# The human placenta and its role in reproductive outcomes revisited

**DOI:** 10.1152/physrev.00039.2024

**Published:** 2025-06-11

**Authors:** Irving L M H Aye, Stephen Tong, D. Stephen Charnock-Jones, Gordon C S Smith

**Affiliations:** 1 Department of Obstetrics and Gynaecology, University of Cambridge, NIHR Cambridge Comprehensive Biomedical Research Centre, Cambridge, United Kingdom; 2 Loke Centre for Trophoblast Research, Department of Physiology, Development and Neuroscience, University of Cambridge, Cambridge, United Kingdom; 3 Wellcome Trust - Medical Research Council Stem Cell Institute, Jeffrey Cheah Biomedical Centre, University of Cambridge, Cambridge, United Kingdom; 4 Department of Obstetrics and Gynaecology, University of Melbourne, Victoria, Australia; 5 Mercy Perinatal, Mercy Hospital for Women, Heidelberg, Victoria, Australia

## Abstract

The placenta performs many key tasks which are essential for healthy growth and development of the human fetus. Placental dysfunction has multiple manifestations but they share the common property of lacking mechanistic understanding of etiology. The clinical consequences of placental dysfunction are a major determinant of the global burden of disease. Currently, the primary clinical method for assessing placental function is ultrasonic Doppler flow velocimetry of the umbilical and uterine arteries. More recently, some biomarkers have emerged which can predict or diagnose placentally-related complications of pregnancy. However, methods for identifying and characterizing placental dysfunction have developed relatively little over the last 20 years and perform poorly, and there remains an absence of disease modifying therapies targeted at the placenta. Understanding disease mechanisms is made more difficult due to the profound differences in pregnancy and placentation comparing humans and the most commonly used laboratory animals, limiting the utility of animal models. Use of omics methods in human samples may yield progress: omics analyses of maternal blood shows promise in identifying better predictors of disease and single cell analyses, including spatial omics of healthy and abnormal placentas, could identify therapeutic targets. Limitations in cellular models of the placenta have been significantly overcome in the last five to ten years by the development of human cell models, including human trophoblast stem cells and organoids, and use of these model systems may allow hypothesis testing experiments in a more clinically relevant context than animal models or immortalized cell lines.

## 1 Introduction

The placenta is an organ like no other. It combines a complex skill set, including gaseous exchange, metabolism and transport of nutrients and waste, and endocrine control of multiple elements of maternal and fetal physiology. Fetal growth and development are wholly dependent on placental function. In the first three months of its existence the placenta grows from a handful of cells in the blastocyst to ˜50 grams of tissue and in the next six months, it increases in size 10-fold. Its final role is to separate from the uterus in a perfectly timed dive to oblivion: too early and the baby dies due to placental abruption, too late and the mother dies from retained placenta and postpartum hemorrhage. Yet it is relatively neglected as a subject of scientific research. How can physiologists study the pancreas, a dullard controlling sweetness and digestion, day after day the same for decades, and ignore the gloriously multitalented and short-lived placenta? They are biographers documenting the blameless life of second violin in a provincial symphony orchestra while eschewing the story of Jimi Hendrix.

As healthy placental function is fascinating, placental dysfunction is devastating, the cause of untold human suffering and death. Pregnancy and neonatal complications directly cause about 10% of all years of life lost due to disease globally (1) and placental dysfunction is central to the pathophysiology of many of the causes, including fetal growth restriction (FGR), preeclampsia, spontaneous preterm birth and stillbirth (2). The placenta also has an important role in determining the risks of non-communicable diseases in adulthood. Women experiencing preterm birth, FGR and preeclampsia during pregnancy are at increased risk of death and morbidity in later life due to myocardial infarction (3) and stroke (4, 5), an area characterized as “Pregnancy as a Window to Future Health” (6). Furthermore, abnormalities of fetal growth and development are associated with diverse lifelong risks for the offspring, an area characterized as the “Developmental Origins of Health and Disease” (DOHaD) (7). Extensive studies in animals confirm that perturbation of the intra-uterine environment results in fetal adaptations which would predispose to conditions such as diabetes, hypertension, ischemic heart disease and cerebrovascular disease (8) and placental function is critically important in the mechanisms mediating these effects (9).

The aims of the present review are to provide an overview of current knowledge of healthy development of the placenta, to outline how placental dysfunction leads to complications of pregnancy, and to describe in vitro and animal models which may provide mechanistic understanding of disease pathophysiology. Moreover, we will outline how understanding normal and abnormal placental function informs tools to assess fetal well-being and could lead to better methods of predicting and preventing pregnancy complications and the development of novel therapeutics to improve outcome in women experiencing complications. Given the wide scope of the review, most of the references to the literature will not involve an in-depth critique, but the reader is referred to review articles which deal with the evidence in more detail. We draw parallels between data from both human and animal studies throughout the paper, but the strengths and weaknesses of the model systems is discussed in detail in section 6.

## 2 Early placental development

### 2.1 Development of the trophoblast cells

The first definitive cell differentiation event in the human embryo is the formation of the trophectoderm which separates it from the inner cell mass in the late blastocyst stage. Around 7-9 days post-fertilization (dpf), implantation is initiated by the trophectoderm attaching to the receptive luminal epithelium of the endometrium. The trophectoderm then transforms into cytotrophoblasts, trophoblast stem cells that can differentiate into the two other major trophoblast cell types: syncytiotrophoblast and extravillous trophoblasts.

**Figure 1:**
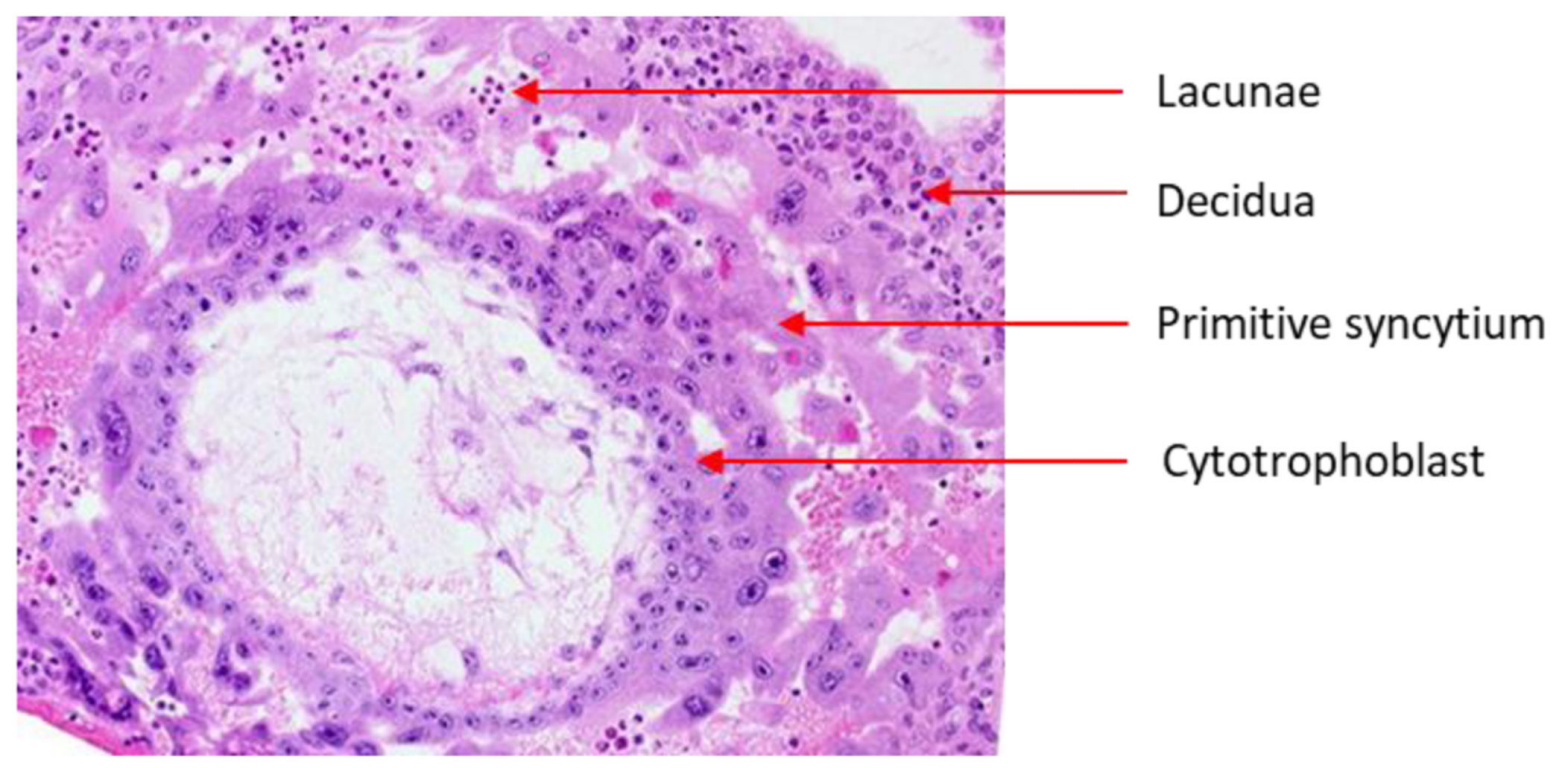
Cross-section of a post-implantation embryo. Histological section of a post-implantation embryo (Carnegie stage 5c, 11-12 dpf) following Hematoxylin and Eosin staining. The cytotrophoblasts have begun to differentiate into the primitive syncytium which invades into the maternal decidua forming lacunae. Images reprinted with permission from the Virtual Human Embryo Project at Louisiana State University (http://virtualhumanembryo.lsuhsc.edu).

**Figure 2:**
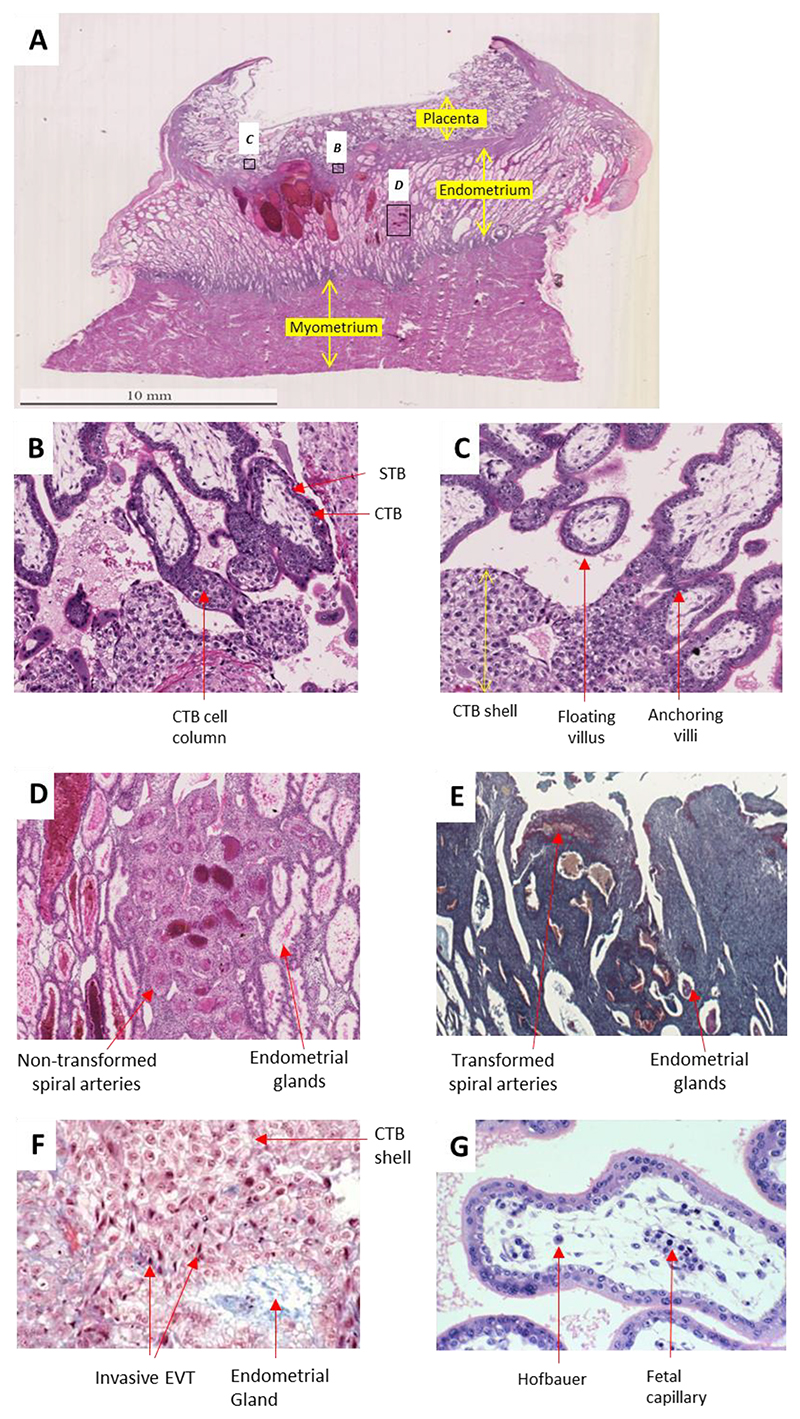
Cross-section of the maternal-fetal interface in early pregnancy. Hematoxylin and eosinstained sections from the Boyd collection of [**A-D, F-G**] a 6-week gestational age specimen (H710) and [**E**] a 10-week gestational age specimen (H630) obtained from pregnant women where hysterectomies were performed. **A** Overview of the uterus with attached conceptus. **B** High magnification image showing cytotrophoblast cell columns migrating towards the decidua. **C** High magnification image showing floating villi and anchoring villi with cytotrophoblasts spreading to form the cytotrophoblast shell. **D** Low magnification image showing non-transformed spiral arteries with narrow bores and surrounding endometrial glands. **E** Low magnification image of transformed spiral arteries showing dilatated vessels with large bores. **F** High magnification image of invasive EVTs migrating from CTB shell. **G** High magnification image showing a villus with a Hofbauer cell in a stromal channel formed by fibroblasts and a fetal capillary with nucleated red blood cells. CTB, cytotrophoblast; EVT, extravillous trophoblast; STB, syncytiotrophoblast. Placenta-in-situ slides from the Loke Centre for Trophoblast Research Boyd Collection, funded by Loke Centre for Trophoblast Research and the Wellcome Trust (215361/Z/19/Z). Licensed under CC BY-NC-SA 4.0. Retrieved 23-Apr-2025 from https://www.trophoblast.cam.ac.uk/Resources/boyd-collection.

The attachment of the trophectoderm to the uterine layer initiates the cytotrophoblasts to differentiate into the primitive syncytium (10), an epithelium made up of a unique trophoblast subtype with both invasive and secretory functions. The primitive syncytium secretes digestive enzymes eroding the uterine stroma, glands, and capillaries, and rapidly expand into the decidua (Figure 1). The resulting cavities, known as lacunae, become filled with blood and glandular secretions from the degraded tissue, providing an early source of nutrition for the conceptus known as histotrophic nutrition. The underlying cytotrophoblasts rapidly proliferate and traverse through the primitive syncytium and form mass elongations known as cytotrophoblast cell columns (Figure 2A-B). The expanding cytotrophoblasts ultimately contact the decidua basalis and spread laterally and merge to encircle the conceptus in a continuous CTB shell (11) (Figure 2C).

Within the cell columns, the cytotrophoblasts in the proximal region remain proliferative but cytotrophoblasts in the distal cell column cease their mitotic activity and differentiate into the highly invasive extravillous trophoblasts (Figure 2F). These cells leave the cell columns and deeply migrate through the decidua (12) and up to the inner third of the myometrium where they fuse to form multinucleated cells referred to as placental bed giant cells (13, 14). A subset of extravillous trophoblasts reach the endometrial spiral arteries and transform into endovascular trophoblasts that migrate down the lumens of the arteries and replace the endothelial cells. The endovascular trophoblasts form aggregates that plug the arteries greatly limiting the maternal blood flow into the intervillous space in the first 10 weeks of pregnancy. This creates a physiologically low oxygen environment that is permissive to trophoblast proliferation (15), angiogenesis (16) and protection against damage from reactive oxygen species (17).

During this stage, the spiral arteries are also extensively remodeled by the endovascular trophoblasts (Figure 2D-E), leading to the loss of elastin and smooth muscle from the arterial walls which are replaced by fibrin, resulting in wide flaccid bores with loss of vasoreactivity (18). The endovascular trophoblast plugs progressively disintegrate after 10 weeks gestation, allowing maternal blood to fill the intervillous space. Remodeling of the spiral arteries has several important consequences on the intervillous blood flow. Dilatation of the artery decreases the speed and pulsatility of the maternal blood flow into the intervillous space reducing the risk of damage to the chorionic villi, and the loss of smooth muscle prevents spontaneous vasoconstriction (19).

The evaginations of trophoblasts that are in direct contact with the maternal blood within the intervillous space are referred to as the floating villi and the sites where the trophoblasts attach to the decidua are referred to as the anchoring villi (Figure 2C). Both the anchoring and floating villi comprise an outer layer of syncytiotrophoblast surrounding the cytotrophoblast core. The floating chorionic villi represent the functional units of the mature placenta, responsible for the maternal-to-fetal exchange of nutrients, electrolytes, gases, and waste. These villi also secrete numerous proteins into maternal circulation some, such as hCG have well recognized endocrine functions and regulate maternal physiology. Many others have poorly described functions but nonetheless have potential as biomarkers reflecting placental function.

### 2.2 Non-trophoblast cells of fetal origin in the human placenta

While the trophoblasts make up the villus portion of the placenta, the stromal core of the villi contains a variety of cells including fibroblasts, and immune and vascular cells which are thought to be generated from the extra-embryonic mesenchyme (20). Vasculogenesis is the process of new blood vessel development from progenitor cells. This process occurs in the first 4 weeks of pregnancy where mesenchymal stem cells differentiate into hemangioblastic cell cords (21, 22). The cords transform into endothelial tubes that elongate due to proliferation of existing cells. Stromal cells such as pericytes are recruited and the simple tubes are transformed into a vascular network. This is further extended by the growth of new vessels from the existing ones i.e. angiogenesis (23). This process is regulated by paracrine signals, where VEGF-A plays a critical role. VEGF-A is initially secreted by the cytotrophoblasts in the first few weeks of pregnancy followed by the placental-resident macrophages (Hofbauer cells) and mesenchymal cells.

The existence of fetal immune cells in the human placenta was first reported by Hofbauer in 1903 (24) but their identity as macrophages was only confirmed in 1970 (25). The eponymous Hofbauer cells are believed to be the only immune cell population of fetal origin that exist in a healthy placenta. Hofbauer cells first appear around 18 days post-conception (26). These cells were previously believed to originate from the yolk sac, but a later study demonstrated that Hofbauer cells arise *de novo* within the placenta through primitive hematopoiesis (27). The function of Hofbauer cells remains largely unknown but they may aid in trophoblast differentiation and vascular remodeling by producing growth factors, cytokines, and matrix metalloproteinases (28). Hofbauer cells express high levels of TLR6 and exhibit potent microbicidal activity (28), indicating a protective capacity against transplacental infections. However, Hofbauer cells are susceptible to viral infections including HIV and Zika virus as they express entry receptors for these viruses and viral replication inside these cells has been reported (29, 30).

### 2.3 Maternal immune cells at the maternal-placental interface

Maternal immune cells do not cross the placental barrier in healthy pregnancies. However, different subsets of unique leukocytes accumulate at the maternal – placental interface and interact with the syncytiotrophoblast and extravillous trophoblasts that are in direct contact with maternal blood and decidual tissues respectively. In the first trimester decidua, the leukocyte population in this tissue is made up of: specialized natural killer cells known as decidual natural killer cells (50-70%), macrophages (20-30%), T cells (˜10%) and dendritic cells (˜1%) (31–33). As pregnancy progresses, the local immune cell population changes significantly with decidual natural killer cells accounting for less than 50% of the leukocyte population at term, while T cells (>50%) become the dominant group, accompanied by proportional changes in the other leukocyte populations.

Decidual natural killer cells differ from their peripheral counterparts in both morphology and phenotype. They proliferate and differentiate within the uterine mucosa during the menstrual cycle and acquire cell surface expression of killer cell immunoglobulin-like receptors (KIRs), leukocyte immunoglobulin like receptor B1 (LILRB1) and CD94-NKG2-A/C (referred to as NKG2A or NKG2C). These receptors interact with various forms of human leukocyte antigens (HLAs): KIRs bind to HLA-C, LILRB1 binds to HLA-G, and NKG2A (inhibitory) and NKG2C (activating) bind to HLA-E. Together, these receptors regulate the effector functions of natural killer cells, including the production of chemokines and angiogenic factors that promote extravillous trophoblast differentiation and migration (34). Additionally, decidual natural killer cells contribute to spiral artery remodeling by degrading extracellular matrix components and displacing smooth muscle cells (35). Each decidual natural killer cell expresses a pair of KIRs that are highly variable, and each KIR may be either activating or inhibiting, and thus the balance of these receptors determines natural killer cell function. Genes that encode both KIR and HLA-C are highly polymorphic and thus the different combinations of these genes lead to variable activation or inhibition of decidual natural killer cells. Genetic studies suggest that the combination of maternal KIRs and fetal HLA-C can alter the risk of developing placenta-related pregnancy disorders such as preeclampsia (36), discussed further in *Section 5.4.2*.

Cytotoxic T cells make up ˜45% of the T cell population in the first trimester decidua. Despite their cytotoxic nature in peripheral tissues, these T cells do not trigger lytic responses to trophoblasts. The absence of any HLA molecules on syncytiotrophoblast that is in direct contact with maternal blood avoids systemic immune recognition by T cells. Cytotoxic T cells in the decidua show signs of exhaustion, such as high expression of inhibitory immune checkpoint proteins and suppressed activity (37). While their exact role remains unclear, they are believed to assist in immune surveillance and pathogen clearance at the maternal-fetal interface as the placenta grows (38). Regulatory T (Treg) cells are another subset of T cells present at the placental interface and their differentiation is induced by TGFβ secreted from extravillous trophoblasts. Both decidual cytotoxic T cells and natural killer cells are regulated by Treg cells that are normally involved in suppressing the cytotoxic activity response thus ensuring immune tolerance to the allogenic trophoblast.

### 2.4 Single cell-RNA-sequencing to examine cellular heterogeneity in the human placenta – a critique of existing literature

In recent years there has been a technological revolution in the way tissues can be analyzed – the so-called single-cell -omic methods. Analysis of large number of single cells, particularly single cell RNA sequencing (scRNAseq) has transformed the approach to many biological questions (39). This allows diverse cell types to be identified and, unlike previous methods, enables the near-complete repertoire of transcripts in a cell to be analyzed. Thus, the signals a cell sends and those it receives and its response can be analyzed. For example, the immunosuppressive action of T helper 2 (Th2) cells is mediated by their steroidogenic action (40). This is a rapidly moving field with frequent descriptions of new and improved methods. Nonetheless, the availability of commercial platforms has made the technology readily accessible (41–43). scRNAseq allows for the simultaneous identification of new cell types and the characterization of their potential functions. It can also reveal the likely regulatory networks that govern differentiation pathways of divergent cell populations within a tissue (44–46).

While these approaches are exciting, considerable caution is needed when considering the placenta. The major challenge with using single-cell based approaches to analyze the placenta is that the placenta is not composed of individual cells – the surface that is in contact with maternal blood is a syncytium with multiple nuclei embedded in a single cytoplasmic mass with no cell boundaries between the nuclei. Therefore, when evaluating papers which describe “single cell” analysis of the placenta, one needs to carefully consider what has actually been analyzed. For example, some papers reporting such analysis include syncytial “cells” in their figures but do not discuss the under-representation of syncytiotrophoblast in their datasets, they simply assume they have analyzed all the cells in the sample. Indeed, in the figures presented by Pique-Regi et al the syncytiotrophoblast are barely visible (47); Tsang et al do detect and discuss the syncytiotrophoblast but these are always rarer than cells from the other trophoblast lineages (48). Similarly, syncytiotrophoblast “cells” are less than 10% of the total trophoblast as reported by Suryawanshi et al (49). This is in marked contrast to the data obtained by stereological methods in which the cells (or nuclei) are counted in situ and therefore are not affected by selective losses during cell isolation. These methods report that at all stages of gestation approximately 90% of the villous trophoblast nuclei are within the syncytium (50). While the stereological approach is clearly unable to provide any data on the molecular characteristics of the cells and is only useable when cells can be unambiguously identified microscopically, it is robust to the confounding effects of biased cell isolation. The reliable and unbiased isolation of single cells for scRNAseq is an often-overlooked challenge in the field (51).

Another potential bias that needs to be considered is that tissue heterogeneity can profoundly affect the proportion of a given cell type that is present in a sample. In the placenta the number of smooth muscle cells varies greatly with the sampling site. There are very few at the basal plate but many more in the deeper portions closer to the chorionic plate, which complicates comparisons between different placentas.

The interaction between maternal decidual and fetal trophoblast cells has long been recognized as important and this likely to be tractable to scRNAseq analysis. As there are no syncytiotrophoblast in the decidua this is much less likely to be confounded by cell isolation bias. This field has developed rapidly – the first paper only analyzed 87 transcriptomes (i.e. cells) obtained from single cells collected from 2 term placentas (52) and only a year later the detailed analysis of about 70,000 cells was reported (53), including a detailed analysis of the immune cells present at the feto-maternal interface which characterized three major subsets of decidual natural killer cells (dNK1-3). Based on the ligands and receptors expressed by these and distinct extravillous trophoblasts subpopulations it is likely that one subset (dNK3) regulates extravillous trophoblasts invasion. The focus of this work was the dialogue at the maternal-fetal interface (ie in the decidua) and it identified mechanisms which generate “a physiologically peaceful decidual environment” (54). This is a good example of the power of scRNAseq analysis, but it does not satisfactorily address the function of the syncytiotrophoblast.

Fortunately, there are methods that alleviate the shortcomings mentioned above. Specifically, the analysis of isolated nuclei (rather than single cells (55, 56) and use of spatial transcriptomics (57). The latter has the great advantage of preserving the spatial relationship between cells and providing information on their transcriptomes. These two methods have been elegantly applied to the placental bed to reveal the mechanism underlying trophoblast invasion in the first trimester (58). This work characterizes distinct populations of trophoblast and their final cell states. Spatially resolved analysis of the implantation site – from the floating villi to the underlying myometrium – allowed the characterization of placental bed giant cells, endovascular trophoblast and the syncytiotrophoblast. As spatial information is preserved the perivascular maternal stromal cells and the invading extravillous trophoblasts can be identified and the possible mediators (Ephrin B1 for example) of their interaction defined (59). This focus on spiral artery remodeling has been considerably extended and the temporal progression modelled by the integration of spatially resolved mass-spectrometry based imaging and co-registered spatial transcriptome analysis (59). While spatial transcriptomics is a very powerful technique, the single cell methods have the advantage that other -omic methods can be applied simultaneously to the same cell. The most common is chromatin accessibility (ATACseq) but other methods are also in use (60, 61). Spatial versions of these modalities are being developed but as these are technically challenging and are often limited by resolution, single cell methods have greater scalability and flexibility.

### 2.5 Molecular mechanisms regulating the trophoblast lineage

Development of the cells within the trophoblast lineage is dependent upon specification of the trophoblast stem cell population, and regulation of their self-renewal or differentiation. Regulation of trophoblast fate is determined by specific combinations of transcription factors that cooperate with additional transcriptional and epigenetic machinery to function as a network. Thus, the stem versus differentiated fate of trophoblasts depends on coordinated regulation of gene expression patterns whereby a group of transcription factors are activated while others are repressed. Any imbalance in this regulation can lead to alterations in self-renewal or differentiation leading to defective placentation. The importance of trophoblast stemness on organism development was elegantly illustrated in mice whereby knockout of the genes associated with embryonic lethality and placental dysmorphologies *in vivo*, are commonly associated with impaired trophoblast stemness or differentiation *in vitro* (62).

The molecular regulation of trophoblast stemness has largely been uncovered in mice and whether much of these mechanisms are conserved in humans remain to be verified. It is important to note that there are considerable species differences in the molecular mechanisms regulating the trophoblast lineage between humans and mice. For example, CDX2 activity and TGF-β signaling promotes trophoblast stemness in mice (63, 64); whereas CDX2 is lowly expressed in human trophoblast stem cells and TGF-β inhibition (rather than activation) increases trophoblast stemness in humans (65). Therefore, we focus our review on the transcriptional and cell-signaling mechanisms that either have conserved roles in humans and mice or human-specific roles. The role of epigenetics on trophoblast development is discussed in depth in a recent issue of this journal (66).

#### 2.5.1 Cytotrophoblast self-renewal

Following implantation, cytotrophoblasts emerge from the trophectoderm, and these cells represent the trophoblast stem cell population. However, it is important to note that there are likely several sub-populations of cytotrophoblasts with limited potency which represent the intermediate populations that appear during the differentiation process. For example, extravillous trophoblasts differentiate from a subset of cytotrophoblasts in the cell columns during early pregnancy whereas the villous cells differentiate into syncytiotrophoblast throughout gestation. Indeed, single cell RNA-seq studies have reported three major sub-populations of cytotrophoblasts (54, 67). One population represents cytotrophoblasts undergoing rapid self-renewal with high expression of cell cycle genes and transcription factors, such as MYC, which drive proliferation. The second is an intermediate population of cells that have exited the cell cycle with upregulation of genes associated with differentiation towards the syncytiotrophoblast lineage. The phenotype of the third population is less clear to infer based on the transcriptome (67) but may represent a quiescent population which has previously been reported as the ‘side population’ of cytotrophoblasts that exist throughout pregnancy (68). The terms trophoblast stem cells and cytotrophoblasts have been used interchangeably in the literature, and thus in this review we refer to all trophoblasts with the capacity to differentiate into extravillous trophoblasts and/or syncytiotrophoblast as cytotrophoblasts.

Given the inaccessibility of the human tissue to sampling during the period of post-implantation development, little is known about the transition from trophectoderm to cytotrophoblast. Several transcription factors associated with trophectoderm differentiation are retained in the cytotrophoblasts where they promote stemness. For example, GATA2/3, TFAP2C and TEAD4 are expressed in both the trophectoderm and cytotrophoblasts (69), and the knockout/knockdown of these genes reduces stemness and results in loss of viability in cytotrophoblasts (70, 71). Of these, GATA2/3 and TFAP2C likely play a role at the earliest stage of the trophoblast lineage as they have been shown to function as pioneer transcription factors in several developmental contexts, for example, during gastrulation (72) or during the development of the hematopoietic and cardiac systems (73). Pioneer transcription factors bind directly with nucloesomal DNA and promote chromatin opening and recruitment of additional transcriptional regulators to initiate gene network changes (74). In mice, GATA2 and GATA3 demonstrate redundancy due to a large overlap in their regulated genes which include those involved in cell cycle and proliferation as well as cytotrophoblast genes ELF5 and TFAP2C (75).

TEAD4 is a component of the Hippo signaling pathway that directly promotes the expression of genes involved in cell cycle regulation and proliferation while simultaneously inhibiting genes regulating syncytiotrophoblast and extravillous trophoblast differentiation (76). Knockout of the TEAD4 transcriptional co-activators YAP1 (77) or WWTR1 (78) produces the same phenotype as TEAD4 knockout cells, demonstrating the importance of the Hippo pathway in regulating trophoblast stemness. Interestingly, TEAD4 regulates trophectoderm formation in mouse embryos but not human embryos (79), but TEAD4 is essential for the viability of human cytotrophoblasts (71). This suggests that, unlike mice, TEAD4 in humans does not play a vital role in the earliest stages of trophoblast development but is nevertheless critical for trophoblast stemness. Low cytotrophoblast expression of TEAD4 or its co-activators YAP1 and WWTR1 is associated with pathological pregnancies such as preterm birth, severe preeclampsia, and recurrent miscarriage (76, 78, 80).

Other transcription factors regulating stemness appear in the cytotrophoblasts but not the trophectoderm. In humans, ELF5 is expressed in the cytotrophoblasts but absent in the trophectoderm although its expression is retained in both compartments in mice (81). It is highly expressed in the first trimester human placentas from 8 – 12 weeks and declines thereafter (82). The ELF5 promoter is highly methylated and consequently silenced in embryonic stem cells restricting its expression to the trophoblast lineage (82, 83). Similarly, placental ELF5 DNA methylation increases with gestation, correlating with its decline in mRNA expression (82). In mouse trophoblast stem cells (mTSCs), ELF5 knockdown decreases stemness and promotes precocious differentiation without altering apoptosis (84). Moreover, ELF5 knockout mice exhibit an ablated extraembryonic ectoderm, a region which subsequently forms the fetal portion of the placenta (85). Although ELF5 is expressed in the mouse trophectoderm, its knockout does not impair trophectoderm development or implantation suggesting that ELF5 plays a role only once the cytotrophoblasts are developed. In mTSCs ELF5 binds to EOMES and TFAP2C to form a regulatory network that coordinates transcription of additional trophoblast stemness genes (86). Whether similar mechanisms operate in humans is unclear, especially given that EOMES is lowly expressed in cytotrophoblasts (65).

MSX2 is a transcription factor that maintains trophoblast stemness only in humans (87). Interestingly, MSX2 does not regulate the transcription of known trophoblast stemness genes. Rather, it is a repressor of the syncytiotrophoblast lineage and its knockdown leads to precocious differentiation into syncytiotrophoblast while overexpression prevents differentiation. Mechanistically, MSX2 binds directly to syncytiotrophoblast-associated genes as well as the cBAF chromatin remodeling complex to inhibit their activity. Therefore, reductions in MSX2 during differentiation leads to cBAF de-repression and activation of syncytiotrophoblast genes.

The above transcription factors are just few examples to demonstrate the complex network that operates within cytotrophoblasts. Regulation of trophoblast stemness is thus dependent on factors that drive self-renewal (i.e. proliferation) as well as others that inhibit differentiation.

#### 2.5.2 Differentiation into syncytiotrophoblast

Syncytiotrophoblast is a multinucleated epithelium that forms the placental interface that is in direct contact with maternal blood in the intervillous space. As the maternal-fetal interface, syncytiotrophoblast play an important role in mediating the exchange of nutrients, gases and waste to support fetal development. Additionally, the syncytiotrophoblast produces and secretes large quantities of hormones that play critical roles in regulating the maternal physiological adaptations to pregnancy. The syncytiotrophoblast is also devoid of HLA class I molecules (88) aiding its escape from alloimmune recognition.

The syncytial layer occupies the largest surface area of the placenta. At term, syncytiotrophoblast contain around 58 billion nuclei, approximately 10-fold higher number of nuclei than cytotrophoblasts (50). Despite this, scRNA-seq studies to date have identified only one syncytiotrophoblast population in both first trimester (54, 67) and term placentas (52, 89). This is a gross underestimation since, as described above, the multinucleated epithelium is not amenable to standard scRNA-seq methods that require isolation of single cells. Single-nuclei RNA-sequencing (snRNA-seq) offers an alternative approach to interrogate syncytiotrophoblast populations by examining their nuclei. This approach has now been applied to human placentas and the proportion of syncytiotrophoblast is more accurately reflected (90). snRNA-seq of the mouse labyrinthine placenta similarly identified multiple syncytiotrophoblast populations (91) compared to the single population identified by scRNA-seq (92).

Throughout gestation, the syncytial layer undergoes a highly regulated turnover to replace aged nuclei and organelles which are shed as syncytial knots into the maternal circulation. Syncytial renewal is facilitated by the fusion of the underlying cytotrophoblasts into the overlying syncytia. This process is referred to as syncytialization, and requires complex coordination of multiple cellular events, and can be assessed *in vitro* by measuring secretion of hormones such as human chorionic gonadotrophin (hCG), human placental lactogen and progesterone, and observing intercellular fusion. One of the key initial events in syncytialization is the rise in intracellular cyclic AMP (cAMP) mediated by adenylate cyclase. Various extracellular signals such as hormones, cytokines and growth factors as well as the drug forskolin, induce cAMP production via adenylate cyclase. The rise in cAMP activates protein kinase A leading to the phosphorylation of various proteins including the transcription factor cAMP response element binding protein (CREBP). CREBP is an important regulator of the syncytiotrophoblast program and promotes the transcription of the cell cycle inhibitor p21 and the transcription factor glial cell missing-1 (GCM1). p21 causes cells to exit the cell-cycle, halt proliferation and enter G0 arrest (93). Furthermore, p21 interacts with GCM1 to drive the transcription of genes encoding fusogenic proteins which include syncytins, cadherins, gap junction proteins and hormones such as hCG (94–96).

Amongst the fusogenic proteins, syncytins play an important role that uniquely occurs only in the placenta. Syncytin-1 is encoded by the endogenous retrovirus W-1 (*ERVW1*) and Syncytin-2 by *ERVFRD1*. These genes are remnants of historical retroviral infections that integrated into the human genome and have been inherited by humans over successive generations. Syncytin-1 and -2 are cell surface proteins that bind to plasma membrane receptors ASCT2 and MSFD2 respectively promoting membrane bending, merging and formation of the fusion pore (95, 97). Forced expression of Syncytin-1 or -2 in non-trophoblast cells is sufficient to trigger cell-cell fusion but these fused cells are unstable, continue to divide, and form defective syncytia (93), suggesting that fusion alone is not sufficient for syncytialization.

The number of syncytial nuclei increases exponentially during gestation but no mitotic figures are observed in these nuclei because the increase is driven by continual fusion of the underlying cytotrophoblasts. However, some stain positively for PCNA (proliferating cell nuclear antigen), suggesting recent fusion and others will necessarily be older. Consistent with this, there is considerable microscopic heterogeneity among the nuclei within the syncytium. The number of transcriptionally active nuclei increases with the majority being active (98).

Nuclei with highly condensed chromatin aggregate into syncytial knots and these become more frequent in late gestation. In pregnancies complicated by preeclampsia the increase is exacerbated, and the changes are referred to as Tenney-Parker changes (99). These nuclei are transcriptionally inactive, but they have increased staining for 5’-hydroxymethylcytosine, a mark for transcriptional activation (100). Chromatin condensation is typically associated with apoptosis, and it has been suggested that there is increased apoptosis in syncytial knots and in FGR and preeclampsia (101). However, this may not be the case as apoptotic cytotrophoblasts can be surrounded by the syncytium and hence it may appear that a syncytiotrophoblast nucleus is undergoing apoptosis, but it is in fact a cytotrophoblast. These authors conclude that caspase-mediated apoptosis was not detectable in regions with intact syncytiotrophoblast (102). Thus, the process of cell death is more nuanced than previously thought and as the placental syncytial surface is unusual so it is possible that the process underlying syncytiotrophoblast maturation may be different to what is typically observed in other tissues (103).

#### 2.5.3 Differentiation into extravillous trophoblasts

Extravillous trophoblasts originate from the anchoring villi and their differentiation can be broadly categorized into two stages. The first involves the formation of the extravillous trophoblast progenitors followed by the invasive extravillous trophoblasts. Extravillous trophoblast progenitors are formed in the proximal cell columns and are characterized by transient proliferation. At the distal portions of the cell columns, the extravillous trophoblasts lose their proliferative activity and cell-cell contact, leading to the invasion into the maternal decidua and beyond. Shallow extravillous trophoblasts invasion and spiral artery remodeling are implicated in preeclampsia and FGR, suggesting these conditions may arise from a defect in the extravillous trophoblasts differentiation program. Combined spatial and single-cell transcriptomics analysis identified four populations of extravillous trophoblasts (named EVT-1 to 4). EVT-1 and EVT-2 emerge from the proximal and distal cell columns respectively (58). EVT-1 represent the proliferative cells while EVT-2 cease their proliferative activity and bifurcate into two invasive extravillous trophoblast populations: the interstitial extravillous trophoblasts detected within the maternal decidua and myometrium, and the endovascular extravillous trophoblasts (including trophoblast plugs) found within the spiral arteries.

Extravillous trophoblast progenitor formation is highly influenced by the interactions between the cytotrophoblasts on cell columns and neighboring cells through paracrine signaling. The Notch1 pathway is one such example of paracrine signaling in the proximal cell columns that promotes extravillous trophoblast progenitor development (104). Notch signaling requires direct cell-cell contact whereby one cell expresses a ligand and interacts with an adjacent cell that expresses the receptor. Ligand binding results in proteolytic cleavage of the receptor to release Notch1 intracellular domain (N1ICD) that translocates to the nucleus and functions as a transcriptional co-activator (105). Overexpression of N1ICD in cytotrophoblasts induced the expression of cell cycle proteins and E-cadherin to promote extravillous trophoblast progenitor proliferation and cell column attachment (104).

Another mechanism regulating the differentiation into an extravillous trophoblast progenitor is the local secretion of soluble paracrine factors. Decidual stromal cells secrete Neureglin-1 which binds to its target receptor ErbB3 on progenitor extravillous trophoblasts. This triggers the heterodimerization of ErbB3 with ErbB2 leading to their phosphorylation and activation of the downstream mitogen activated protein kinase (MAPK) pathways Erk and phosphoinositol-3-kinase (PI3K)/Akt. Activation of this pathway in placental explants leads to stabilization of the cell columns by protecting it against apoptosis and thus promoting the formation of extravillous trophoblast progenitors but does not affect the migration of these cells (106).

Wnt signaling is another major pathway that is involved in multiple aspects of extravillous trophoblast development. Cytotrophoblasts express Wnt receptors on the extracellular surface which bind to Wnt ligands either secreted by the underlying fibroblasts (paracrine signaling) or by the cytotrophoblasts themselves (autocrine signaling) (107). In the canonical Wnt pathway, this culminates in the nuclear translocation of β-catenin. In the nucleus, β-catenin interacts with the LEF/TCF family of transcriptional factors which in turn recruit additional cofactors, including the chromatin modifiers CBP/p300 and BRG1 (107). While Wnt signaling is initially required for cytotrophoblast stemness, loss of Wnt activity triggers the formation of extravillous trophoblast progenitors (108). However, as extravillous trophoblasts migrate from the cell columns, Wnt signaling is activated once again (109) where it promotes extravillous trophoblast invasion (110, 111).

Differentiation of extravillous trophoblast progenitors into invasive extravillous trophoblasts is regulated by another secreted protein TGFβ1 (109). The binding of TGFβ1 to its receptor leads to nuclear translocation and activation of SMAD3. This results in downregulation of MYC to cease proliferation, and upregulation of fibronectin and the degradative enzymes diamine oxidase and pregnancy-associated plasma protein A2 (PAPPA2) to promote extracellular matrix (ECM) remodeling associated with cell invasion. Upregulation of PAPPA2 may also result in the cleavage and release of insulin growth factors (IGFs) from IGF binding proteins (IGFBPs) produced in the decidua to promote placental growth. The decidua plays an important role in regulating this pathway as decidual stromal cells and decidual macrophages are rich sources of TGFβ ligands (112).

**Figure 3:**
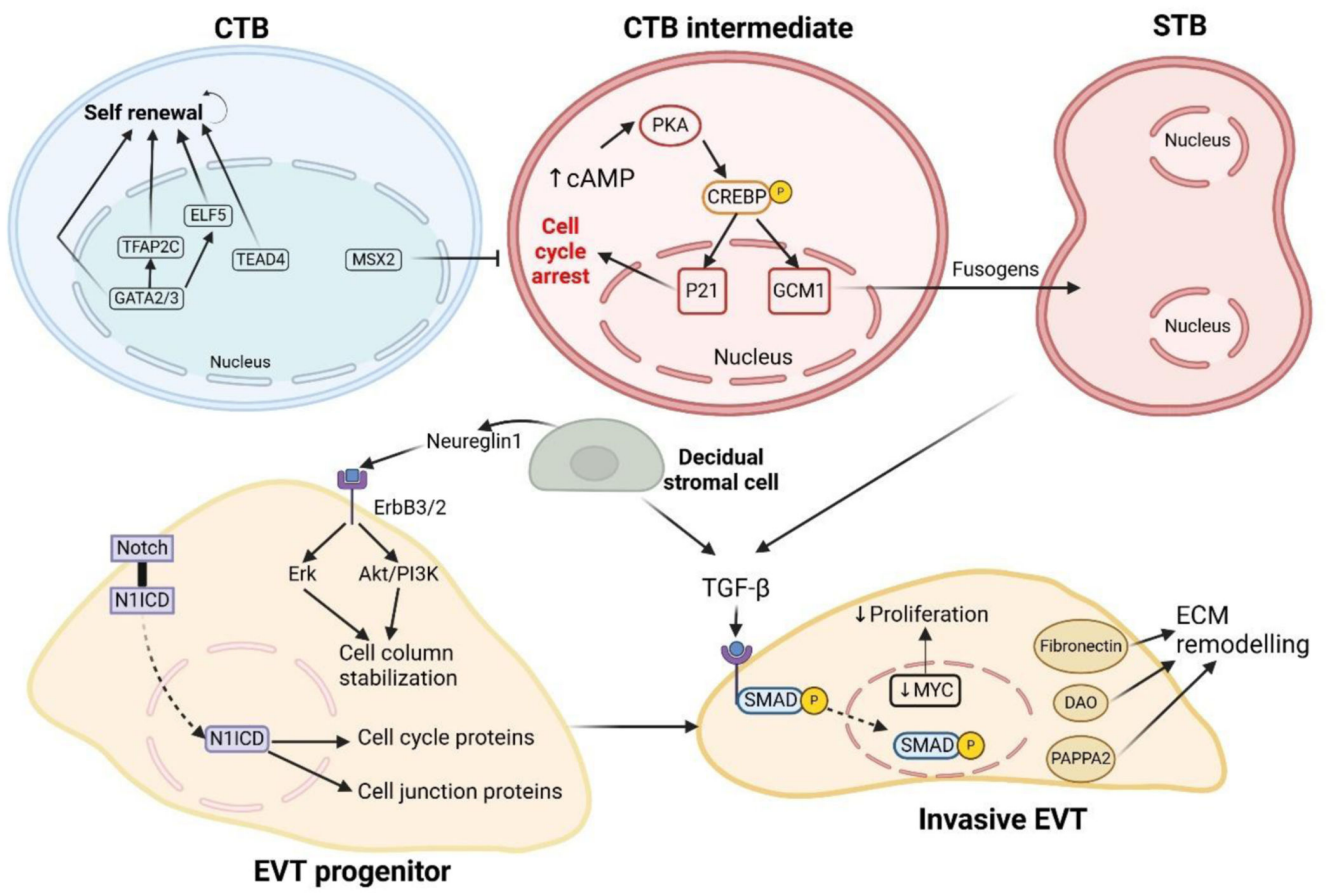
Overview of molecular pathways regulating trophoblast self-renewal or differentiation. The illustration provides an overview of the molecular pathways discussed in the review and is not an exhaustive list. CTB, cytotrophoblast; EVT, extravillous trophoblast; STB, syncytiotrophoblast. *Created using images from a licensed Biorender.com*.

As the extravillous trophoblasts migrate away from the cell columns, they undergo genome amplification via endoreduplication to become polyploid cells (113). The frequency of polyploidy in extravillous trophoblasts increases progressively with the extent of invasion with approximately 60% polyploid cells in the distal columns rising to 80% of extravillous trophoblasts in the decidua (114). Interestingly, *in vitro* differentiation of pure cytotrophoblasts into extravillous trophoblasts does not recapitulate the polyploid phenotype (115) seen *in vivo* or in placental explants (113) suggesting that additional signals from the environment may play an important role. The biological significance of polyploidization remains unclear but it has been hypothesized that the acquisition of multiple sets of chromosomes may buffer extravillous trophoblasts against genotoxic damage caused by reactive oxygen species as they migrate towards oxygen-rich areas of the decidua (116). An overview of the molecular pathways regulating trophoblast development is illustrated below (Figure 3)

## 3 Normal placental structure and function

### 3.1 Placental types

The placenta has evolved independently in a range of species including cartilaginous and bony fish, amphibians, squamate reptiles and mammals. It shows the most interspecies variation of any organ with an extraordinary degree of variation in both gross and fine structure (117–119). This variation is seen in the overall shape of the placenta, the shape and type of interdigitations between the fetal and maternal tissue, the number of cellular layers between the fetal and maternal blood and finally, the direction of flow of the two circulations. Following this classification scheme the human placenta is of the discoid, villous, hemochorial type with multivillous exchange (118, 119).

### 3.2 Villus structure

**Figure 4:**
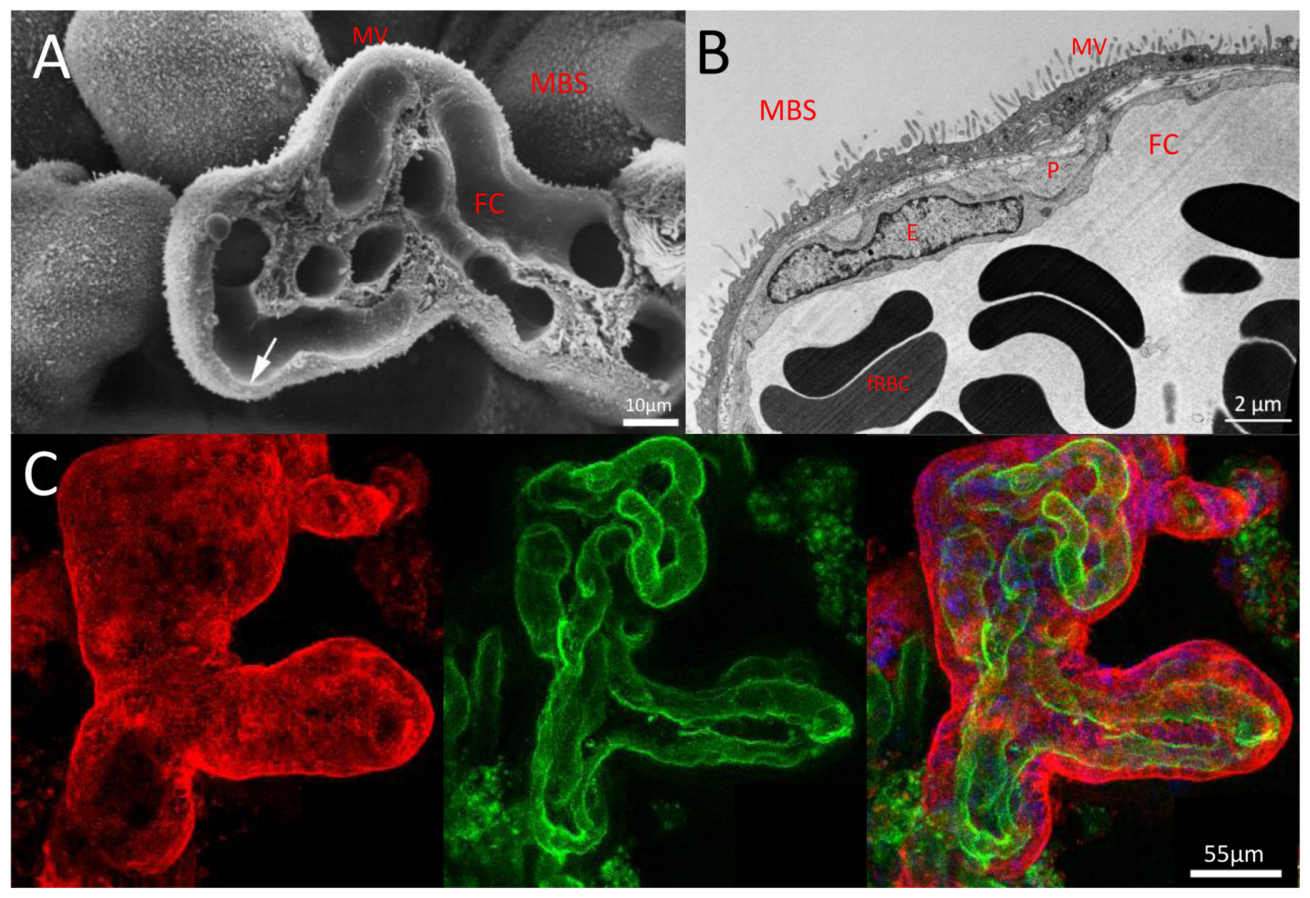
Human placental terminal villi at term. **A)** Scanning electron micrograph of a freeze-cracked human placental villus. The villus has been cracked open to reveal the capillaries inside. The syncytiotrophoblast surface in contact with maternal blood is covered in microvilli. The thin barrier at the vasculosyncytial membrane is arrowed. **B)** Transmission electron micrograph of a terminal villus. The microvilli on the surface of the syncytiotrophoblast are visible. **C)** Confocal images of perfused human villi; syncytiotrophoblast is stained red, endothelial cells green and nuclei blue. MBS maternal blood space; MV micro villi; FC fetal capillary; fRBC fetal red blood cell; E endothelial cell nucleus and P pericyte. Image **A** is from Charnock-Jones and Burton 2000 (16) and **B** was provided by GJ Burton, samples in **C** are those described in Mayo et al 2016 (125).

The human placenta is of the villous hemochorial type - that is, it has a tree-like branching structure that ends in finger-like projections, bathed in maternal blood (Figure 4). The outer syncytial surface is in direct contact with the maternal blood and is optimally placed to carry out maternal-fetal exchange of nutrients and gases.

The larger stem villi contain fetal arteries and veins which act as conduit vessels to carry fetal blood to the sites of feto-maternal exchange in the terminal villi. The stem villi branch to form mature intermediate villi and the vessels also become smaller containing arterioles and venules. The intermediate villi serve to connect the conduit vessels in the stem villi to the exchange vessels – the capillaries in the terminal villi. Terminal villi are the functional units of exchange, and they have a structure specifically adapted for this role. They arise as grape-like protrusions from the surface of the mature intermediate villi and at least 50% of their volume is occupied by the lumens of capillaries and dilated sinusoidal loops. The sinusoids, which can be up to 50µm in diameter, often form on the outer aspect of a capillary bend and the dilation brings the endothelium very close to the overlying syncytiotrophoblast. Indeed, the sinusoids appear to press against the syncytiotrophoblast and cause this to protrude from the villous surface. This leads to the displacement of the syncytial nuclei and organelles and thinning of the syncytiotrophoblast. The two basement membranes beneath the endothelial and trophoblast often fuse resulting in a vasculosyncytial membrane that is 1-2µm thick (120–122).

However, it should be noted that although the human placenta is of the hemochorial type (in which the trophoblast surface is bathed with maternal blood) in the first trimester of pregnancy the exchange mechanism is markedly different. During this period, the villi are not in contact with maternal blood but rather with a plasma exudate containing lipid and carbohydrate-rich secretions from the endometrial glands (123, 124).

### 3.3 Placental function

#### 3.3.1 Transfer and transport

The placenta carries out the functions normally performed by the lungs, intestines, and kidneys as it is the sole mediator of the transfer of oxygen, nutrients and waste products between mother and fetus. These molecules must cross a barrier composed of the continuous syncytiotrophoblast layer and the fetal endothelial cell layer. The syncytiotrophoblast layer has two polarized plasma membranes, the fetal facing basement membrane and the maternal facing microvillous membrane. As the primary placental barrier is a continuous syncytial monolayer there are no cell-cell junctions through which molecules could pass. However, there has long been evidence that passive diffusion of hydrophilic molecules with molecular weights up to 5,000 Daltons occurs and Bain et al conclude that as the “syncytiotrophoblast does not have any paracellular channels and all aqueous pores must therefore be transcellular” (126). Direct evidence for such transcellular channels has been lacking. However, serial block-face scanning electron microscopy allows reconstruction of 3-D tissue structure (127). This has been used to reconstruct placental ultrastructure in three dimensions and demonstrates that trans-syncytial nanopores are present in the syncytiotrophoblast layer. These membrane-bound pores directly connect the maternal and fetal facing sides of the syncytiotrophoblast (128). The formation and regulation of these pores is poorly understood but they provide a pathway for paracellular diffusion between the mother and fetus. They therefore offer a possible route for the transfer of small molecules such as drugs or toxins and potentially particulate material such as that found in the products of combustion (129).

For small molecules such as oxygen which are lipid soluble and are transferred by diffusion, the rate of transfer is determined by the exchange surface area, barrier thickness and concentration gradient. As rate of diffusion is inversely proportional to the barrier thickness (Fick’s law), the surface area and thickness of the vasculosyncytial membrane are important factors in determining exchange capacity. The sinusoids are the main sites for gaseous exchange which is facilitated by the thin barrier, and computational modelling shows that blood velocity is reduced, and transit time increased at these sites (121, 130, 131). There is a dramatic increase in the number of terminal villi from about 20 weeks in gestation (hence an increase in the sinusoidal surface area) and a progressive decrease in the mean villous membrane thickness (132). *Ex vivo* manipulation of the perfusion pressure of the sinusoids shows that the thickness of the interhaemal membrane is dependent on the pressure differential between the fetal and maternal circulations (133). As these are key sites for exchange, processes that alter the number or characteristics of the terminal villi, including the thickness of the vasculosyncytial membranes, have the potential to influence exchange capacity. At term, the diffusion distance of the vasculosyncytial membrane is estimated to be 2–3 µm (134), the placental exchange area around 11 m^2^ (135) and maternal incoming blood flow rate 500-700 ml/min (136).

Transport via passive diffusion alone is inadequate for the placenta to meet its functional and metabolic needs, let alone that of supporting fetal growth. Consequently, the placenta has evolved numerous facilitated transport systems that enable specific and high-capacity transfer of nutrients. These include over 25 amino acid transport systems which mediate the transplacental passage of amino acids (137) and at least seven isoforms of glucose transporters which transfer other carbohydrates (e.g. fructose and galactose) in addition to glucose (138).

Most fatty acids in the maternal plasma are esterified in triglycerides that cannot freely cross cell membranes. Therefore, they are initially metabolized by lipoprotein lipases on the syncytiotrophoblast microvillus membrane which breakdown triglycerides into fatty acids prior to uptake via fatty acid transfer proteins, fatty acid binding proteins and fatty acid translocases. Additionally, cytosolic fatty acid binding proteins traffic fatty acids to organelles to be mobilized for energy production or fetal transfer (139).

Placental transfer of macromolecules such as micronutrients, serum proteins and immunoglobulins (Ig) is mediated by endocytic uptake at the syncytiotrophoblast plasma membrane. Endocytosis may be ligand-specific via receptor-mediated process that utilizes clathrin and caveolin proteins to form coated vesicles, or receptor-independent. Receptor-mediated endocytosis (RME) has been recognized for its role in placental uptake of transferrin-bound iron, lipoproteins, albumin, and viruses including Zika virus (140). RME is also responsible for the placental transfer of maternal IgG to the fetus, the only antibody class that is significantly transferred across the placenta (141). Macropinocytosis, a form of nonspecific endocytosis, facilitates the uptake of soluble compounds including proteins. Interestingly, although macropinocytosis is ligand-independent, it can be induced via nutrient sensing pathways in the placenta. mTORC1 inhibition due to amino acid shortage triggers macropinocytosis to maintain nutrient supply by degrading scavenged proteins into amino acids (142).

Other hydrophobic molecules such as glucocorticoid hormones can pass through the microvillous plasma membrane and so also move from maternal blood to the fetal compartment by simple diffusion down a concentration gradient. In fact, early in sheep gestation as much as 80% of circulating fetal glucocorticoid is maternally derived (143, 144). However, the extent of feto-placental exposure to the higher maternal glucocorticoid concentrations is limited by the presence of 11β-hydroxysteroid dehydrogenase type 2 (11βHSD2) in the trophoblast, which increases as gestation progresses. This metabolizes active glucocorticoids to their inactive keto forms.

#### 3.3.2 Oxygen transfer

One of the key roles of the placenta is in transporting oxygen to the developing fetus and this has been the subject of much study. Readers are referred to Saini et al for a fuller description (145).

Following implantation of the human embryo, the trophectoderm differentiates into the syncytiotrophoblast which erodes into the superficial endometrial capillary network. This had previously been interpretated as the developing embryo gaining direct access to the maternal circulation. However, following direct visualization and ultrasound guided measurement of the local pO_2_, it is now accepted that there is little, if any, maternal arterial inflow into the inter villous space (146, 147). A consequence of the lack of maternal arterial blood is that the local oxygen concentration is low ˜18 mmHg, or approximately 2.5% O_2_, prior to 10 weeks of pregnancy, and this rises approximately 3-fold after ˜12 weeks (147).

This observation had led many authors to state that the placenta develops in a “hypoxic environment”. However, this is incorrect as it is not possible to state that a tissue is hypoxic solely based on the partial pressure of oxygen. It is necessary to consider the metabolic requirement of the cells or tissue and that the local pO_2_ varies along the length of blood vessels and with increasing distance from the capillary (148). Hence, there is no universal definition or a value of pO_2_ that can be used as a definition of hypoxia (149). Furthermore, the local pO_2_ in different tissue varies enormously. For example, the pO_2_ in arterial blood is ˜100mmHg but in superficial skin it is only 8mmHg (150). In the specific context of the first trimester human placenta, this low oxygen environment is normal, and indeed is necessary for development (151). The first trimester placental tissues are not metabolically stressed, the ADP:ATP ratio is no different from that in later gestation and hypoxia inducible factor-1α is not activated (152). Hence it is appropriate to say that the first trimester human placenta develops in low oxygen environment but it is incorrect to describe this as “hypoxic”.

Placental oxygen transfer to the fetus is a three-step process: i) oxygen is supplied by the maternal uterine circulation, ii) it is transferred across the placental barrier and, iii) it is delivered to the fetus via the umbilical vein. Each of these are potential points of regulation or perturbation which can affect fetal oxygenation.

#### 3.3.3 Flow and oxygenation

Uterine arterial and umbilical blood flow are best characterized in the chronically catheterized sheep. However, ultrasound and more recently magnetic resonance imagining (MRI) based methods have allowed flow and oxygenation to be estimated in humans (153, 154). These developments facilitate investigation of the first and third steps above. Oxygen is transferred by passive diffusion across the placental barrier down a concentration gradient and is strongly influenced by permeability of the barrier. The thickness and hence the permeability vary greatly over the surface of the placenta. The vasculosyncytial membrane described in Section 3.2 is specialized for diffusive transport and this is where most transfer occurs.

The three-step process described above fits well with the 3 possible causes of fetal hypoxia (155, 156): pre-placental hypoxia, reflects a problem on the supply side and would be caused by maternal anaemia or by hypobaric hypoxia (living at high altitude). Uteroplacental hypoxia refers to a defect in the placental transfer, and post-placental hypoxia to a deficiency in oxygen extraction. These differences have been reported to be associated with differences in villus architecture (155, 157, 158). Finally, when considering placental oxygen transfer it is important to remember that the placenta consumes a considerable amount of the oxygen it receives. The endocrine function of the placenta (which requires extensive protein translation and steroid synthesis) consume ATP as does maintaining the ionic gradients that drive facilitated transport. It has been estimated, primarily from ovine data, that cation pumping and protein synthesis consume >50% of placental oxygen (159).

In humans it is generally not possible to directly measure the local oxygen concentration in the intervillous space in the placenta. Therefore, most conclusions are inferences based on histological or other features. The use of molecular markers to determine the presence and extent of hypoxia are insufficiently specific to provide a correlate of the observed histological or ultrasound differences.

#### 3.3.4 Barrier function

The placenta serves as a protective barrier for the fetus against pathogens and xenobiotics. The fused syncytium lacks extracellular space which restricts the passage of pathogens. Additionally, the syncytiotrophoblast releases antimicrobial and antiviral factors such as cytokines, peptides and extracellular vesicles containing antiviral miRNAs (160), providing further defense against pathogens. The presence of Hofbauer cells within the placental stroma (Figure 2G) (28) also suggests an additional layer of protection against pathogens which have invaded past the syncytium. However, the placenta is by no means an impenetrable barrier against pathogens. This is evident by the fact around 15% of all stillbirths in developed nations are due to infections (161, 162).

Xenobiotics, which are chemical substances foreign to a biological system such as drugs and environmental pollutants, can also pose a risk to the developing fetus. To mitigate this risk, placenta produces various xenobiotic detoxifying enzymes (163) and ATP-binding cassette (ABC) transporters which efflux xenobiotics from the placenta to the maternal circulation (164). Xenobiotic or drug metabolizing enzymes transform biologically active compounds into inert molecules that can be easily excreted. However, the placenta’s detoxifying metabolic capacity is modest, making it only a partial barrier against xenobiotics (163). ABC transporters likely make a greater contribution than metabolizing enzymes as a placental barrier to xenobiotics. These transporters, localized in the syncytium, efflux xenobiotics which have crossed the placental barrier thus limiting their transfer to the fetus.

#### 3.3.5 Endocrine function

Pregnancy places a considerable physiological load on the pregnant female and requires considerable maternal physiological adaptation. Trophoblast cells and the placenta play a critical role in these adaptations and hence the placenta should be considered a major endocrine organ (165).

At the very beginning of pregnancy, the newly fertilized zygote signals its presence to prevent the progression of the menstrual or estrus cycle. This is accomplished by the release of hormones (either steroid or polypeptide) from the trophoblast. For example, in primates, chorionic gonadotrophin is released by the blastocyst and this signals directly to the corpus luteum, to maintain progesterone production. In the sheep, interferon tau is released by the trophectoderm but this acts on the uterine epithelial cells, preventing expression of the oxytocin receptor and the estrogen receptor alpha. This blocks the oxytocin-dependent pulsatile release of luteolytic PGF2A (166). Finally, in the pig the expanding trophoblast produces estradiol which was until recently thought to be the factor responsible for maternal recognition of pregnancy. More recent work shows that the sequential action of progesterone, estradiol, IL1B2, prostaglandin E2 and interferon gamma are required (167).

Similar diversity is also found in other aspects of the endocrine function of the placenta (168). Many of the highly expressed protein hormones are present in multi-copy families that arose by gene duplication. For example, in human there is a cluster of genes derived from growth hormone on chromosome 17 but these proteins have a higher affinity for the prolactin receptor than the growth hormone receptor (169). However, in the rodent, there is a cluster of prolactin-like genes which are expressed in specific trophoblast lineages (170) and arose from the duplication of the prolactin gene (171, 172). There is similar diversity among the pregnancy specific glycoproteins (PSGs) which are part of the carcinoembryonic gene family. The distribution and structure of these genes varies considerably between species. For example, in humans there are 10 genes (and one pseudogene), but PSG genes are only found in one sub-order of rodents (173).

The site of hormone production also differs between species. In the human, the syncytiotrophoblast is the site of both nutrient and gas exchange as well as hormone production. The high metabolic activity required for these functions makes these cells susceptible to endoplasmic reticulum stress which is a feature of preeclampsia and FGR (174). In contrast to humans, in the mouse the transport and endocrine functions are found in separate regions of the placenta - the labyrinthine and the junctional zones respectively.

Maternal recognition of pregnancy occurs very early in pregnancy, but gestation as a whole is characterized by profound physiological maternal changes (165, 175, 176). Many of the changes centre on energy balance - specifically the accumulation of energy stores early in pregnancy and their subsequent release. For example, early in human pregnancy maternal appetite is increased and leptin resistance develops to allow for fat stores to be enhanced (177, 178). Later in pregnancy lipid and triglycerides are mobilized to provide additional energy for the growing fetus and lactation (179).

The supply of glucose to the fetus is central and as pregnancy progresses maternal insulin resistance rises leading to elevated circulating glucose, hence there is sufficient glucose available for fetal growth. There is a concomitant rise in circulating insulin to prevent maternal hyperglycemia and the rise in insulin demand is met by increased maternal β-cell mass. Prolactin and placental lactogen regulate this expansion but this is, in part, mediated by the local synthesis of serotonin in the pancreatic islets (180, 181). The maternal and fetal metabolic states and other placental peptides regulate β-cell mass and these have been reviewed (182, 183).

Due to the abundance of the protein hormones produced (e.g. ˜1g/day for human placental lactogen at term (184)) and the long-established importance of steroidogenesis by the placenta (185) the importance of the endocrine functions of the placenta are well recognized. However, the precise action of some factors is poorly understood and some, such as the PSGs, have no well-established function. However, new genetic and metabolic tools are allowing the identification of specific placental factors that mediate the nuanced dialogue between the feto-placental unit and the mother.

An example is the demonstration that the imprinted Igf2 gene is necessary for the adaptation of maternal glucose and lipid handling (186).

Two placental proteins of note which are implicated in adverse pregnancy outcomes are placenta growth factor (PGF or PlGF) and Pappalysin 1 (PAPPA). PlGF is a member of the VEGF family and binds to FLT1 (VEGFR1). It is produced by villous and extravillous trophoblast (187) and promotes endothelial migration and proliferation and inhibits endothelial apoptosis (188). These actions are inhibited by a soluble form of this receptor (sFLT1). PAPPA is a secreted metalloproteinase which cleaves insulin-like growth factor binding proteins (IGFBPs). This releases IGFs, allowing them to bind to and activate IGF receptors (189). The actions and use of these proteins as predictive biomarkers is discussed in sections 4.5 and 5.

#### 3.3.6 Steroid biosynthesis

The placenta is a key site for steroid synthesis during pregnancy with progesterone production being absolutely required. There are however differences in the timing of this requirement, for example progesterone production by the corpus luteum is maintained in the mouse for the whole of pregnancy although there is placental progesterone production. However, in the human, progesterone production by the placenta is an absolute requirement from approximately 8 weeks of pregnancy. Steroidogenesis and metabolism by the placenta have been well studied (190–192) and the placenta has some distinctive features, outlined below.

Sex steroids are all produced from cholesterol and the term human placenta produces ˜400mg of these hormones a day. Cholesterol is also required for fetal growth and the mechanism and regulation of placental cholesterol transport and metabolism is reviewed elsewhere (193, 194). The first, and rate-limiting step in the synthesis of all steroid hormones is the conversion of cholesterol to pregnenolone by P450scc (P450 side-chain cleavage, gene name CYP11A1). This takes place on the inner membrane of the mitochondrion (191). In most steroidogenic tissues the hormonally regulated steroidogenic acute regulatory protein (StAR) regulates the transport of cholesterol from the outer to the inner mitochondrial membrane, however, this is not the case in the placenta (190). Another notable difference in the placental steroidogenesis is that steroidogenic factor 1 (SF-1, encoded by the gene NR5A1), which regulates transcription of StAR in other steroidogenic tissues is absent from the placenta. In its place are one activating transcription factor (LBP-1b, officially termed TFCP2A) and two repressive factors (LBP-9 and LBP32, TFCP2L1 and TFCP2L2) (190, 195, 196).

#### 3.3.7 Steroid metabolism

Lipid soluble factors such as steroid hormones might be expected to pass relatively freely between maternal and fetal circulations but the placenta has specific mechanisms to prevent the passage of active maternal steroids into the fetal circulation while at the same time allowing production and release of hormones into the maternal circulation. The key to this is the location of specific enzymes that inactivate maternal hormones that could perturb fetal development.

17β-HSD1 (HSD17B1 on human chromosome 17) predominantly catalyzes the reduction of estrone to the more active estradiol and is located in the syncytiotrophoblast. By contrast, 17β-HSD2 (HSD17B2 on human chromosome 16) is located in the placental endothelial cells and it oxidizes sex steroids and hence inactivates them, transforming estradiol to estrone, testosterone to androstenedione, and DHT to 5alpha-androstanedione. Knockout of *Hsd17b2* in mice leads to 70% embryonic lethality and reduced fetal and placental size in the surviving implantations (197).

A similar system applies with 11-βHSD1 and 11-βHSD2 which metabolize glucocorticoids with the reductase (11-βHSD1) reducing cortisone to the active cortisol and 11-βHSD2, oxidizing cortisol to the inactive cortisone. Thus, for both 11-βHSDs and 17-βHSDs the type 2 enzyme (the oxidase) inactivates the steroid. In the case of 11-βHSD2 the barrier is in the syncytiotrophoblast. Protection of the fetus from the effects of excessive cortisol is important as glucocorticoids can have profound physiological effects. Ablation of 11-βHSD2 leads to a reduction of placental weight and by E18 a smaller fetus; these changes are mediated, at least in part, by altered placental nutrient transport (198). Environmental stimuli such as hypoxia or undernutrition regulate placental 11-βHSD2 activity and thus alter fetal exposure to glucocorticoids (144, 199). It is particularly important to note that some synthetic glucocorticoids (e.g. betamethasone and dexamethasone) are poorly metabolized by 11-βHSD2 and therefore the barrier is incomplete. This has clinical implications due to the widespread use of antenatal synthetic glucocorticoids to promote lung maturation and improve respiratory function. While this is very beneficial in the short term, the long-term developmental consequences are unknown (200, 201).

## 4 Clinical methods for assessing human placental dysfunction

### 4.1 Doppler flow velocimetry

**Figure 5:**
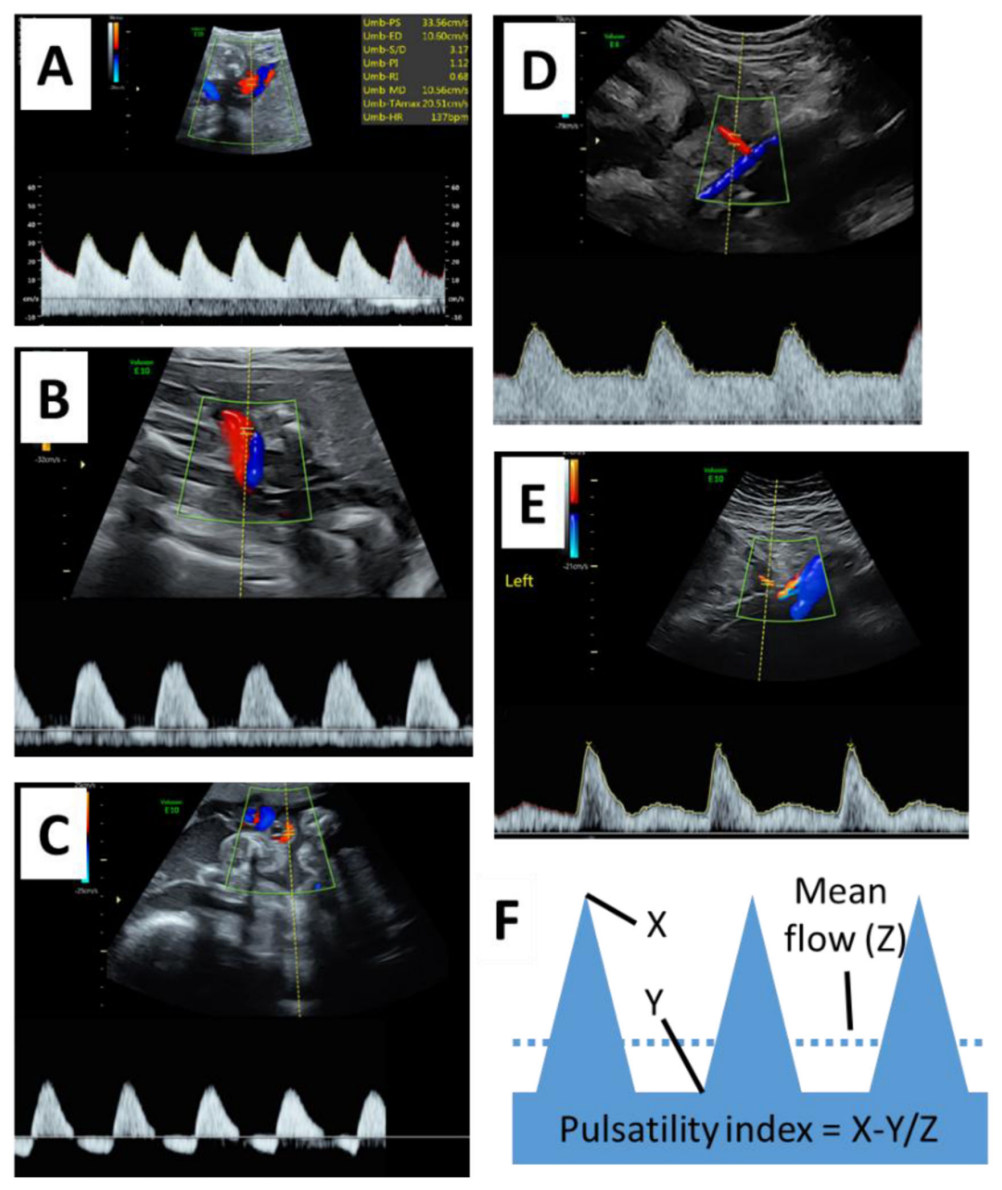
**A**. Low resistance flow in the umbilical artery, as evidenced by a high rate of forward flow at the end of diastole **B**. High resistance flow in the umbilical artery as evidenced by the absence of forward flow at the end of diastole, **C**. Very high resistance flow in the umbilical artery, as evidenced by the reversal of the direction of flow at end of diastole, **D**. Low resistance flow in the maternal uterine artery, as evidenced by a high rate of flow at the end of diastole and the absence of a notch after the initial systolic peak, **E**. High resistance flow in the maternal uterine artery, as evidenced by a low rate of flow at the end of diastole and the presence of a notch after the initial systolic peak, **F**. Calculation of the pulsatility index, a measure of downstream vascular resistance, from the Doppler flow velocity waveform.

Doppler methods are key to assessment of utero-placental insufficiency and fetal well-being. Vessels are insonated and the change in frequency of the reflected sound from the movement of blood is used to calculate the speed of blood flow. The Doppler flow velocity waveform has time as the X axis and the speed of flow on the Y axis. Qualitative assessment of the waveform is performed on the basis of the presence or absence of notches (uterine artery) or the presence, absence or reversal of flow at the end of diastole (umbilical artery) (Figure 5). Quantitative assessment of the waveform is focused on the maximum velocity of flow (most commonly in the middle cerebral artery, where it is a non-invasive indicator of fetal anemia) or indices of the shape of the waveform which are thought to provide information on the resistance to flow downstream of the point of measurement (Figure 5). Assessment of a high-risk pregnancy will involve Doppler interrogation of fetal blood vessels, principally, the middle cerebral artery and the ductus venosus, and assessment of fetal status provides indirect evidence of placental function. Low resistance patterns of fetal middle cerebral artery flow are thought to reflect fetal hypoxia, as chemoreceptor activation in the fetus results in reduced cerebrovascular resistance (202). High resistance patterns of ductus venosus flow are associated with an increased risk of fetal death (203). In chronically instrumented sheep, high resistance patterns are associated with increased systemic venous pressure and increased end diastolic pressure in the heart and can be induced by fetal hypoxia (204). The two key Doppler methods directly assessing the placenta interrogate the maternal and fetal utero-placental circulation and are discussed below.

### 4.2 Uterine artery Doppler flow velocimetry

High resistance patterns of uterine artery blood flow in the middle of pregnancy are thought to reflect impaired invasion of maternal uterine resistance vessels by the invading extravillous trophoblast. There is direct evidence from a transgenic rat model that deeper trophoblast invasion of the spiral arteries is associated with a lower resistance pattern of flow in the uterine arteries (205). Doppler flow velocimetry of the uterine arteries in women can be performed in the first trimester and this was used as one component of a combined algorithm to predict preterm preeclampsia risk in the ASPRE trial. This trial demonstrated that low dose (150mg at night) aspirin reduced the risk of preterm delivery due to preeclampsia among women who had screened high risk for the condition (206). A meta-analysis of 18 studies including ˜56,000 women demonstrated that first trimester uterine artery Doppler was potentially clinically useful in the prediction of preterm preeclampsia and FGR (207) and the diagnostic odds ratios (i.e. the ratio of the positive and negative likelihood ratios, a useful summary measure of association) for preterm preeclampsia and FGR associated with abnormal Doppler were ˜10.

Trophoblast invasion occurs as a continuous process well into the second trimester (208) and there is a sharp decline in indices of resistance in uterine artery Doppler in the second trimester (209). Based on the assumptions that high resistance uterine Doppler reflects impairment of trophoblast invasion and that trophoblast invasion continues into the second trimester, uterine artery Doppler flow velocimetry is more commonly performed around 20-23 weeks and this has generally been considered the optimal time of assessment. Consistent with this, published associations indicate diagnostic odds ratios of ˜40 for preterm preeclampsia (210) and ˜30 for preterm stillbirth (211). In the UK, national guidelines mandate assessment of uterine artery Doppler at 20-23 weeks in women with risk factors for FGR as a basis for selecting women who need serial ultrasonography during the extreme preterm period (212, 213).

### 4.3 Umbilical artery Doppler flow velocimetry

High resistance patterns of flow in the umbilical arteries are thought to reflect maldevelopment of the placental vascular tree. The villous tree of the mature human placenta is comprised of a hierarchy of three villous types: stem villi, intermediate villi and terminal villi (214). The stem villi contribute to the structural support of the villous tree, the terminal villi provide the primary site where exchange between the maternal and fetal circulation takes place, and the intermediate villi connect the two. Ultrastructural analysis reveals that high resistance patterns of umbilical artery Doppler are associated with dysfunction in the smaller terminal villi, as well as features which would reduce the diffusion of oxygen from the intervillous space into the fetal circulation (157). Stereological studies indicate that FGR is typically associated with a mean reduction in fetal capillaries of 40% (214). Studies in a chronically instrumented fetal sheep have demonstrated that high resistance patterns of flow in umbilical artery Doppler were associated with increased vascular resistance in the placenta and reduced placental blood flow (215, 216). Moreover, embolization of the fetal side of the placental circulation increased placental vascular resistance and this was strongly correlated with increased umbilical artery Doppler flow resistance indices (217).

Clinically, umbilical artery Doppler flow velocimetry is one of the most important measurements in assessing fetal well-being. The mildest abnormality of flow is where there is positive end diastolic flow but measures of resistance are increased above the normal range. Serious abnormalities of flow are characterized qualitatively on the basis of the absence or reversal of direction of flow at the end of diastole (Figure 4). Where the fetus is small for gestational age, medically indicated delivery is recommended at 34wks gestation where end diastolic flow is absent and 32wks gestation where it is reversed (212).

### 4.4 Other ultrasonic methods of placental assessment

One of the most important roles for ultrasound in the assessment of the placenta outside the context of utero-placental insufficiency is in the diagnosis of abnormally invasive placenta (AIP, this range of conditions has other synonyms including “placenta accreta spectrum” or “placental adhesive disorders”). AIP is a potentially life-threatening complication for the mother where trophoblast invasion extends well beyond its normal physiological limits, sometimes penetrating into adjacent sites (bladder and parametrium), which is called placenta percreta. Ultrasonic assessment, which involves both 2D imaging and Doppler interrogation to determine the presence of high flow “tornado” vessels in the placental parenchyma has 80-90% sensitivity and ≥95% specificity for different manifestations of the spectrum of AIP (218).

Assessment of placental size using 2D imaging has reported a number of associations with outcomes but it is not routinely performed (219). 3D ultrasound can be used to assess placental volume in the first trimester of pregnancy although it is only weakly predictive of adverse pregnancy outcomes and not used clinically (220). Application of the method is limited but may be facilitated by automated capture of placental volume using artificial intelligence (AI) methods (221). In the first trimester of pregnancy, the combination of 3D scanning and power Doppler allows the vascular supply to the placenta to be assessed and a review of studies indicates that placental vascular indices were significantly lower in women who went on to develop preeclampsia (222). However, standardizing the quantification of placental vascularity remains problematic, for example, in relation to different degrees of maternal obesity. Similarly, placental elastography is a novel indicator of placental function which involves assessing tissue deformation in response to some form of external stimulus (e.g. pressure or an external acoustic source) and calculates measures of placental stiffness. Similar to 3D ultrasound methods discussed above, while disease associated changes have been observed with placental elastography, there are challenges in standardization of measurements and these methods are not part of routine care (223).

Abnormal cord insertion can be reliably diagnosed by ultrasound combined with color Doppler, with >99% sensitivity and specificity (224). This may be clinically important to know because a meta-analysis of four studies relating antenatal diagnosis demonstrated that a velamentous cord insertion was associated with a 248g (95% CI 144 to 352) lower birth weight (225). It has been hypothesized that velamentous cord insertion reflects excessive chorionic regression in the first trimester and is associated with failure of conversion of the spiral arteries (226). However, the clinical utility of abnormal cord insertion is limited to diagnosis of vasa previa, i.e. where the umbilical cord has a lateral insertion into a low-lying placenta and where the area of membrane traversed by the umbilical blood vessels overlies the cervix (227). Diagnosis of vasa previa mandates caesarean delivery, which is life saving for the fetus (because allowing labor to happen might risk the presenting part of the fetus shearing open these fetal vessels as it descends down the birth canal resulting in fetal exsanguination).

### 4.5 Magnetic resonance imaging (MRI)

The main application for MRI in clinical assessment of the placenta is in the management of AIP. Suspected cases are assessed both by ultrasound and MRI with the latter being particularly useful in delineating the extent of invasion, particularly in cases where visualization by ultrasound is problematic, such as maternal obesity or a posterior placenta (228). Whereas MRI has a key role in the management of AIP it has not yet become a routine part of assessment of functional abnormalities of the placenta in the context of complications such as FGR and preeclampsia, although there are potential avenues for future applications.

In its simplest form, MRI can provide the same structural information about the placenta as ultrasound, such as placental dimensions and volume. Developments of MRI for applications outside of pregnancy has generated tools which could be used for functional assessment of the placenta, including assessment of perfusion rate (229), fractional blood volume (230), permeability surface area (231), perfusion fraction (232) diffusional properties (233), oxygen levels (234) and estimation of metabolite levels (235). However, some of these measures utilize dynamic contrast-enhanced (DCE) MRI which requires the use of intravenous contrast agents. Unfortunately, the most commonly employed, gadolinium, is known to cross the placenta in non-human primates (236, 237). Long term follow-up of about 400 infants exposed to gadolinium MRI *in utero* demonstrated increased risks of connective tissue disease, stillbirth and neonatal death (238). Hence, the use of gadolinium is limited to a very small number of cases where the benefits outweigh these risks.

Other MRI methods that do not use contrast agents (arterial spin labelling, blood oxygen-dependent (BOLD-MRI) and diffusion weighted MRI) have a range of other limitations which make patient-to-patient comparison of values problematic. Estimation of metabolites using MRI spectroscopy also has practical limitations. Hence, functional MRI has no routine clinical role in the investigation and management of the placenta in FGR or preeclampsia. However, it is an avid area of study and it is conceivable that it may have utility in the future, particularly as newer contrast agents for DCE are available which do not cross the placenta (231).

### 4.6 Biomarkers

There are a number of maternal serum biomarkers currently used clinically to assess the risk of placental complications. The use of these tests varies between and within countries, however, and there is currently no biochemical placental function tests used consistently throughout the world which are comparable to the universally performed renal or liver function blood tests.

Placental production of the soluble form of the fms-like tyrosine kinase receptor-1 (sFLT1) was first described in 1998 (239). In 2003, a model for preeclampsia was proposed whereby sFLT1 was released from the placenta in response to malperfusion ischemia and that sFLT1 bound and inactivated growth factors including placenta growth factor (PlGF) and vascular endothelial growth factor A (VEGF-A) (240). As these growth factors are critical for maintaining the endothelium in a healthy state the model provided a link between placental malperfusion ischemia and the endothelial dysfunction which characterizes preeclampsia. Analysis of stored samples from cohort studies subsequently demonstrated that changes in maternal serum levels of sFLT1 and PlGF preceded the clinical onset of preeclampsia by many weeks (241). Prospective studies of diagnostic test accuracy subsequently confirmed that the sFLT1:PlGF ratio had clinically useful prediction to rule in and rule out preeclampsia among women in whom the disease was clinically suspected (242). A number of clinical assay platforms were developed, some using both sFLT1:PlGF and some just PlGF. A stepped wedge randomized controlled trial demonstrated measuring PlGF in the context of preterm preeclampsia expedites diagnosis and reduces the risk of severe maternal morbidity (243). Testing using either sFLT1:PlGF or PlGF on its own is now routine practice in the UK and much of the rest of Europe, although it is not currently in widespread use in the USA. However, an sFLT1:PlGF assay platform was approved by the FDA in 2023 (244).

sFLT1 and PlGF have also been evaluated as predictors of disease in early pregnancy. A complicating factor is that the relationship between sFLT1 and adverse outcome is in the opposite direction in the first trimester, when higher levels of sFLT1 are associated with a reduced risk of complications (245, 246). However, low PlGF is consistently associated with an increased risk of complications across the whole of gestation and this is incorporated into first trimester assessment of the risk of preeclampsia and is used to target aspirin prophylaxis to the women who are most likely to benefit (206). Another first trimester biomarker, low PAPP-A, was found to be predictive of a range of adverse pregnancy complications (see below). PAPP-A was identified as a marker through secondary analysis of data collected to assess its predictive utility in screening for Down’s syndrome. PAPP-A is a protease for insulin-like growth factor binding proteins (IGFBP) 4 and 5 (247). Lower levels of PAPP-A would, therefore, be expected to result in decreased bioavailable IGF2 which is known to be a critical fetal growth factor (248).

**Figure 6:**
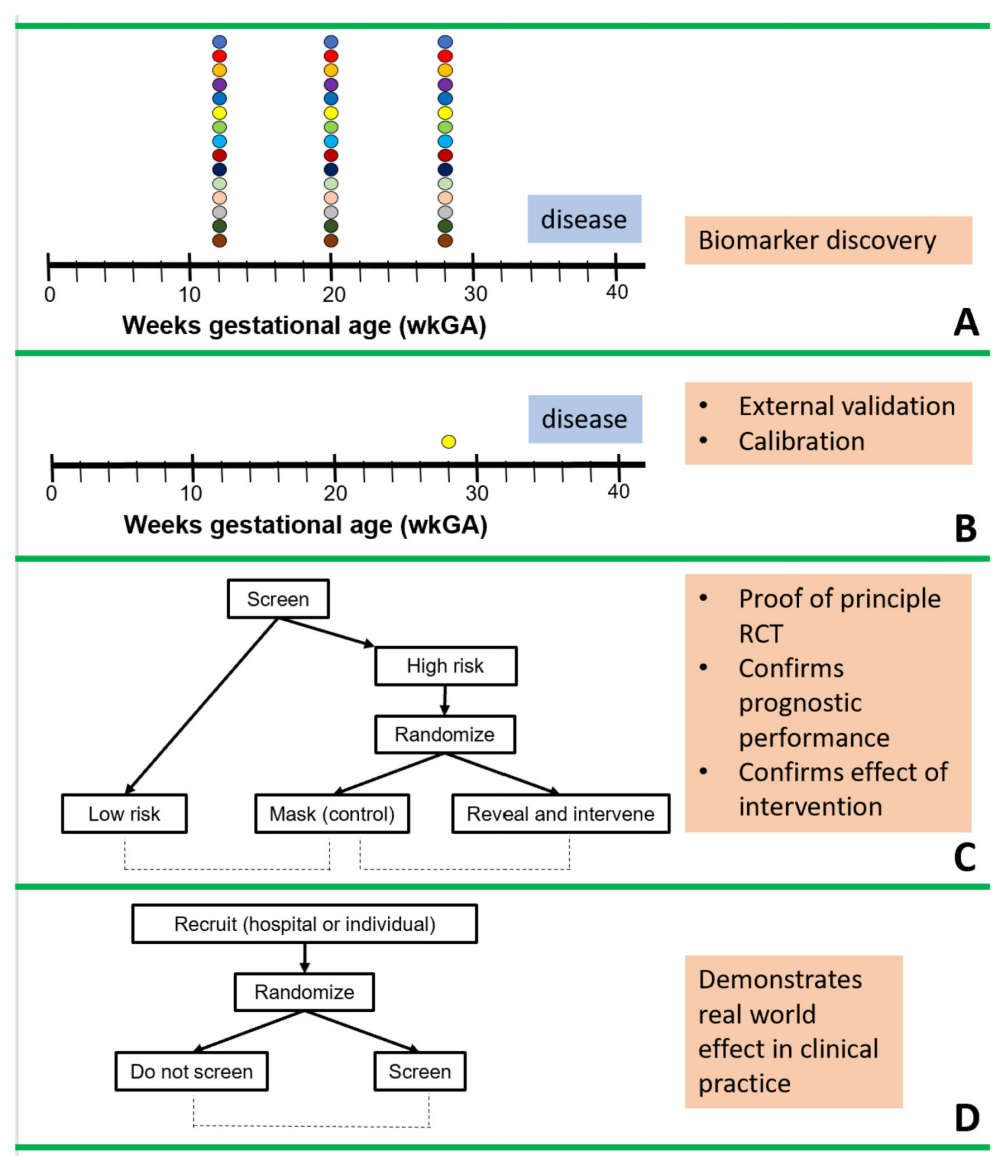
Schematic illustration of clinical study design for the implementation of a biomarker following omic discovery. **A**. In the original discovery study a candidate test is identified in a study which has a large number of hypothesis tests. **B**. Having identified the candidate, its predictive utility needs to be externally validated. This rules out the possibility that the initial findings were a false discovery due to a large number of hypothesis tests. It also assesses the test in separate and, ideally, diverse populations (confirming external validity) and the absolute risk of the outcome in relation to the level of the test (calibration). **C**. A proof of principle study randomizes high risk women to intervention or routine care, and this allows both validation of the screening test and assessment of the effect of the intervention. This study design also requires a much smaller sample size than the design below (253). **D**. Randomization to screen or no screen gives the best information about the likely effect of implementation of screening into a healthcare system. Implementation of the screening program into a group of hospitals can be both performed and assessed by randomization at the level of the hospital, using a stepped wedged cluster randomized controlled trial (254).

A wide range of other placentally derived proteins have been reported to be associated with pregnancy complications, as well as steroid hormones released by the placenta (249). More recent studies have also applied omic methods and identified other predictive molecule types including metabolites (250) and cell free RNA (251, 252). Translating findings from omic experiments through to clinical implementation requires the generation of multiple levels of clinical evidence and this is illustrated schematically in Figure 6.

## 5 Placental dysfunction in adverse pregnancy outcome

### 5.1 Hyperemesis gravidarum

Nausea and vomiting are common in early pregnancy, affecting almost 70% of pregnant women (255). Although these generally subside by 16-20 weeks, in about 35% of these women the symptoms are clinically relevant (256). However, in some women nausea and vomiting is so severe that they are unable to eat and drink normally, suffer weight loss and normal daily function is compromised, and this is described by the term hyperemesis gravidarum (HG) (257). The reported incidence of HG is 1.1% but reported rates vary widely and this probably reflects the lack of a rigorous definition although a consensus definition was recently agreed (255, 257, 258). HG is the most common reason for hospital admission in the first trimester of pregnancy and is associated with an estimated economic burden that is substantial (>$800M in 2014 in the USA, (259, 260). In addition to the considerable maternal morbidity during pregnancy, HG poses a significant risk for the longer-term health of both mother and child (261–263). Despite the prevalence and impact of HG, a review found that the certainty of evidence for the available treatments was either low or very low (264).

Given the timing of the onset and frequent resolution of symptoms, it is unsurprising that factors released by the placenta in early pregnancy have been investigated as possible causes of HG and, historically, the most prominent of these is hCG. However, a systematic review of this and other risk factors including Helicobacter Pylori infection, pre-pregnancy body mass index (BMI), adipose tissue, maternal age, leptin, ghrelin, total (T4) and free thyroxine (fT4) concluded that the supportive evidence was poor and that high quality studies with adequate sample sizes need to be carried out. The most consistent risk factor is a positive family history. Indeed, a twin study concluded that nausea and vomiting during pregnancy was highly heritable (73% (95 % CIs = 57–84 %) for the presence of HG) (265).

Recent studies have demonstrated that the likely mechanism of HG arises from the placenta, specifically the >100-fold increase in maternal circulating levels of GDF15 (266). This is an agonist of the GFRAL receptor which is located solely in the hindbrain and causes nausea and vomiting. The initial finding implicating GDF15 was a genome wide association study which leveraged the considerable information available from 23andMe customers (a personal genomics company offering genotyping to the public). This study identified 1306 HG cases - participants who reported receiving intravenous fluid therapy for nausea and vomiting in pregnancy (NVP) whereas the controls (15,756) were women who reported no NVP (267). There were two loci that reached genome-wide signiﬁcance (p < 5 × 10^−8^), chr19p13.11 (rs45543339, OR = 0.67, 95% CI [0.60, 0.74], p = 1.9 × 10^−14^) and chr4q12 (rs143409503, OR = 0.75 [0.69, 0.82], p = 4.5 × 10^−10^). The most plausible genes at, or close to these lead SNPs were GFD15 and IGFBP7 respectively. This study also included a replication cohort of 53,731 unrelated female 23andMe customers in which NVP was graded on an ordinal scale (none, slight, moderate, severe and very severe). This confirmed the initial observation with even smaller p values (2.4 × 10^−41^ 9.2 × 10^−24^ respectively). Moreover, a SNP upstream of the gene encoding a subunit of the GDF15 receptor (GFRAL) reached genome-wide significance in the original study (6.2 × 10^−9^) adding further weight to the likely involvement of this pathway in HG. Further genetic support for a role of GDF15 was reported in a whole exome sequencing study in which rs1058587 in GDF15 easily met exome-wide significance 9.98 × 10^−11^ (268). Experimental support for a role of GDF15 includes the observation that non-human primates treated with platinum-based chemotherapy exhibit elevated circulating GDF15 and the vomiting that ensues can be ameliorated by neutralizing GDF15 (269, 270). Notably, none of the genome-wide association or exome-wide sequencing studies described above, showed any genetic association with either of the hCG sub-unit genes.

Recent data have now confirmed that circulating GDF15 is raised in the first trimester of pregnancy in women with HG compared to those not experiencing nausea and vomiting (271). However, the levels of GDF15 in maternal blood are not the sole determinant of the risk of HG as maternal sensitivity to the effects of GDF15 also plays an important role. Intriguingly, the extent of pre-pregnancy exposure to GDF15 appeared to determine the response in pregnancy. Exposure to low pre-pregnancy levels of GDF15 caused by a missense mutation in the mother, increased sensitivity to feto-placental GDF15 produced in pregnancy. Conversely chronic high levels of GDF15 before pregnancy, as found in patients with thalassemia, was associated with a lower prevalence of nausea and vomiting (271). Furthermore, experimental evidence in mice demonstrated directly that the response to GDF15 is blunted by previous exposures to high levels of the hormone.

Studies of genetic variation in GDF15 in the maternal-fetal dyad have demonstrated that the vast majority of GDF15 in maternal blood in pregnancy originates from the placenta and/or the fetus. The presence of a common coding genetic variant (rs1058587) which alters amino acid residue 202 of GDF15 allowed the allele-specific detection of a GDF15 derived peptide containing either the histidine or aspartic acid residue (H or D) at this position. In pregnancies where the fetus was homozygous for the H encoding allele and the mother was heterozygous (ie HD), less than 1% of the GFD15 circulating in the mother’s blood contained the aspartic acid (D) containing peptide (271). Early reports describing the sites of synthesis of GDF15 (initially known as Macrophage Inhibitory Cytokine-1, MIC-1) showed dominant immunohistochemical staining of the trophoblast but also some staining in the decidual stromal cells (272, 273). However, more recent immunostaining data (in the Human Protein Atlas for example (274)) only reports very strong trophoblast staining in the placenta and a primarily feto-placental source of GSF15 is consistent with the maternal-fetal genetic studies.

The circulating levels of GDF15 in human pregnancy rise ˜100-fold (non-pregnant vs mid-gestation)(266). These levels are considerably higher than those observed in other conditions, for example, the circulating level in pancreatic ductal adenocarcinoma is ˜2,400 pg/ml whereas in mid-gestation it is ˜25,000 pg/ml (275). The mRNA encoding GDF15 is also >100 times higher in the term human placenta than the median value of all the other tissues assessed by the GTEx consortium (790 vs 6 transcripts per million) (276). A similar rise is seen in pregnant macaques (266). However, this is in marked contrast to mice and rats where the observed rise is only ˜2-fold (266).

The regulation of GDF15 has attracted much attention, particularly as it plays a central role in appetite and weight control. Binding sites for several transcription factors have been identified and experimentally validated (p53 and EGR1, (277, 278)). These studies have been performed in the context of cancer cells – as many tumors express high levels of GDF15. However, it is generally expressed at low levels in healthy tissues other than the placenta, but rises under conditions of cellular stress or injury. This is consistent with the binding of another transcription factor (CHOP) to the GDF15 promotor (279). CHOP is a terminal effector of the integrated stress response (ISR) which is an adaptive cellular response to a wide range of stressors. These include endoplasmic reticulum stress, amino acid deprivation, viral infection or heme deficiency.

Identifying the role of GDF15 in HG has major clinical implications. One approach to reducing the risk of HG would be to use interventions which increase maternal circulating levels of GDF15 pre-pregnancy, such as treatment with metformin (280). Pre-pregnancy treatment with metformin could result in desensitization to GDF15 and reduced experience of nausea and vomiting in pregnancy. However, the women who would be most likely to experience HG might be expected to have increased severity of side effects with metformin. An alternative approach would be to administer a monoclonal antibody which binds GDF15. Given that the only known location of GFRAL is the hindbrain, removing GDF15 would be anticipated not to have off target effects. One potential concern is that, as discussed above, IgG can cross the placenta, mediated by the neonatal Fc receptor in the syncytiotrophoblast. The effects of removing circulating GDF15 in the fetus are unknown. Any theoretical risks would have to be considered in the light of the potentially life-threatening nature of severe HG. However, perhaps the optimal approach to treating HG would be an anti-GDF15 monoclonal antibody which lacks the Fc component of IgG and which would not, therefore, be transported across the placenta into the fetal circulation. Some existing MABs have been created which have this property, such as certolizumab, an anti TNF-alpha IgG which lacks the Fc component of IgG and is either undetectable or present at minimal concentrations in cord blood (281). Finally, as GDF15 is a marker of cellular stress and is mostly produced by the placenta, it may have utility as a biomarker of placental dysfunction.

### 5.2 Stillbirth

#### 5.2.1 The clinical problem of stillbirth

Stillbirth is defined as delivery of an infant showing no signs of life. Arbitrary gestational age thresholds for the timing of intra-uterine fetal death (IUFD) are used to differentiate between pre-viable losses and stillbirths, being 24 weeks of gestation in the UK, 20 weeks of gestation in the USA, and 28 weeks of gestation by the World Health Organization definition (282). As with death of a child or adult, death of the fetus can be the end point of diverse pathophysiological processes. However, unlike most deaths of children and adults, understanding the precise cause of stillbirth is possible in only a minority of losses. The proportion of stillbirths which are unexplained varies in different studies and is typically between 30% and 60% (282). However, much of the variation relates to the preparedness of the classifier to accept associated findings which are only possible or probable factors as the “cause”.

The placenta can be a clear, direct cause of stillbirth and examples are placental abruption (i.e. whole or partial separation of the placenta from the uterus prior to expulsion of the fetus) or feto-maternal hemorrhage (i.e. exsanguination of the fetus into the maternal circulation). More commonly, the placenta is implicated in the possible etiology of IUFD by the presence of a co-existing complication which is itself thought to be related to placental dysfunction and examples are FGR and preeclampsia. Finally, the placenta may be implicated in the etiology of a stillbirth through histopathological examination, which is mandated in clinical guidelines of stillbirth management. A complicating feature is that abnormal placental histopathology findings are relatively common in pregnancies with a completely normal outcome (283). While research reveals an excess of some of these findings in cases of stillbirth (see below), an uncritical pathologist may be prone to reporting all abnormalities as causative when, frequently, the inference of causality is not robust.

#### 5.2.2 Macroscopic and microscopic placental findings in stillbirth

Pathological abnormalities can be broadly classified into developmental, inflammatory and circulatory. Developmental abnormalities can be macroscopic, such as abnormal umbilical cord insertion into the periphery of the placenta, or microscopic, such as a thicker barrier to diffusion near term, called villous immaturity. Inflammatory disorders can be acute or chronic and can involve the umbilical cord, fetal membranes, the placental villi and the inter-villous space. Circulatory disorders can be macroscopic, such as infarctions and thrombosis, or microscopic indicating malperfusion of the maternal or the fetal vascular sides of the placenta. However, a major barrier in the field has been variation in sampling procedures, nomenclature and definitions which can make comparison of different studies problematic. An international consensus statement on all aspects of the approach to placental pathology was published in 2016 (284) and widespread adoption would help harmonize future research.

Systematic analyses of placental pathology using rigorous classification systems indicate that placental disorders are the biggest single determinant of stillbirth but the proportions estimated from large scale high quality studies vary from about 25% in a US analysis (285) to 65% in a Dutch analysis (286). Both studies demonstrated that the placental contribution to stillbirth was smallest at 20-23 weeks, these losses being commonly due to cervical incompetence and/or infection. The Dutch study estimated that about 80% of term stillbirths were due to placental dysfunction with villous immaturity, hypoplasia and infarction accounting for about half of all term stillbirths. The US study compared the frequency of placental findings in term stillbirths and controls and also found associations with villous immaturity and infarction, as well as a number of other features (including single umbilical artery, vascular thrombi in the chorionic plate, avascular villi and inflammatory changes) (287).

FGR is a risk factor for stillbirth, is frequently presumed to be due to chronic placental insufficiency (i.e. where placental nutrient and gas exchange are inadequate to meet the genetically determined growth potential of the fetus) and is itself associated with a range of placental pathological findings (288). However, the inter-relationships between FGR, placental pathology and stillbirth are complex (289, 290). FGR can occur in cases where placental histology appears normal. Conversely, features of abnormal placental histopathology are frequently seen in pregnancies with a normal outcome. Moreover, there are multiple histopathological findings which are a consequence of IUFD rather than a cause, i.e. the placenta may exhibit abnormalities which were unrelated to placental dysfunction and where the changes developed post-mortem (291).

Abnormal utero-placental Doppler tends to be a better predictor of stillbirths occurring preterm (see below). However, as discussed above, a large proportion of late stillbirths exhibit placental histopathological abnormalities hence it is clear that stillbirth can be the end point of multiple different manifestations of placental dysfunction. A re-analysis of the Stillbirth Collaborative Research Network case control study confirmed that a number of pathological features of the placenta were associated with stillbirth but the association was not mediated by whether the fetus was small for gestational age (weight <10^th^ centile) (292). Placental features associated with stillbirth are tabulated by whether they are also associated with fetal growth disorder (Table 1).

#### 5.2.3 Maternal serum biomarkers of placental dysfunction and the risk of stillbirth

It was first demonstrated more than 20 years ago that low levels of PAPP-A in the first trimester of pregnancy were associated with an increased risk of stillbirth (293). A systematic review of maternal serum predictors of stillbirth (294) reported that multiple studies replicated the association with PAPP-A and that PAPP-A was one of two stillbirth predictors which were potentially clinically useful. Follow up of women with low early pregnancy levels of PAPP-A has been incorporated into clinical guidelines with the aim of reducing the risk of stillbirth (212, 213). Although low PAPP-A is only weakly predictive of all cause stillbirth, it is much more strongly associated with stillbirths associated with FGR, preeclampsia and/or placental abruption (295). Trying to identify a single biomarker which is predictive of all cause stillbirth may be an impossible task. Given the diverse causes of stillbirth, it is very unlikely that a single test will show a high level of clinical prediction for stillbirth as an entity as tests tend to be specific for a given pathophysiological pathway and, therefore, only be associated with a fraction of stillbirths. Consequently, tests should not necessarily be dismissed on the grounds that they are not strongly predictive of all cause stillbirth.

There are a number of other placentally-related proteins which are associated with stillbirth and these have generally been measured in the context of Down’s syndrome screening. Low maternal serum second trimester levels of estriol and high levels of AFP, hCG and inhibin are all associated with an increased risk of later stillbirth but a meta-analysis indicated that the associations were generally weak (294). Adding measurement of AFP and hCG to maternal characteristics had a minimal effect on prediction of stillbirth risk at term but had a much greater effect on the prediction of stillbirth risk at preterm gestational ages (296).

#### 5.2.4 Ultrasonic assessment of the placenta and the risk of stillbirth

Ultrasonic assessment can identify features of the placenta, membranes and umbilical cord which are causes of (e.g. vasa praevia (297)), or are associated with stillbirth (e.g. single umbilical artery (298) or velamentous cord insertion (225)). High resistance patterns of uterine artery blood flow in the middle of pregnancy are a potentially clinically useful predictor of stillbirth identified by the systematic review (294). As with PAPP-A, the associations differ in relation to the phenotype of the stillbirth, being much stronger for losses where one or more of the following was present: small for gestational age, placental abruption and/or preeclampsia. As these stillbirths tended to occur at earlier gestational ages, uterine artery Doppler was a much better predictor (area under the receiver operating characteristic curve [AUC], 95% CI) of stillbirth <33 weeks (0.84, 0.77–0.91) than stillbirth ≥33 weeks gestation (0.62, 0.54–0.70) (211). Given the strong association with preterm stillbirth, uterine artery Doppler flow velocimetry is used clinically to identify which women with risk factors for stillbirth need to be serially scanned prior to 32 weeks (212, 213).

High resistance patterns of flow in the umbilical artery are also associated with an increased risk of stillbirth in the presence of FGR (203). Assessment of umbilical artery Doppler is a key element of clinical management of pregnancies complicated by suspected FGR (see below) (299). A 1987 study indicated a substantial risk of stillbirth when there was marked calcification of the placenta noted at 34-36 weeks gestation (300). However, the finding was associated with a number of other risk factors, including nulliparity, teenage pregnancy and smoking. While there was no multivariate analysis performed, the association with perinatal death was strong (odds ratio 7.5) and such strong associations are uncommonly wholly explained by confounding. Given other evidence that premature placental senescence may be a cause of adverse pregnancy outcomes (301) there would be merit in further studies assessing the predictive utility of premature placental calcification as a predictor of stillbirth.

### 5.3 Fetal Growth Restriction

Fetal growth restriction (FGR) is a pathological condition where fetal growth falls short of its genetically predetermined potential (302). The most common cause is placental insufficiency, where the fetus does not receive enough oxygen and nutrients to sustain healthy growth and development. Historic cordocentesis studies (303) and recent MRI studies (304) provide direct *in vivo* evidence that growth restricted fetuses are hypoxic. Growth trajectory slows in FGR (302), and the fetus becomes smaller relative to healthy fetuses at the same gestation.

Most cases of FGR are clustered among fetuses with a weight under the 10^th^ centile, but some fetuses over this arbitrary cut-off may be affected by placental insufficiency and are also growth restricted (302). Conversely some below the 10^th^ centile are constitutionally small and healthy, where perfusion of the placenta and placental function is normal (302). The probability that a fetus is affected by pathological growth restriction (not just constitutionally small) rises with progressive centile declines, particularly those under the 10^th^ centile (305). Consequently, most fetuses <3^rd^ centile are likely to have true FGR (where many of the pathological features described below are present); they are at the greatest risk of perinatal morbidity and mortality (306–308).

As noted above, the most immediate risk of placental insufficiency is stillbirth. The risk rises with decreasing fetal weight centiles and is markedly higher for those less than the 3rd centile (309, 310). FGR infants are at greater risk of many complications during the neonatal period, such as neurological injury caused by low oxygenation (hypoxic ischemic encephalopathy), feeding difficulties, hypothermia, hypoglycemia and many other conditions (311).

Protracted impairment of nutrient and gas exchange in utero also gives rise to a legacy of adverse health outcomes across the life course. Risks in childhood include reduced cardiometabolic health (312, 313), and poorer neurodevelopmental (314) and childhood development outcomes (315). The adverse conditions in utero can also program the physiology of the unborn fetus in ways that increase the risk of many common adult conditions developing decades after the birth (7, 316). Low birthweight is linked with adult onset stroke (317), coronary artery disease (317–319), diabetes (320), obesity (321) and other metabolic conditions (322).

#### 5.3.1 Early pregnancy origins of fetal growth restriction

There is clinical evidence suggesting the pathology causing FGR can begin in early pregnancy. A crown-rump length in the first trimester shorter than that expected from calculating gestation age from the first day of menstruation (period dating) is associated with FGR (323). As noted, low circulating first trimester circulating concentrations of PAPP-A (a protein released from the placenta) is associated with a 2-3 fold risk of a small for gestational age infant (<10^th^ centile birthweight) (293). A low placental volume measured by ultrasound during the first trimester also increases the likelihood that the infant will be born small for gestational age (324).

#### 5.3.2 The maternal vessels in the uterus and decidua

There are many lesions seen in the maternal vasculature in association with FGR. The net effect is reduced blood supply to the placenta, which reduces nutrient and gas exchange. Many of these lesions are also seen in preeclampsia, a related placental condition.

The Amsterdam Placental Workshop Group released a consensus statement that sought to harmonize terminologies used to describe pathologic placental lesions (284). They propose two umbrella terms to group lesions. ‘Maternal vascular malperfusion’, are lesions related to altered uterine and intervillous blood flow and may compromise the efficient exchange of nutrients across the maternal-fetal interface, see Table 2). There are broadly three types, gross changes, vascular lesions in the decidua (and can be identified on decidual segments left on the placenta sent for histopathology analysis) and villous changes in the placenta.

The second umbrella term proposed by the Amsterdam Placental Workshop Group is ‘fetal vascular malperfusion’, lesions associated with fetal blood flow obstruction (Table 2). These are mainly lesions in the fetal vascular system in the placenta and are covered later.

As noted, in normal placental invasion the spiral arteries are remodeled by invading extravillous trophoblast (18, 325). The vessels are stripped of their vascular smooth muscle and surrounding elastin, leaving non-contractile flaccid conduits that open into the intervillous space (326). Modelling suggests the rate of blood flow slows 3-5 fold upon reaching the arterial mouth of the dilated segment (2-3 m/s to 10 m/s) (19). Vascular remodeling also prevents spontaneous arterial constriction and pulsatile perfusion (19). This increased capacity (to hold a large volume of blood) and slowing of flow velocity provides optimal conditions for efficient nutrient and oxygen exchange.

Histopathological examination of decidual segments on placenta post birth has revealed FGR is associated with impaired spiral artery remodeling. The vessels remain narrow and vasoactive. This causes pulsatile, turbulent blood flow into the intervillous space, resulting in mechanical injury of the delicate villous tree, as well as ischemia, hypoxia-reperfusion injury and oxidative stress responses (19, 326, 327). Luminal intimal thickening can occur (328) as well as fibrinoid deposition in the intervillous spaces, and both further decrease efficient oxygen and nutrient exchange (as they increase diffusion distance).

There is a constellation of other pathological microscopic lesions that can be observed in the vessels on decidual remnants (284, 329–331). The presence of these is thought to reflect the presence of maternal vascular malperfusion (284) (Table 2). Placental thrombosis and infarcts are commonly seen lesions in pregnancy complicated by FGR (332). Other lesions include fibrinoid necrosis (spiral artery walls are replaced by dense, brightly eosinophilic fibrinoid tissue), arterial thrombosis, mural hypertrophy (thickening of the smooth muscle wall of the spiral artery) (331) and acute atherosis (fibrinoid necrosis and lipid-filled foam cells [macrophages] in the walls of spiral arteries, perivascular lymphocytic infiltration and reduced vessel caliber) (333–335).

FGR is also associated with increased resistance to blood flow in the uterine arteries, the main source of maternal blood to the placenta. This can be seen on ultrasound as abnormal uterine artery Doppler waveforms (reduced flow velocity at the end of diastole) and likely reflects upstream vessel impedance (336). As noted above, its presence at 20 weeks gestation can predict subsequent FGR and preeclampsia, but the associations are strongest with preterm disease (219, 337). Abnormal waveforms have been correlated with histopathological evidence of impaired trophoblast migration (338). Although it has been long thought that the increased resistance occurs at the level of the inadequate remodeled spiral arteries, computer modelling has postulated that reduced dilatation of radial arteries (proximal to spiral arteries) could be the main rate limiter to blood flow that gives rise to abnormal uterine artery Doppler waveforms (336, 339).

#### 5.3.3 Gross ultrasound and anatomical changes in the placenta

FGR is associated with placental hypoplasia: the placenta is smaller and weighs less (<10^th^ centile weight) (331), and the umbilical cord is thinner (also <10^th^ centile). Ultrasound imaging and MRI studies have shown placentas in established growth restricted pregnancies can be globular rather than a flattened disc – the placenta is thicker (340). There is an increased thickness to volume ratio (341) and less surface area abuts the decidual surface which, presumably, reduces nutrient exchange. A small magnetic resonance imaging study even demonstrated an association between the thickness to volume ratio and perinatal mortality (341).

Marginal umbilical cord insertion (attached to the side of the placenta, not the middle) is modestly associated with an increased risk of FGR (225). The association with FGR may be due to less efficient maternal to placental nutrient exchange at the opposite side of cord insertion. Also, the presence of one umbilical artery (rather than two) doubles the risk of a small fetus (342).

#### 5.3.4 Rarer vascular abnormalities in the placental bed and changes in vessel blood flow

Rarely, a massive subchorial thrombosis can arise, involving at least 50% of the chorionic plate. Called a Breus’ mole – it has a very high risk of growth restriction or fetal demise (343). Placental infarctions are thought to arise from complete obstruction of uteroplacental arteries and interruption of maternal blood flow (leading to necrosis of villous tissue). Isolated infarcts can be present in normal pregnancies whereas with FGR, larger infarcts are often seen in combination with intervillous thromboses and extensive fibrin deposition. This cluster of lesions can be seen on ultrasound as complex echogenic intraplacental masses close to the basal plate (332).

Maternal floor infarction is a rare but significant pathological lesion with villous necrosis, fibrin deposition, thromboses and hematomas (332). A small case series suggests it has a very strong association with FGR and stillbirth (344). It is thought to be caused by massive fibrin deposition in the intervillous space, rather than arterial occlusion of maternal decidual vessels.

#### 5.3.5 Histological changes in the placenta

There are a multitude of changes at the microscopic and molecular level that impede nutrient and gas exchange in the growth restricted fetus. Not only is the placenta from FGR small, but what is present has reduced villi of all subtypes. The terminal villi are thinner, there is reduced density, and reduced elongation and branching (157, 345, 346). This decreases the total surface area for nutrient and gas exchange. As noted, many of these lesions are classified under the umbrella term of ‘fetal vascular malperfusion’ (see Table 2) (284).

When around 30% of the placental villous vasculature is severely damaged (such as by thrombosis), increased umbilical artery blood flow resistance can be seen on Doppler ultrasound (347). When a large amount of the placental villous vasculature is non-functional (estimated by sheep studies to be around 60 to 70% of the entire placental area) blood flow resistance may be so severe that forward flow is absent, or even reversed at the end of diastole. This is why, as noted above, that Doppler ultrasound findings correlate with prognosis and stillbirth risk and is used in the clinic to serially monitor the wellbeing of growth restricted fetuses (299).

There are villous developmental defects postulated to happen because of under-perfusion of the intervillous space caused by maternal vascular malperfusion (placental injury caused by both reduced blood flow and fluctuations in oxygen tensions inside the placenta). These lesions are classified under the term maternal vascular malperfusion (Table 2). Distal villous hypoplasia is a paucity of villi in relation to the surrounding stem villi. The villi are thin and elongated-appearing and there are increased syncytial knots (284). Villous infarction may occur as well as a developmental defect called accelerated villous maturation, defined as the presence of small or hypermature villi for the gestational period, again often accompanied by syncytial knots (284).

Not only is villous surface area reduced but the fetal microvasculature within the villi is frequently abnormal. Abnormalities include lesions that reflect fetal vascular malperfusion (Table 2), but there are others. The density of capillaries within the terminal villi is reduced, as is the amount of elongation branching and coiling (157, 345). All this further reduces the surface area for gas exchange. Vasculo-syncytial membranes denotes regions where the fetal capillary endothelium abuts the syncytium – this reduces the distance between fetal and maternal circulations to 1-2 µm. The vasculo-syncytial membrane to villous fetal capillary density is reduced in FGR, impairing fetal-maternal gaseous exchange.

Regions of syncytium can be denuded and the deficit covered over by fibrinoid material (348). In addition, there is reduced syncytial fusion events and increased production of syncytial knots (likely caused by hypoxia or reactive oxygen species) that are released into the maternal circulation (331). Underneath, there are decreased numbers of proliferating cytotrophoblasts, likely due to premature loss from cell death (331).

Non-infectious villitis, also called villitis of unknown etiology, is often present in term FGR (349). It is a pathological observation that is distinct from maternal or fetal malperfusion. There is significant infiltration of T-cells in the terminal and stem villi that extend to the villous stroma. Macrophages are also present, and they amplify the immune response. High grade villitis of known etiology occurring with FGR reportedly has strong associations with neurodevelopmental impairment and recurrence of FGR (350). However, it is possible studies reporting this link may be limited by confirmatory bias as reporting pathologists are unlikely to be blinded to the presence of FGR.

#### 5.3.6 Molecular perturbations in the placenta

A plethora of molecular changes occur in placentas from pregnancies affected by FGR. Many of them are likely driven by tissue ischemia and hypoxia caused by the many lesions just described. Increased apoptosis has been observed in the aging placenta and in cases of FGR (351). However, as discussed above, the considerable care must be taken in determining whether this is the cytotrophoblast or the syncytial layer.

FGR is also associated with chronic oxidative stress (with an increase in free radicals) (327), mitochondrial, and endoplasmic reticulum stress (352, 353). These processes are thought to promote cellular senescence of the trophoblast, or aging (346, 353). Senescent cells undergo cell-cycle arrest and shift away from oxidative phosphorylation towards glycolysis. The presence of senescence is injurious to the local environment – it leads to a senescence-associated secretory phenotype where increased secretion of matrix metalloproteinases degrades the extracellular membrane and increases pro-inflammatory cytokine release, further promoting an inflammatory microenvironment.

Nutrient exchange is compromised by reduced expression and activity of nutrient transporters, such as the amino acid (system A) transporter (354, 355). Placental hypoxia, which is present in FGR, downregulates system A transporter expression (356). Furthermore, there is mechanistic evidence that placental expression of amino acid transporters are reduced before the development of FGR (356) and inhibiting the transporter can reduce fetal weight (357)]. In contrast, there is no evidence of decreased efficiency of glucose uptake, nor change in expression of glucose transporter 1, the main glucose transporter (346, 355). However, the overall amounts of glucose received by the fetus are probably still reduced because of lower total villi surface area in the growth restricted placenta. Similarly, the exchange of permeable molecules like gases (oxygen, carbon dioxide) is compromised by decreased villi surface, less loops of blood vessels within villi and longer diffusion distances.

#### 5.3.7 Circulating markers in fetal growth restriction

Identifying circulating biomarkers that either predict or identify FGR are avidly sought as it may be a path to reduce adverse outcomes, including stillbirth. Besides first trimester PAPP-A and hCG, there are a number of circulating biomarkers that are associated with FGR (249, 358). Many are likely to originate from the placenta (358). PIGF, or an increased sFLT1/PIGF ratio have an association with FGR, though they are far more strongly linked with preeclampsia. At 36 weeks gestation, an elevated sFLT1/PIGF ratio combined with an ultrasound identifying a small for gestational age fetus (<10^th^ centile weight) may be more than additive in predicting adverse clinical outcomes. Circulating markers strongly associated with FGR include circulating Serine peptidase inhibitor, Kunitz type 1 (SPINT1) (359) and a serum metabolite ratio (7, 250).

#### 5.3.8 Fetal adaptions

Faced with limited oxygen and nutrient availability the fetus diverts limited resources from healthy development and growth to survival. Growth trajectory slows and the blood flow is redirected from less vital organs to the brain (which has the highest metabolic needs of any fetal organ), heart and adrenal glands. There is increased consumption of glycogen stores, mainly from the abdomen and liver. This results in asymmetrical growth where the head circumference to abdominal circumference ratio increases (asymmetrical growth).

Many of the physiological (e.g. increased red cell production), metabolic, endocrine and molecular adaptive changes in the fetus may have lasting effects (“fetal programming”). There is extensive evidence that these adaptations predispose to increased health risks in later life (7). Epidemiological studies focus on the relationship between measurements at birth, such as birth weight and placental weight, and the experience of disease in later life (360). Animal models employing multiple species have addressed the effects of dietary modification, genetic manipulation, the administration of glucocorticoids and other drugs to the mother, and environmental modifications (such as exposure to low partial pressure of oxygen or higher levels of carbon monoxide) (361). A detailed review of the developmental origins of health and disease is outside the scope of the current paper, but the review articles cited above address many of these issues in detail.

**Figure 7:**
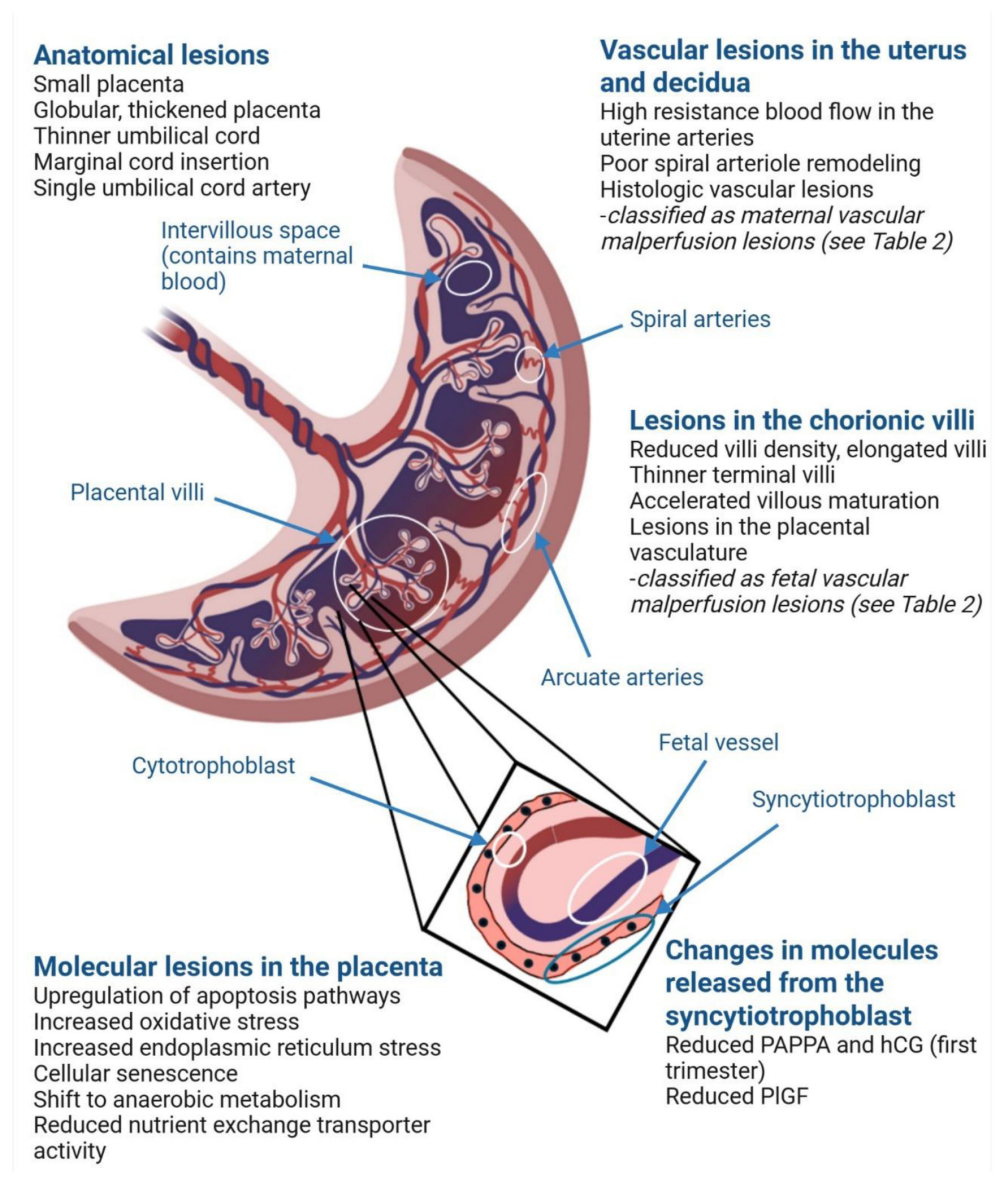
Overview of placental lesions associated with fetal growth restriction. PAPPA, pregnancy associated plasma protein-A; hCG, human chorionic gonadotropin; PlGF, placental growth factor. *Created using images from a licensed Biorender.com*.

In very severe placental dysfunction, significant hypoxia and increased circulating lactic acid can cause the fetus to decompensate, which provokes highly pathological responses. The stressed fetus may move less (which can be reported as reduced maternal perception of movements or assessed by ultrasonic examination) and there may be fetal cardiac dysfunction (seen on Doppler ultrasound in the clinic as reduced forward flow during atrial systole in the ductus venosus, a vessel that feeds oxygenated blood into the atrium of the right heart (362)). Neuronal hypoxia and acidemia perturbs autonomic signaling that regulates fetal heart rate (seen in the clinic as abnormal fetal heart rate patterns such as reduced short term variability or heart rate decelerations on electronic analysis of the fetal heart rate). With unrelenting placental insufficiency, the final outcome can be stillbirth. An overview of the anatomical and molecular changes associated with fetal growth restriction is provided below (Figure 7).

### 5.4 Preeclampsia

Preeclampsia is a multi-system disorder. Like FGR, it is a placental disease and shares many similar pathological features. Unlike FGR, it profoundly affects the mother – circulating factors released from the preeclamptic placenta incites widespread maternal endothelial and vascular injury (363, 364). This causes hypertension and can cause severe injury to many maternal organs (brain, kidneys, lungs, haematological system, or the liver). Very severe maternal organ injury can cause death or permanent morbidity. The placental disease can also result in FGR (which often co-exists with preeclampsia), placental abruption (the placenta shearing off the uterus before birth) or stillbirth (363, 364).

Having had preeclampsia leaves a lifelong legacy of an increased risk of chronic illnesses for the mother, especially cardiovascular morbidity. Over her lifetime there is a very high risk of developing chronic hypertension (363), and a 2-4 fold increased risk of stroke, heart and renal failure, and death from cardiovascular disease (365). It is also associated with an increased risk of dementia, especially vascular dementia (366). It remains hotly debated whether preeclampsia rewires maternal physiology putting her at risk of these conditions, or the mother already had an adverse vascular system which has put her at risk of both preeclampsia and the chronic health conditions that occur in later life (meaning preeclampsia was not part of the causal pathway leading to the chronic diseases) (367).

#### 5.4.1 Different subtypes

While preeclampsia of all types has common similarities recognizable to clinicians (hypertension and specific patterns of injury to organs) there is an increasing move to consider the condition as different subtypes, or syndromes (368). It is proposed that the underlying pathophysiology driving the different subtypes may be very different, an analogy offered by a US expert panel being type I and II diabetes causing hyperglycemia (368). An exemplar of different subtypes is preeclampsia arising preterm versus term gestation (364, 368, 369). For the former, the early implantation failure and placental lesions are more severe. In contrast, placental pathology is often milder for preeclampsia arising around term gestation, but significant underlying maternal vascular pathology tips women into manifesting clinical disease (Table 3).

#### 5.4.2 Placenta in preeclampsia – stage 1 of preeclampsia

Many placental lesions seen in FGR (detailed above) are also present in preeclampsia. The failure of spiral arteriole remodeling leading to reduced intervillous blood flow, (where jets of blood arising from arteriole luminal narrowing causes turbulent blood flow, leading to villous injury and ischemia) is considered a key feature in the early pathogenesis of preeclampsia, especially preterm disease (19, 374). Furthermore, many of the other maternal vascular lesions discussed for FGR are present in preeclampsia, including many of the maternal vascular malperfusion lesions (370) such as acute atherosis (375–377).

Reduced placental perfusion caused by these maternal vascular lesions leads to what is now often coined ‘syncytiotrophoblast stress’ (stage 1, or placental disease) (335, 364, 378). It arises because there is uteroplacental mismatch, where demand for nutrients outstrips maternal vascular supply (364). Syncytiotrophoblast stress is characterized by significant cellular pathology in the syncytiotrophoblast layer, such as endoplasmic reticulum stress, oxidative stress causing mitochondrial damage and dysregulated metabolism (335, 378).

The innate and adaptive immune system is thought to be intimately involved in facilitating healthy placental implantation and, conversely, their dysregulation is part of the pathogenesis of preeclampsia. Decidual specific natural killer cells and decidual specific T-regulatory cells have a critical role promoting immune tolerance to the fetus. Decidual specific natural killer cells regulate trophoblast invasion via secretion of angiogenic growth factors, cytokines and chemokines (34, 379). T-regulatory cells facilitate immune tolerance via antigen presentation, secretion of cytokines and even facilitate vascular remodeling (378, 380). Immune tolerance mismatch may lead to faulty placental implantation and preeclampsia. For instance, it has been shown that various polymorphisms of genes coding the killer immunoglobin like receptor (KIR, expressed on decidual natural killer cells) and HLA-C expressed on the surface of extravillous trophoblasts are associated with an increased risk of preeclampsia (36). There is epidemiological evidence to suggest that a shorter duration of immune system exposure to paternal pathogens (such as seminal fluid) is associated with an increased risk of preeclampsia. Furthermore, reduced sperm (or seminal fluid) exposure - uch as pregnancies conceived from donor insemination (381), donor sperm (382), donor oocyte (383); a first time pregnancy or conception after a shorter duration of co-habitation (384) - are all associated with an increased risk of preeclampsia.

#### 5.4.3 Endothelial dysfunction and vascular injury – Stage 2 of preeclampsia

It is postulated that syncytiotrophoblast stress in the placenta is the cause for the release of many factors into the maternal circulation (385), such as sFLT1 and other anti-angiogenic factors, extracellular vesicles and nucleic acids. They circulate widely and cause maternal endothelial dysfunction and vascular injury. Some of these factors may act directly on the endothelium, or activate the maternal immune response, leading to the release of pro-inflammatory cytokines that potentiate the adverse endothelial response.

The endothelium lines the lumen of the vascular smooth muscle of blood vessels. A number of adverse pathological processes occur with endothelial dysfunction and they can all compromise perfusion of downstream organs leading to injury. There is increased surface expression of cell adhesion molecules, notably vascular cell adhesion molecule-1 (VCAM-1 – upregulation can be readily reproduced *in vitro* by administering pro-inflammatory cytokines such as tumor necrosis factor-α to endothelial cells (386)). The usual role of VCAM-1 is to promote transendothelial migration of leukocytes into areas of infection but in vascular pathologies, VCAM-1 can cause an inflammatory mesh (387), ensnaring leukocytes, consuming coagulating factors and causing pathological complement and platelet activation. The dysfunctional endothelium likely reduces the release of vasorelaxation factors which then act locally, such as nitric oxide (a soluble gas which relaxes the underlying vascular smooth muscle), prostacyclin and endothelium-derived hyperpolarizing factor (388, 389). Endothelial injury also causes elevated release of the peptide endothelin-1 by 2-3 fold (390, 391), a potent vasoconstrictor that can act locally on the underlying smooth muscle cells but also circulate in the blood. Endothelin-1 is also proposed to act on the placenta via cognate receptors expressed on trophoblast to increase endoplasmic reticulum stress (392). Finally, with endothelial dysfunction there is reduced gap junction formation between endothelial cells (389), resulting in vascular leakiness and oedema which is a well-recognized clinical feature of the condition (393).

#### 5.4.4 Placental derived circulating factors that cause the endothelial dysfunction

Of all the placentally derived circulating factors, the evidence seems most compelling to implicate sFLT1 as an important driver of the pathogenesis of preeclampsia. sFLT1 is released from placenta in all pregnancies, where levels rise across gestation (241). In preeclampsia circulating concentrations of sFLT1 is elevated across gestation (241). sFLT1 is a truncated form of the vascular endothelial growth factor receptor 1 (VEGFR1). It comprises the extra-cellular domain but does not contain the transmembrane domain or the intracellular kinase domain (394). It is produced by alternative splicing of the VEGFR1 gene, then secreted by the syncytiotrophoblast into the maternal circulation (395).

Relative circulating levels of sFLT1 correlate with the clinical severity of disease (396–398). sFLT1 is increased many weeks prior to the onset of preeclampsia (241). When compared to gestationally matched controls, relative levels are far higher in preterm preeclampsia, a disease variant with particularly high morbidity (398). Conversely, preeclampsia without high levels of sFLT1 is associated with more favorable clinical outcomes (399). The link between sFLT1 and preeclampsia is sufficiently robust that it is now used as a diagnostic adjunct: a normal sFLT1/PIGF ratio is used to rule out the presence of the condition (242) in women where it is clinically suspected.

**Figure 8:**
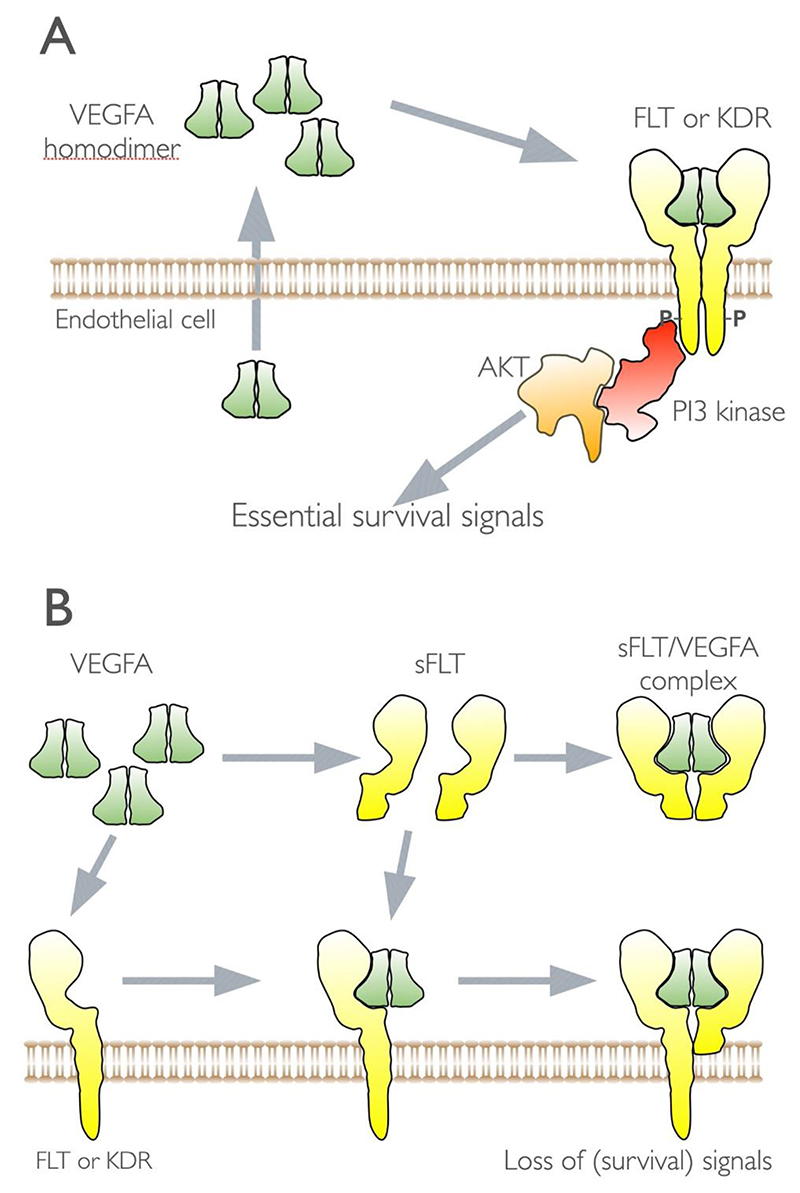
Schematic illustration of VEGFA signaling and the dominant negative action of sFLT1. VEGFA is secreted from endothelial and binds with high affinity to the VEGF receptors (FLT1 or KDR). This leads to dimerization and autophosphorylation of the receptor (-P). A signaling cascade is triggered which activates PI3 kinase and AKT, promoting endothelial cell survival. B) In the presence of sFLT, VEGFA (and PlGF) are bound in a complex which prevents their binding to cell surface receptors. Thus, sFLT can act as a competitive inhibitor. However, sFLT can also bind to the cell surface receptors and form a heterodimer. As sFLT lacks the intracellular kinase domain, autophosphorylation cannot occur and activation of the full-length receptor is blocked and the survival signals are not generated. Figure modified from Charnock-Jones 2016 (406).

Furthermore, it is biologically plausible that sFLT1 may have an important role in preeclampsia. It functions as a ligand trap, sequestering VEGFA, VEGFB and PlGF preventing binding to their cognate receptors to maintain healthy endothelial and vascular function (395, 398). VEGFA signaling induces nitric oxide formation, reduces vasoconstrictor signaling and neutralizes reactive oxygen species (400). Numerous animal models have shown that administering sFLT1 recapitulates aspects of the condition, such as hypertension and proteinuria (240). However, sFLT1 also acts as a dominant negative receptor which directly inhibits autocrine VEGF receptor signaling, which is required for endothelial maintenance (401–403), Figure 8. This is consistent with the observation that administration of bevacizumab (Avastin), an anti-VEGFA monoclonal antibody used to treat malignancies, can cause hypertension and proteinuria (404). Finally, a fetal genome-wide association analysis reported that the only sequence variants associated with preeclampsia were near the *FLT1* gene (405).

PIGF is an angiogenic factor that is a member of the VEGF family. Released by the placenta, it binds and activates VEGFR1 to promote healthy maternal vascular function (in contrast, VEGF binds to both VEGF receptors 1 and 2) (407). In normal pregnancies, there is a progressive rise in PIGF that peaks at around 30 weeks gestation, then declines (408). PIGF concentrations are reduced in preeclampsia. Significantly, levels are low many months prior to the onset of preeclampsia and, as noted above, is now measured as part of clinical care in some countries (206).

There are other vasoactive factors that are increased in the circulation with preeclampsia. Soluble endoglin is elevated in preeclampsia (409). It is the extracellular domain of endoglin, a co-receptor that promotes transforming growth factor-α signaling on endothelial cells (which induces angiogenesis) (410). Cleaved off the placenta by matrix metalloproteinase-14 into the circulation (411), soluble endoglin antagonizes transforming growth factor-β signaling on the endothelium, causing endothelial dysfunction (410). There is increased circulating agonistic autoantibodies against the angiotensin type II receptor 1 (AT_1_) in preeclampsia (412). They act as agonists to the AT_1_ receptor on vascular smooth muscle and promote vasoconstriction (413). AT_1_ autoantibodies also suppress circulating renin and aldosterone (414) and their administration to mice can produce features of preeclampsia (415).

Preeclampsia is associated with both placental and systemic pro-inflammatory immune response. There are elevated circulating levels of pro-inflammatory cytokines such as tumor necrosis factor-α and interleukin-6 (416). These pro-inflammatory cytokines may be released systemically by an activated maternal immune system but they may also be directly released from the placenta. One mechanism of placental release of pro-inflammatory cytokines is via the activation of toll-like receptors 3 and 4 on the placental surface. There is increased expression of these receptors in preeclamptic placentas (417).

In preeclampsia, extracellular vesicles are released in excess into the circulation and are likely to contribute to endothelial dysfunction (418). Extracellular vesicles are cell derived membranous vesicles and is a term that encompasses different vesicle types. Three main vesicle types are exosomes (the smallest type 50-150nm), microvesicles and apoptotic bodies (released from cells undergoing apoptosis) (418). In endothelial cells, it has been shown *in vitro* that extracellular vesicles are internalized and may have antiangiogenic and hypertensive effects (418). An interesting line of research is that the release of the extracellular vesicles is not simply a result of syncytiotrophoblast cellular turnover (where the particles are effectively waste products), but they are deliberately released to allow the placenta to communicate with distant organs (419), especially the immune system to promote immune modulation (420, 421). If this is true, then not all extracellular vesicles are harmful – some may be facilitating healthy pregnancy outcomes. Indeed, many of these vesicles are packaged with cargo that includes proteins, lipids and many RNA species (mRNA, miRNAs, tRNAs) (420). It is possible that outward facing proteins studded on the surface of these vesicles could facilitating organ specific targeting, and to promote endocytosis (leading to fusion with endosomes and release of its cargo) (421). If so, it is plausible that this communication could go awry in preeclampsia where the result is systemic inflammation.

#### 5.4.5 Maternal organ injury

The widespread endothelial and vascular dysfunction and compromised blood flow leads to injury to many maternal organs. The most severe outcome from injury to any of these organ systems is death and hypertensive disorders of pregnancy account for 14% of all maternal deaths globally (422).

The kidneys are most often affected by preeclampsia, manifesting clinically as proteinuria, or elevated blood levels of creatinine or urea. That the kidneys may be the most often affected in preeclampsia might be explained by the fact that the glomerular endothelial cells express high levels of VEGF (antagonized in preeclampsia by sFLT1) (423). Historic studies of renal biopsies from women with preeclampsia often show a distinct histopathological feature that was once considered pathognomonic of the condition: glomerular endotheliosis. This is swelling of endothelial cells in the kidneys, obliteration of fenestrations and occlusion of capillary vessel lumens (424).

Liver involvement is suspected clinically by symptoms of right upper quadrant or epigastric pain, and is confirmed by elevated circulating levels of liver enzymes in maternal blood (spillage of enzymes into the circulation from affected hepatocytes). The liver pathology includes fibrin deposition in vessels and hepatic sinusoids, periportal hemorrhage and hepatocyte necrosis (425). At worse, fatal liver rupture or liver failure can occur.

The neurological system may be affected. In the brain there may be compromised autoregulation of the cerebral circulation and increased permeability of the blood-brain-barrier. This can lead to significant brain oedema, leading to symptoms, such as visual disturbances or even cortical blindness. Clinically, eclampsia can occur (tonic-clonic seizures) and this can lead to a life-endangering intracerebral bleed (426). Eclampsia is an important cause of mortality in low- and middle-income countries (426).

**Figure 9:**
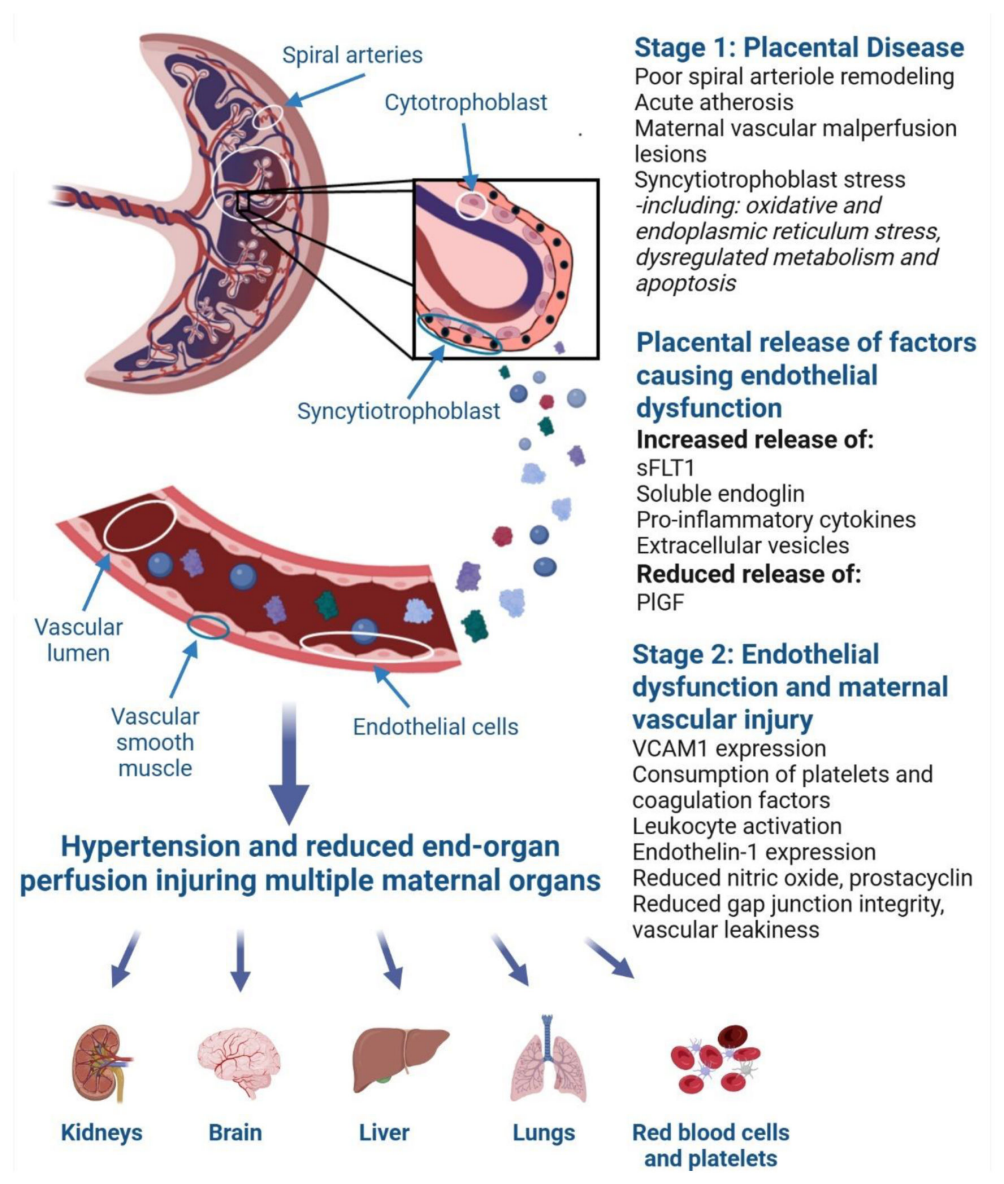
Overview of the pathogenesis of preeclampsia. sFLT1, soluble fms-like tyrosine kinase 1; VCAM1, vascular cell adhesion molecule 1; PlGF, Placental growth factor. *Created using images from a licensed Biorender.com*.

There may also be significant fluid leakage into the lungs leading to pulmonary edema. This can arise due to capillary leakage but also exacerbated iatrogenically if women with preeclampsia are administered too much intravenous fluid. The hematological system can also be involved in preeclampsia where anemia develops due to the lysis of red blood cells, and platelet counts fall due to platelet activation and aggregation. The pathogenesis of preeclampsia and associated changes in the placenta and maternal organs is summarized below (Figure 9).

### 5.6 Gestational diabetes

Gestational diabetes mellitus (GDM) is a common complication in pregnancy, affecting approximately 14% of pregnancies worldwide (427). Prevalence varies by region, with rates as high as 25% in South-East Asian countries (428) and 7-8% in the UK and USA (427). GDM incidence has increased by more than 30% over the past two decades due to factors such as obesity, advanced maternal age, and insulin resistant diseases such as polycystic ovarian syndrome (429). GDM poses significant risks to both mother and fetus during pregnancy including hypertensive disorders in the mother and excessive fetal growth and adiposity. Furthermore, GDM pregnancies heighten the likelihood of adverse obstetric outcomes including preterm-labor, preeclampsia, caesarean section delivery, macrosomia and shoulder dystocia (430). Beyond pregnancy, individuals affected by GDM, both mothers and offspring, face an increased risk of developing diabetes, obesity, and cardiovascular disease later in life (431).

#### 5.6.1 Macroscopic and microscopic findings in a GDM placenta

GDM is consistently associated with larger placentas in terms of weight, volume, thickness and diameter (432–435). The increase in placental size is believed to be a result of increased villous tissue and intervillous space. The elevated placental weights in GDM pregnancies often correlate with higher birth weights, possibly stemming from increased placental nutrient delivery or independent effects of GDM on both placental and fetal growth due to maternal hyperglycemia.

At the cellular level, all trophoblast types (i.e., cytotrophoblast, syncytiotrophoblast and extravillous trophoblast) are increased in GDM (434, 436). This may be explained by both increased trophoblast proliferation and reduced cell death as indicated by the staining patterns of Ki-67 and TUNEL respectively (437, 438), although some studies have also reported an increase in apoptosis. Histopathologically, GDM placentas commonly exhibit villous immaturity, which is characterized by reduced number and surface area of terminal villi but increased abundance of immature intermediate villi. Additionally, villous hypervascularization (i.e., higher number of branches per capillary in terminal villi) is consistently associated with GDM placentas (435, 436) which may contribute to the greater incidence of placental vascular lesions (histological changes related to blood flow) (439). It is important to note that many of these histopathological reports on GDM placentas are based on high-risk cases or related to case-control studies selected from those with abnormal findings. Moreover, in these studies, the extent to which the reporting pathologist was blinded to the clinical condition remains uncertain, introducing a potential source of bias. In a study of histological examinations of term placentas performed in an unselected population of over 1000 women, pathologists completely blinded to all clinical information reported that only 20% of GDM placentas exhibited histological abnormalities which was actually lower than the proportion (28%) in placentas from pregnancies with a normal outcome (283). This suggests that the majority of GDM placentas at term lack histological lesions, and that histopathological findings do not always correlate with abnormal outcomes.

#### 5.6.2 Molecular phenotype of the GDM placenta

In healthy pregnancies, maternal insulin sensitivity decline in the second trimester of pregnancy. This usually presents in the form of reduced sensitivity to insulin-mediated glucose uptake in peripheral tissues such as the skeletal muscle and adipose tissue. Pregnancy-induced insulin resistance is regulated in part by placental secretion of hormones such as estrogen, progesterone, leptin, placental growth hormone and placental lactogen (440). This leads to a modest increase in circulating glucose, which is readily transported across the placenta to support fetal growth. To ensure that glucose homeostasis is maintained, maternal pancreatic β cells undergo hypertrophy and hyperplasia and increased glucose-stimulated insulin secretion (441). In GDM pregnancies, women initially adaptively maintain normoglycemia in early pregnancy because their β cells can increase insulin production. However, by late pregnancy as insulin resistance increases due to placental hormones, the insulin response becomes inadequate. Some form of β-cell defect likely existed in these women before pregnancy particularly in those with co-morbidities such as obesity, but this is only clinically manifested with pregnancy-associated insulin resistance, resulting in hyperglycemia. Although hyperglycemia is the most widely recognized metabolic derangement in GDM, other nutrients such as lipids and amino acids may also be elevated (442). These metabolic changes significantly impact upon the placenta and fetus resulting in their excess growth.

Maternal hyperinsulinemia and hyperglycemia in a GDM pregnancy have several consequences on the placenta with implications for fetal growth. Circulating insulin binds to insulin receptors on the maternal-facing microvillus membrane of the syncytiotrophoblast (443). Insulin itself does not cross the placenta as it is rapidly degraded by proteases in the placenta (444). Insulin binding to its receptor on the placental surface triggers the phosphorylation of the insulin receptor substrate-1 (IRS-1). This further initiates a phosphorylation cascade leading to the activation of the PI3K/Akt and Erk-MAPK pathways. These pathways regulate cellular metabolism and growth, respectively. In classical insulin-sensitive tissues such as the skeletal muscle and adipose tissue, insulin-mediated PI3K/Akt signaling mediates glucose uptake via trafficking of the glucose transporter (GLUT) 4 to the plasma membrane. In the placenta, GLUT4 levels are minimal beyond the first trimester and therefore insulin does not directly influence glucose transfer to the fetus (445). Instead, the placenta is highly abundant in the high affinity glucose transporter GLUT1, which allows rapid uptake and transfer of glucose across the placenta. Unlike GLUT4 however, GLUT1 does not respond to insulin (446) and, therefore, placental glucose transport is primarily driven by the maternal-fetal glucose concentration gradient. The fetal pancreatic β cells respond to elevated glucose by secreting insulin which promotes nutrient accretion in fetal tissues leading to increased fetal growth. Placental amino acid uptake and fatty acid esterification are increased in response to maternal hyperinsulinemia and the increased amino acids and lipids are transferred to the fetus, further exacerbating the insulin response (447, 448). Interestingly, in GDM pregnancies, although the maternal peripheral tissues develop insulin resistance (449), the placenta remains insulin responsive (450).

The importance of fetal hyperinsulinemia in regulating fetal growth was elegantly illustrated in animal models. Chronic insulin infusion in fetuses of pregnant rhesus monkeys increased fetal weight (451), whereas fetal lambs exposed to streptozotocin, which induces β-cell destruction and diminished insulin production, exhibited FGR (452).

**Figure 10:**
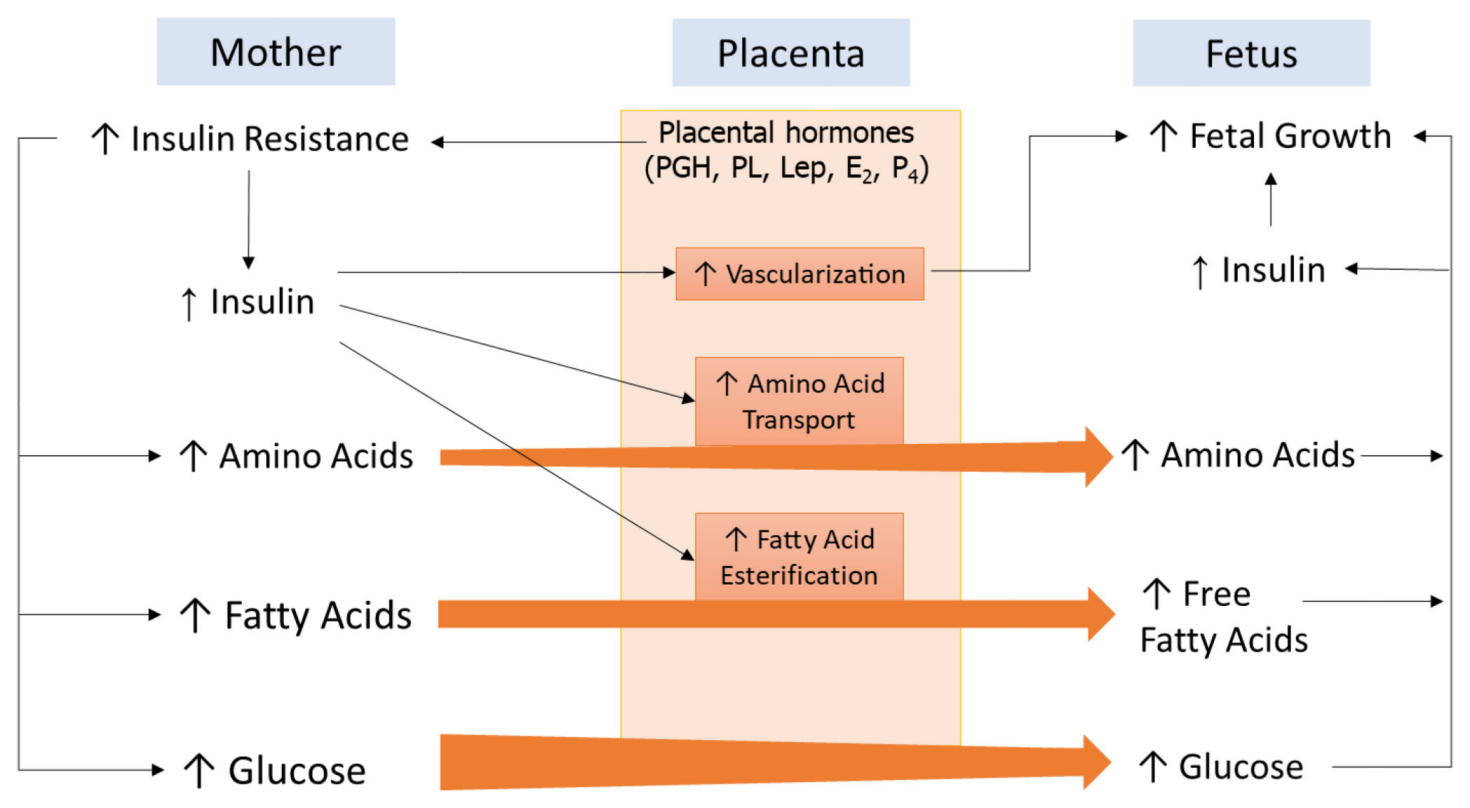
Schematic representation of the metabolic effects of maternal gestational diabetes on placental function and fetal growth. Maternal insulin resistance diverts circulating nutrients (amino acids, fatty acids and glucose) across the placenta. Maternal hyperinsulinemia promotes placental endothelial vascularization, trophoblast amino acid transport and fatty acid esterification. The resulting increase in nutrient accumulation in the fetus promotes fetal insulin secretion leading to excess fetal growth. PGH, placental growth hormone; PL, placental lactogen; Lep, leptin; E_2_, estrogen; P_4_, progesterone.

Fetal hyperinsulinemia has also been proposed to mediate placental angiogenesis which may explain the observed hypervascularization in the GDM placenta (435, 436). Fetal insulin stimulates insulin receptors on the feto-placental endothelial cells and promotes endothelial network formation (453) Molecularly, this involves activation of the PI3K pathway regulating endothelial nitric oxide synthesis which stimulates endothelial tube formation, increased extracellular matrix degradation and cytoskeletal rearrangement which collectively promote angiogenesis (453, 454). In summary, the placental response to maternal hyperglycemia and hyperinsulinemia in a GDM pregnancy is dependent on fetal hyperinsulinemia resulting in placental hypervascularization as well as increased nutrient transfer (Figure 10).

### 5.7 Spontaneous preterm birth

Spontaneous preterm birth (sPTB), like stillbirth, is the end point of diverse pathophysiological pathways (455). There are a number of these pathways which involve the placenta, both directly and indirectly.

#### 5.7.1 Retroplacental haemorrhage and sPTB

Acute placental dysfunction can lead directly to sPTB through placental abruption and this is due to direct and indirect (via prostaglandins) effects of thrombin to stimulate myometrial contraction (456). Chronic bleeding in the interface between the decidua and the placenta can lead to preterm labour through activation of proteases and pro-inflammatory cytokines stimulated by the release of thrombin which promote pPROM and myometrial contraction (457).

#### 5.7.2 Placental infection and inflammation and the risk of sPTB

Colonisation of the mother with Mycoplasma hominis, Ureaplasma urealyticum or Ureaplasma parvum is associated with an increased risk of preterm birth (458) and U. urealyticum is found more commonly in the placenta in cases of spontaneous preterm birth (459). Acute inflammatory findings (neutrophil infiltration) in the placenta, umbilical cord (funisitis) and fetal membranes (chorioamnionitis), are strongly associated with spontaneous preterm labour – particularly at extreme preterm gestational ages – and this is usually secondary to ascending infection (460) and not a marker of placental dysfunction, *per se*. Sterile inflammation is also associated with sPTB (461): T cell infiltration of the placenta (villitis of unknown etiology) and fetal membranes (chronic chorioamnionitis) frequently co-exist, are associated with spontaneous preterm labour – particularly in the late preterm period – and are thought to reflect maternal immune rejection of the placenta and membranes.

#### 5.7.3 Placental insufficiency and the risk of sPTB

There is a large body of evidence which indicates that a proportion of sPTB is due to prolonged placental insufficiency. Early evidence for an association used first trimester assessment of the fetus and placenta, demonstrating that smaller fetal size and low levels of the placentally-derived protein, PAPP-A in the first trimester were associated with the subsequent risk of spontaneous preterm birth (293, 323). Maternal serum levels of AFP and hCG measured between 15 and 20 weeks gestation were also associated with an increased risk of sPTB and the association with hCG significantly differed in relation to the severity of the degree of prematurity, being specific for births between 24 and 28 weeks gestation but not later gestations (462). However, these associations, while shedding light on the complexity of sPTB and the role of the placenta, were not strong enough to provide clinically useful prediction: adding maternal biomarker levels to predictive models which included maternal characteristics generated models that were only moderately predictive. However, the combination of AFP and PAPP-A in the first trimester, either in a model or as a ratio, was moderately associated with the risk of spontaneous preterm birth (AUC 0.67 to 0.69) in a cohort of unselected nulliparous women in the UK (463) and its strength of prediction is comparable to the combination of fetal fibronectin and ultrasonically measured cervical length measured 22-30 weeks (AUC 0.67) in a cohort of unselected nulliparous women in the USA (464). Hence, first trimester placental biomarkers yield a comparable prediction to more widely recognized second trimester screening methods for sPTB.

#### 5.7.4 Trophoblast invasion and the risk of sPTB

Failure of spiral artery remodeling in both the decidua and myometrium is more commonly observed in cases of spontaneous preterm labor (465). Consistent with this finding, high resistance patterns of uterine artery Doppler flow velocimetry (both the presence of notches and higher mean pulsatility index) at around 20 weeks gestation were associated with the risk of spontaneous preterm birth, although associations were weaker than for medically indicated preterm birth (466). Animal studies have also highlighted the importance of decidual function, as genetic manipulation of mice to promote decidual senescence increases the risk of spontaneous preterm birth, which was reversed with the mTORC1 inhibitor rapamycin (467).

#### 5.7.5 Placental histopathology and sPTB

Aside from the effects of ascending infection on the placenta, there is some evidence for associations between placental histopathological abnormalities and sPTB. However, the data are inconsistent, and some associations may be the consequence rather than the cause of sPTB. An increased frequency of maternal vascular malperfusion and delayed villous maturation in cases of sPTB in the absence of inflammation has been reported (468). However, a study reporting the results of placental histopathology where the pathologist was blinded to clinical outcome demonstrated only an increased frequency of delayed villous maturation in cases of sPTB and this was interpreted as being likely to reflect the earlier gestational age of delivery, i.e. a consequence rather than a cause of the sPTB (469). Other studies have shown that fetal vascular malperfusion and maternal vascular malperfusion were more frequent in induced PTB but they were not associated with sPTB (470). While delayed villous maturation could be a consequence of sPTB, two other studies reported an increased proportion of cases with accelerated placental maturation in the context of sPTB (468, 470) which cannot be explained as being a consequence of earlier delivery.

#### 5.7.6 Other evidence linking placental insufficiency, FGR and sPTB

**Figure 11:**
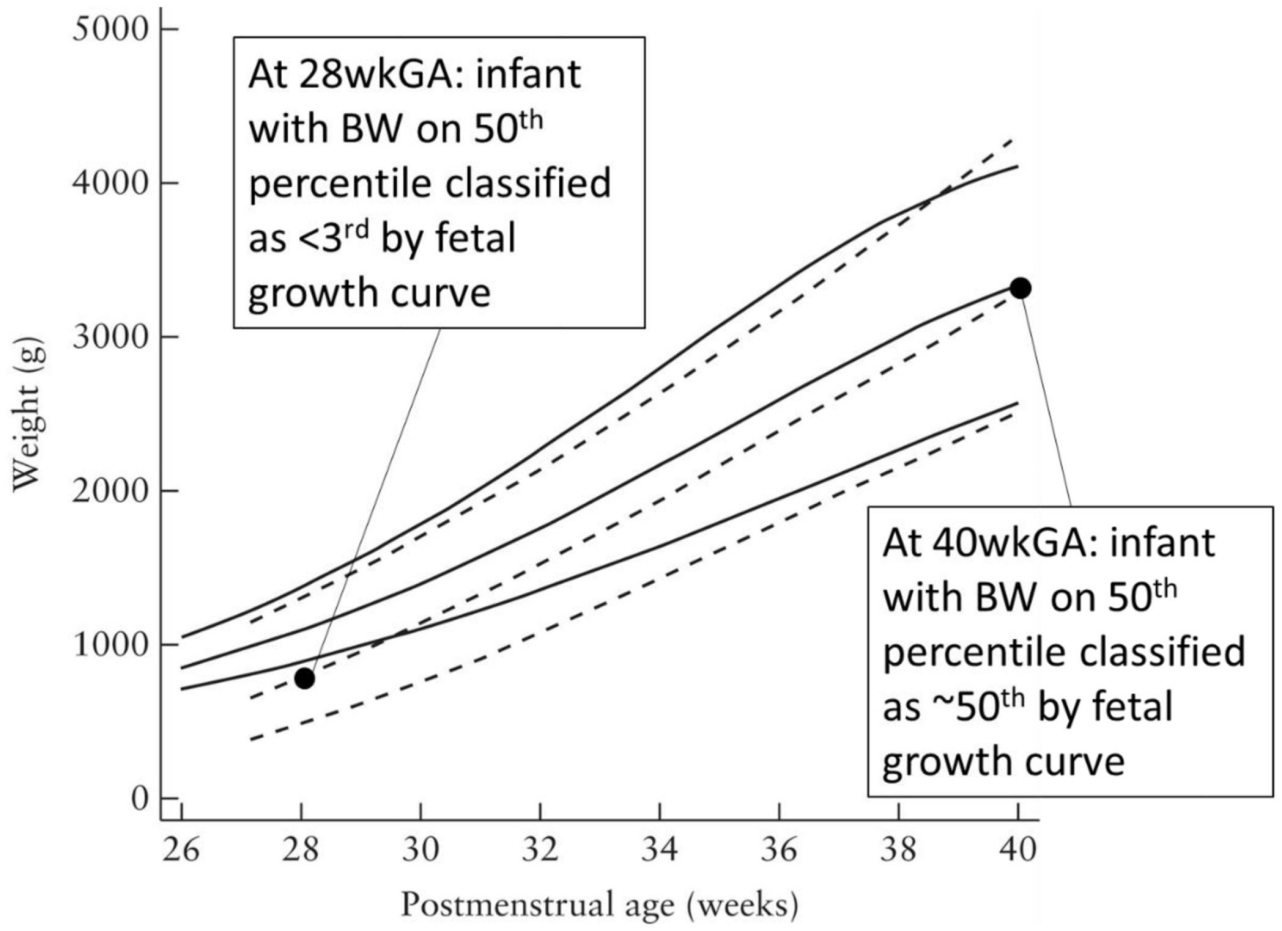
The dashed lines represent the 3^rd^, 50^th^ and 97^th^ percentile of observed birth weight across the range of gestational age. The solid lines represent the 3^rd^, 50^th^ and 97^th^ percentile of ultrasonically estimated fetal weight. Whereas the distributions are very similar at term, the distribution of observed birth weights is lower than the ultrasonic estimated fetal weights of on-going pregnancies at the same gestational age in the preterm period. Figure modified from Smith et al. Best Pract Res Clin Obstet Gynaecol 2018; 49: 478-486 (476).

Two prospective cohort studies performing serial fetal ultrasounds showed that slower fetal growth between the second and third trimester was associated with an increased risk of spontaneous preterm birth (471, 472). Similarly, slower growth between 28 and 36 weeks gestation was associated with an increased risk of spontaneous early term labor (473). Slowing of growth is assumed in these cases to reflect placental dysfunction. Consistent with this interpretation, increasing maternal serum sFLT1, decreasing PlGF and increasing sFLT1:PlGF ratio between 20 to 28 weeks gestation were associated with an increased risk of subsequent spontaneous preterm birth, although the associations were stronger with medically indicated preterm birth (474). Pregnancies with the combination of being in the top 10% of sFLT1:PlGF and the lowest 10% of femur length growth had a 9-fold risk of sPTB. Similarly, high levels of sFLT1 and low PlGF at 36 weeks gestation were both associated with an increased risk of spontaneous early term labor (473). Collectively, these findings indicate that FGR due to placental insufficiency may be associated with an increased risk of spontaneous preterm birth. This concept is also supported by comparison of ultrasonic estimated fetal weights (EFW) and actual birth weights. If the growth of babies born preterm was the same as the babies ultimately born at term, it would be expected that the normal distribution of birth weights across the whole range of gestation would be the same as the normal distribution of ultrasonic EFW across the whole range of gestation. However, this is not the case. At term, the average EFW of on-going pregnancies is similar to the average birth weight of babies born in the given week. However, in contrast, at preterm gestational ages, the distribution of birth weights of babies delivered preterm is lower than the distribution of EFWs in on-going pregnancies at the same week of gestational age. For example, in a multinational prospective cohort study (InterGrowth21) the 50^th^ percentile of birth weight at 28 weeks was less than the 3^rd^ percentile of EFW among on-going pregnancies (475) (Figure 11). This observation indicates that the population of babies being born preterm is enriched with cases of FGR.

#### 5.7.7 Potential mechanisms linking fetal stress and sPTB

Dissecting the mechanisms linking placental dysfunction to sPTB is limited as the mechanisms which lead to spontaneous labour are still incompletely understood in humans (477). Unlike many species where labour is initiated by falling circulating levels of progesterone, there is no such fall in the human. It is speculated that there may be a “functional withdrawal” of progesterone and this could be due to changes in the relative abundance of progesterone receptor subtypes, but the factors which control this also remain incompletely understood.

**Figure 12:**
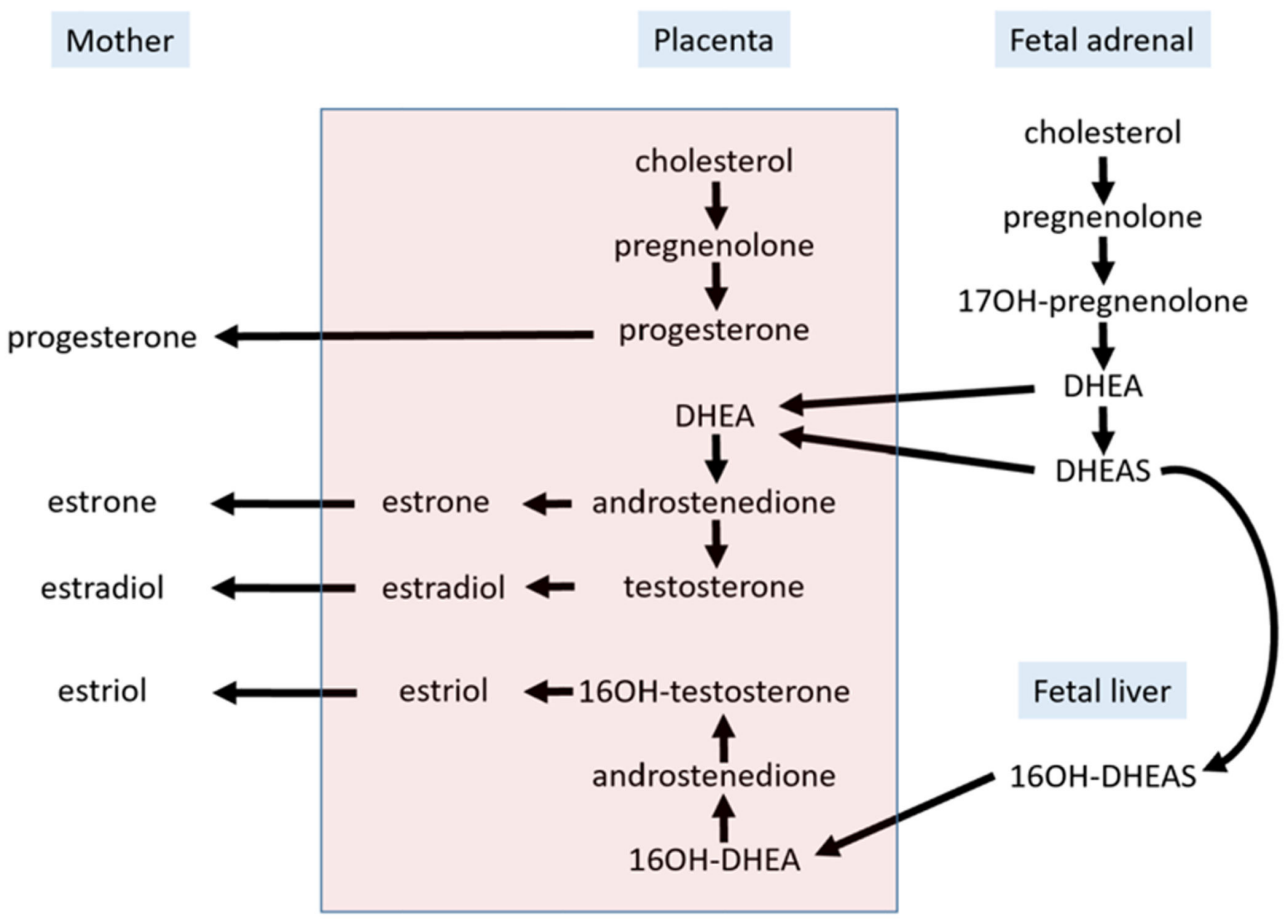
Schematic representation of integrated pathways of steroid biosynthesis in the fetus and placenta. DHEA denotes dehydroepiandrosterone and DHEA denotes dehydroepiandrosterone sulfate.

The endocrine functions of the placenta could potentially explain the connection between placental function and spontaneous preterm birth (Figure 12). Corticotrophin releasing hormone (CRH) is produced in human and other non-human primate placentas, but not other commonly used model species, and circulating levels are massively elevated in pregnant women compared to the non-pregnant state (478). CRH rises exponentially with advancing gestational age in human pregnancy and its binding protein declines in late pregnancy resulting in an increase in bioavailable CRH. The rise in CRH occurs earlier in women who deliver preterm and more slowly in women who deliver post-term (479). However, there is substantial overlap in maternal serum CRH comparing women who ultimately deliver preterm or term and, although differences are highly statistically significant, its clinical usefulness as a screening test is limited (480). Increased bioavailable CRH could promote parturition through multiple pathways, which directly and indirectly (through mediators, such as steroids and eicosanoids) stimulate multiple key targets, including the maternal (pituitary, adrenal, myometrium and cervix), fetal (pituitary, adrenal gland and liver) and extra-embryonic tissues (placenta and membranes), and the evidence is reviewed in detail elsewhere (481). Moreover, there is evidence linking placental dysfunction to increased placental production of CRH as umbilical cord blood levels of CRH were elevated 5-fold in fetuses with growth restriction (482).

Another endocrine pathway which could contribute to a link between placental insufficiency and sPTB is the production of estrogens. The massive increase in maternal estrogens during pregnancy has been known for ˜100 years, and research in this area identified the role of the fetal adrenal and liver, interacting with the placenta, to generate estrone, estradiol and estriol (483) (Figure 6). While administration of estrogens to primates does not induce labor, administration of androstenedione to pregnant rhesus monkeys at 80% of term gestation resulted in increased maternal levels of estrogen and this was associated with increased myometrial contraction and changes in the fetal membranes similar to those observed with physiological labor at term (484). There is extensive evidence linking production of estrogens by the feto-placental unit and placental insufficiency and measurement of estriol was used as a method of assessing fetal well-being in the 1970s (485). However, the method was supplanted with the development of ultrasound as an imaging modality and meta-analysis of studies indicates that, although low levels of urinary estriol was associated with an increased risk of complications, measurement of urinary estriol performed poorly as a predictive test (486). The effect of estrogen receptor activation in the uterus is generally to induce changes which promote parturition (477). However, lower levels of estriol, caused by placental insufficiency, could lead to increased estrogen signaling as estriol is a weak agonist and present in massive excess and can be antagonistic to the effects of full agonists, such as estradiol, on both nuclear and G protein coupled receptors (487, 488).

#### 5.7.7 The concept of “fetal rescue” and its relevance to stillbirth

**Figure 13:**
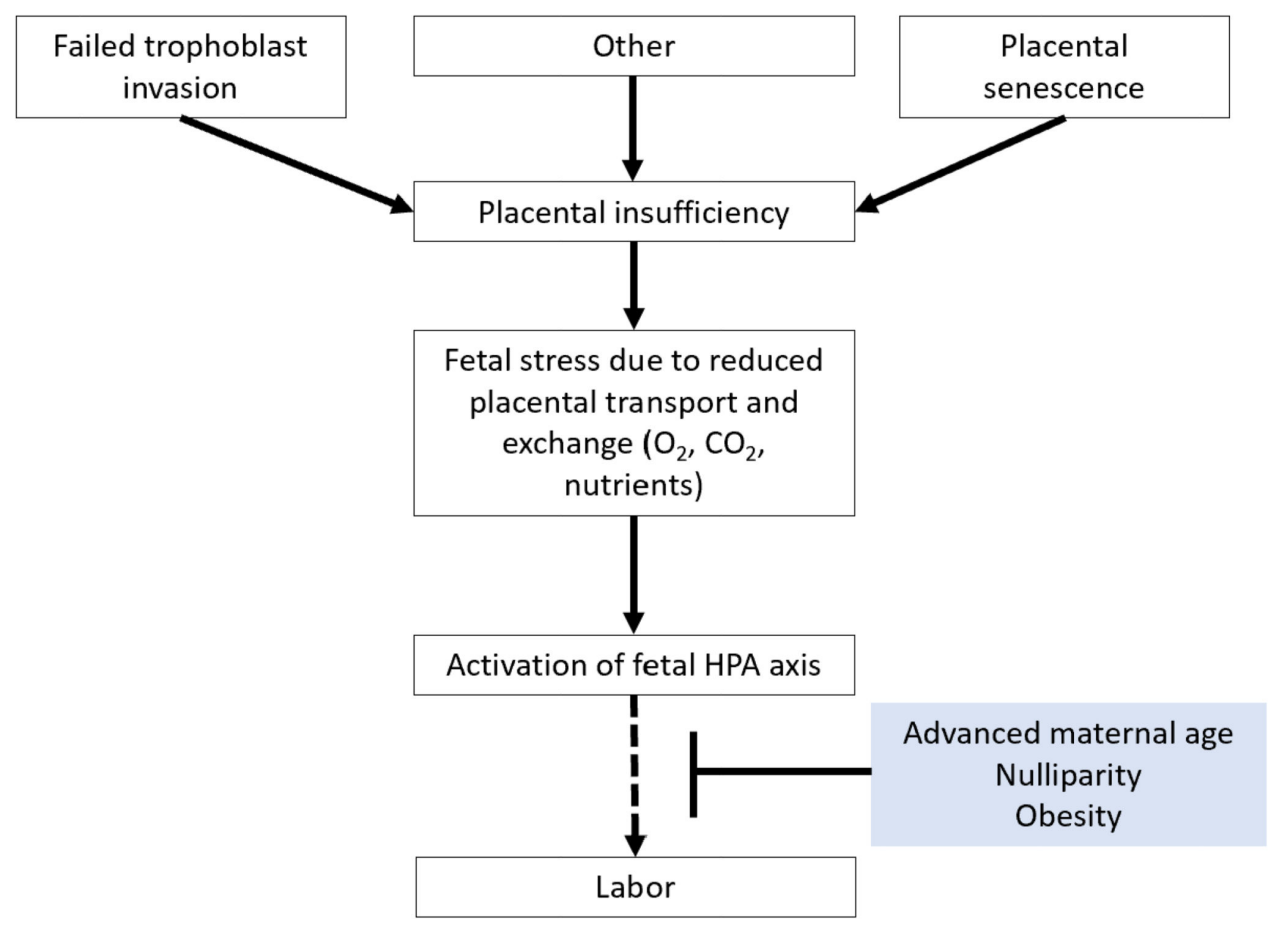
Schematic overview indicating the link between placental dysfunction, fetal stress and the initiation of labor and delivery. Maternal factors associated with an increased risk of stillbirth are highlighted (blue box) as potentially inhibiting the effect of activation of the fetal hypothalamo-pituitary adrenal axis to promote labor (broken line as mechanisms less well understood in humans than other model species, e.g. sheep). Hence, the above model could explain the relationship between these factors and the risk of stillbirth.

The data above indicate that both preterm and term labor may be associated with placental dysfunction. In the case of preterm labor, it makes sense that the fetus might activate pathways which initiate labor if faced with a hostile intra-uterine environment. However, while the initiation of labor and delivery might reduce the risk of intra-uterine fetal death, it is not certain that delivery enhances the overall chance of long term survival of the offspring, as preterm delivery is a major determinant of infant and child mortality. The associations described may also have implications for physiological labor at term. Even in normal pregnancies, markers of placental senescence increase with advancing weeks of gestational age at term, and this may be related to reduced antioxidant capacity of the placenta (489). Hence, it is possible that the physiological onset of labor at term is related, at least in part, to activation of fetal stress pathways secondary to placental senescence and this could in turn promote the onset of labor, via the pathways described above. A risk of such a system would be that failure of the fetal response to initiate parturition would lead to an increased risk of intra-uterine fetal death and stillbirth. Consistent with this are the well-recognized association between advancing gestational age at term and post term and the risk of unexplained stillbirth (490) and the fact that placentas from unexplained stillbirth and from post-term livebirths exhibit similar signs of aging-related changes (491). It is also interesting to note that several well recognized risk factors for unexplained stillbirth are associated with an increased risk of failure of induction of labor, including advanced maternal age and nulliparity (492, 493). Hence, the potential interrelationships between placental function, fetal stress and parturition may also be important in the etiology of stillbirth as well as the etiology of spontaneous preterm birth (Figure 13).

## 6 Animal models for human placental dysfunction

Animal models of human disease play an important role in evaluating possible therapeutic interventions. They also allow experimental manipulation which allow definition of the underlying mechanistic processes. It follows, therefore, that an essential aspect of an animal model is that it faithfully recapitulates the human condition. This is a substantial challenge when studying pregnancy-related disorders because placentation among mammalian species is extremely diverse. Even between closely related species, such as Old World monkeys and Great Apes (orangutans, gorillas, bonobo, chimpanzees, and man) there are notable differences. Within this group, the baboons and macaques share features such as endovascular trophoblast and spiral artery transformation as well as a true intervillous space thus making them the most similar to humans (494). However, working with non-human primates is ethically sensitive, very expensive and there are significant practical constraints. The long gestation period and the difficulty of genetic manipulation are particular disadvantages. Nonetheless, there are aspects of the underlying biological processes that are broadly shared among many species. These can be manipulated and are informative, even in species where placental development is substantially different from humans – such as rodents, where most of the work with animal models has been carried out.

### 6.1 Animal models of preeclampsia and fetal growth restriction

There are numerous animal models that have been used to investigate either the underlying pathology of pre-eclampsia or to test possible interventions. The reader is referred to the several recent reviews for a more detailed picture (495–498). These models can be divided into 4 broad categories i) some form of immunological intervention or perturbation such as administration of LPS (499) or the breeding of genetically distinct strains of mice (for example, the abortion-prone CBA/J×DBA/2 mouse (500)); ii) the administration of an exogenous agent - for example the nitric oxide synthase inhibitor, L-NAME, (501) or a specific protein (or a viral vector encoding a specific protein (240)); iii) genetic manipulation of a specific gene (502–504); iv) finally, a surgical intervention to directly perturb uterine blood flow (505, 506). Similarly, there are multiple models for fetal growth restriction with the most commonly used being a surgical intervention (typically in the sheep) (507) or dietary restriction (508– 511).

The key consideration when using of any of these models is to understand which step in the causal pathway of preeclampsia has been manipulated. For example, it is widely accepted that defective trophoblast invasion and transformation of the decidual spiral arterioles is one of the early and possibly causal events in preeclampsia. Therefore, if a model leads to perturbed invasion, then it may be suitable for the investigation of the earliest events. However, when choosing an animal model of preeclampsia, it needs to be remembered that the human, even when compared with most non-human primates, has very deep trophoblast invasion.

In the human, the extravillous trophoblasts invade the decidua whereas in rodents, the analogous cells are referred to as trophoblast giant cells (TGC) or the more generic ‘invasive trophoblast cell’ (512, 513). All these invasive cells are polyploid and the extent of the increased ploidy is greater in murine TGCs (hence, “giant cell”). Many features of the invasive mechanisms are conserved between species but the extent and some characteristics of the invasion vary. Specifically, in the mouse the invasion is shallower than in the rat (512–514). Nonetheless, both model species have been used to effectively investigate the role that decidual natural killer cells play in regulating invasion and, more specifically, vessel remodeling (515).

Several models are known to perturb trophoblast invasion and vessel remodeling. For example, treatment of rats on gestational days 13.5-16.5 with low dose LPS leads to TNFα mediated inflammation with an elevation in maternal white cell counts. Importantly in this specific context, the mean cross-sectional area of the spiral arteries was reduced. Similarly, in C1q^-/-^ mice there was a reduction of collagenolytic activity with impaired invasion. However, these authors did not quantify this nor the effect on decidual vessels (504).

One of the consequences of decreased decidual invasion is that the velocity of maternal blood flowing into the intervillous space is higher and the risk of spontaneous vasoconstriction and ischemia–reperfusion injury is raised. This damages the villi, releases villous debris and generates oxidative stress (19). Surgical models to reduce placental blood flow mimic some aspects of this and lead to many of the maternal changes observed in preeclampsia. The reduced uterine perfusion pressure (RUPP model – where a uterine artery is ligated) and its variants are widely used and have been effectively used to study the mechanism underlying the maternal features of preeclampsia (505, 506).

Preeclampsia has been described as a disease of the endothelium due to the widespread maternal endothelial dysfunction (516). Therefore, using models which show this feature allow the manipulation of candidate factor(s) that cause endothelial damage or testing of agents to mitigate of the effects of this damage. Such models and manipulations are useful in understanding the maternal effects but would not be expected to shed light on the ultimate cause of preeclampsia. For instance, in the rat RUPP model where circulating sFLT1 is increased (consistent with in vivo release due to placental hypoxia), chronic infusion of VEGF_121_ restored endothelial function and glomerular filtration rate and reduced the elevated blood pressure (517). Similarly in a uterine artery ligation model in the baboon, circulating sFLT1 is raised and there is endothelial dysfunction (elevated blood pressure, proteinuria and glomerular endotheliosis). These endothelial effects were ameliorated by administration of recombinant human PlGF (506). These studies strongly support the role of sFLT1 in the maternal endothelial dysfunction and they suggest that defects in placental perfusion (and hence oxygenation) can induce release of sFLT1. While these studies shed further light into maternal vascular pathophysiology of preeclampsia, they do not provide insights into the ultimate cause of preeclampsia which precede elevation of sFLT1.

Despite the marked differences in mammalian placentation and the heterogeneity of preeclampsia, good progress has been made in developing tractable animal models for this condition. These can be used to investigate some of the underlying mechanism and to evaluate potential interventions (495). The models of fetal growth restriction generally reduce nutrient supply to the placenta and fetus and developing strategies to ameliorate this remain challenging (518).

### 6.2 Animal models of gestational diabetes mellitus

Pregnancy is a state of insulin resistance where insulin’s response in target tissues, particularly (but not exclusively) muscle, liver, and fat, are modestly impaired. Maternal pancreatic β-cells adapt to pregnancy by expanding and consequently enhancing insulin production during the first two-thirds of pregnancy. As noted above, GDM is the result of β-cells failing to adapt to insulin resistance during pregnancy. One challenge with modelling GDM is that most animal models reproduce insulin resistance but not diabetes. Alternatively, the experimental manipulation is so severe that it causes the animal to produce overt β-cell dysfunction and thus diabetes develops before pregnancy and therefore these models may not truly reflect “gestational” diabetes, i.e. diabetes that only develops during pregnancy.

As pancreatic function is arguably the most important determinant of glucose homeostasis, manipulating pancreatic function either surgically, pharmacologically, or genetically, is the most direct way of inducing diabetes in pregnant animal models. As early as the early 20^th^ century, pancreatectomy was performed in dogs during pregnancy to show maternal hyperglycemia and glycosuria (519). However, it is unclear how the pregnant condition influenced the phenotype since these experiments were not performed in non-pregnant animals. Pancreatectomy is not regularly used because it is difficult to perform in small animals, not specific (both endocrine and exocrine tissues are removed) and the changes in the pancreas following surgery (e.g. pancreatic regeneration) are not necessarily related to diabetes.

A more commonly employed method of inducing β-cell dysfunction in rodents is the administration of streptozotocin (STZ), a glucosamine-nitrosurea and DNA alkylating agent which is selectively toxic to β-cells. The timing of STZ administration is an important factor, with treatment on the day of successful mating being the desired time point. STZ treatment before pregnancy can result in a severe diabetic phenotype pre-pregnancy impacting preimplantation embryo development (520). Conversely, STZ administration later in pregnancy (from mid-pregnancy onwards) does not fully interrogate β-cell adaptation as pregnancy-associated β-cell expansion reverts to its pre-pregnancy state in the third trimester of pregnancy (182). Moreover, STZ crosses the placenta and can potentially damage the developing fetal pancreatic β-cells (521).

Placental hormones including prolactin and placental lactogen play an important role in regulating β-cell expansion during pregnancy. Both prolactin and placental lactogen signal through prolactin receptors (PRLR) on β-cells to promote their growth. Given that PRLRs are expressed in key endocrine tissues and that *Prlr-*null mice are infertile, β-cell specific *Prlr* knockout models have been generated as models of GDM. Pancreatic β-cell specific *Prlr* knockout (βPRLRKO*)* female mice exhibit higher fasting glucose and impaired glucose tolerance when compared to controls but only during pregnancy and the glucose intolerance is reversed post-partum (522, 523). Moreover, while β-cell size was modestly reduced by the knockout in the non-pregnant state, pregnancy-induced β-cell expansion was entirely blunted. Notably, the fetal weights of βPRLRKO females mated with wildtype males were also increased. Taken together, this model recapitulates several key aspects of GDM observed in humans and support a role of placental hormones in mediating β-cell adaptations to pregnancy. However, it should be noted that, while these models allow us to investigate the impact of GDM on maternal physiology and feto-placental growth, the causal relationship between placental hormones and GDM remains unclear.

## 7 In vitro models for human placental dysfunction

There are significant differences in placental anatomy, cell types and molecular mechanisms regulating placental development between humans and common laboratory animals such as rodents. Thus, *in vitro* human trophoblast cell cultures provide value in that they allow us to study human placental development and function with direct species relevance and translational applications. A variety of *in vitro* trophoblast models including choriocarcinoma cell lines, transformed embryonic stem cells, isolated primary trophoblasts and placental explants have been used to study many aspects of trophoblast development and function. These methods allow pathological insults to be incorporated to model pathologies associated with placental dysfunction. While all models have certain advantages and disadvantages, none of them completely recapitulates the *in vivo* processes. Nevertheless, *in vitro* models can provide significant mechanistic insights into trophoblast biology. Overview of the advantages and disadvantages of *in vitro* models is provided in Table 4.

### 7.1 Choriocarcinoma and immortalized trophoblast cell lines

Early trophoblast culture studies relied on cell lines that were derived from malignant tumors of the trophoblast (choriocarcinomas). BeWo cells were the earliest trophoblast cell line derived in 1959, shortly after the publication of the first human cell line (HeLa) in 1952 (524, 525). BeWo cells were derived by serial passaging of choriocarcinoma deposits within the hamster cheek pouch (524). They are commonly used for endocrine and transport studies as they produce large quantities of placental hormones and maintain polarized plasma membranes with distinctly localized transporters resembling the syncytial epithelium (526). BeWo cells can be maintained in their undifferentiated state as cytotrophoblasts indefinitely or induced to differentiate into syncytiotrophoblast by elevating cAMP signaling, allowing several key processes of syncytialization to be modelled. The JEG-3 cell line is a sub-clone of BeWo but displays several extravillous trophoblast characteristics including expression of HLA-A and HLA-G. These extravillous trophoblast like properties allow JEG-3 cells to be used in studies of trophoblast invasion. However, unlike extravillous trophoblasts, HLA-G dimerization does not occur in JEG-3 cells and therefore prevents them from being recognised by decidual natural killer cells. JARs are another choriocarcinoma derived cell line that secretes placental hormones and can be induced to syncytialize in response to cAMP elevation.

Owing to the malignant phenotype of choriocarcinoma cells, researchers created immortalized cells from normal first trimester trophoblasts via transduction with the SV40 T-antigen which inhibits p53-mediated apoptosis and senescence. Two cell lines created using this method, HTR8/SVneo and Swan-71 were believed to phenotypically resemble primary cytotrophoblasts (527, 528). However, questions have been raised over the purity of these immortalized cell lines as they were shown to contain heterogenous cell populations (529). The process of immortalization also leads to significant mutations which can alter the phenotype from that of the primary cell.

The main advantages of choriocarcinoma and immortalized cell lines are that they can be easily propagated using standard culture protocols, grown indefinitely and are amenable to gene manipulation. However, there are several significant drawbacks to their use which have resulted in their loss of popularity in recent years. Choriocarcinoma and immortalized cell lines are intrinsically unstable in their genome and epigenome. Genome-wide profiling studies comparing these cell lines to primary trophoblasts demonstrate significant aberrations in their transcriptome and DNA methylome (530, 531). These cell lines are also aneuploids at the time of derivation and because of their genomic instability undergo frequent chromosomal rearrangements over many passages. Some of these lines have been in use for more than 5 decades, in multiple labs, under different conditions, which inevitably leads to genetic drift which can substantially alter their behavior from one another. Therefore, the reproducibility of experimental results from different labs using the same cell line remains an important concern. Another issue concerning choriocarcinoma or immortalized cell lines is that the role of biological variability cannot be addressed as each line originated from a single individual.

### 7.2 Human embryonic stem cell derived trophoblast-like cells

Studies in the last decade indicated that human embryonic stem cells (hESCs), but not mouse ESCs, can differentiate into trophoblast-like cells. Upon BMP4 activation and inhibition of FGF2 and TGFβ signaling, hESCs differentiate into trophoblast-like cells (532–535). These hESC-differentiated trophoblasts display several of the defining characteristics of the human trophoblast proposed by Lee et al. (536) including high expression of trophoblast genes TFAP2A/C, GATA2/3 and KRT7, as well as secretion of placental hormones placental lactogen and hCGα/β. HLA-G expression (extravillous trophoblast marker) also increased with BMP4 treatment but the expression of HLA-A and HLA-B (which are absent in all trophoblasts) has not been determined. hESC-derived trophoblasts differentiate into both syncytiotrophoblast and extravillous trophoblast -like cells with the same protocol, and thus a true trophoblast progenitor cannot be captured. Later studies characterizing hESC-derived trophoblasts indicated that they may in fact represent a population of mesoderm cells (537), most likely amnion epithelial cells which exhibit many similar characteristics to the trophoblast. Moreover, BMP4 promotes differentiation of human induced pluripotent cells into the amnion lineage (538, 539). The role of BMP4 in human trophoblast development is also questionable given that true human trophoblast stem cells and trophoblast organoid cultures (both of which are discussed below) do not require BMP activation (65, 108, 540).

### 7.3 Placental villous explants

Placental explant culture consists of small pieces of villous tissue (1-5 mm^2^) that are dissected from the placenta and cultured *in vitro* under similar conditions to cells. Placental explants retain the tissue architecture and heterogenous cell composition of the *in vivo* placenta compared to cultured cells. Explant cultures are typically short-lived which can be advantageous in some instances as the functional properties of the placenta are retained better than cultured cells which can lose their physiological characteristics over time in culture.

Trophoblast proliferation and migration can be examined by culturing first trimester villous explants directly on plastic dishes or membrane inserts, pre-coated with an ECM. Cytotrophoblast outgrowth then arises by attachment and migration of cells from cytotrophoblast cell columns into the ECM layer. Proliferation can be monitored either by sequential imaging and quantification of outgrowths over time in culture or staining with cell proliferation markers (e.g., Ki67 or BrdU). Explants cultured on collagen or Matrigel (mixture of structural proteins found in basement membranes) show cytotrophoblast outgrowth from cell columns as well as differentiation into extravillous trophoblasts and their migration (541). In contrast, there was no trophoblast proliferation or migration in explants cultured on agarose, despite no differences in viability (541), demonstrating the importance of the ECM to assess trophoblast properties.

Villous explants from term placentas are typically used to examine endocrine, transport and metabolic functions as well as syncytiotrophoblast differentiation. A major advantage of the term villous explant is that the functional properties of placentas affected by pregnancy complications can be examined alongside healthy controls. For example, preeclamptic villous explants show elevated sFLT-1/PlGF ratio compared to healthy placentas (542), recapitulating the *in vivo* situation (241). Placental explants have served as excellent models for the preclinical testing of pharmacological agents such as sildenafil (543), pravastatin (544), esomeprazole (545) and metformin (386) prior to their evaluation in clinical trials.

A major disadvantage of term villous explants is that they undergo syncytial degeneration within 24h of culture and tissue viability begins to decline after 72h (546). While some studies report syncytial regeneration after 72h (547), other parameters such as syncytial detachment (548), reduction in hCG secretion (547, 549, 550) and metabolic capacity decline (548) have been reported after 96-144h of culture, while oxidative stress markers increase from 120h. Additionally, gene targeting (e.g., siRNA transfection) of explants is restricted only to the outer syncytial layer (551, 552). While trophoblasts are the main cells under investigation, it is important to recognize that the villous explant contains many other cell types including stromal, endothelial, blood and immune cells. This cellular heterogeneity can give rise to both intra and inter-placental variation in treatment response. For example, hCG secretion is mediated by the syncytial portion of the villous explant which may vary with each tissue piece. While certain normalization procedures (such as tissue weight or protein concentration) can reduce the variability, determining how the differences in cellular composition of each tissue piece contributes to the response is difficult. Moreover, the individual cell type that produces the response under investigation is not determined easily.

### 7.4 Cultured primary trophoblasts

Cytotrophoblasts can be isolated from both first trimester and term placentas via digestion with enzymes such as trypsin, collagenase or dispase, and purified via density gradient centrifugation. Syncytiotrophoblast cannot specifically be isolated from placentas as the outermost layer is usually sloughed away during the enzymatic digestion (553). However, syncytiotrophoblast can contaminate cytotrophoblast cultures at the time of plating, but they do not attach well to tissue culture plastic and are washed away (554). Syncytiotrophoblast can be obtained by isolating and culturing cytotrophoblasts which spontaneously syncytialize in culture (553). Cytotrophoblasts cultured using this method do not require additional supplements except fetal bovine serum. Isolated term cytotrophoblasts have low proliferative activity, differentiate into syncytiotrophoblast within 3-4 days and can be maintained for up to 7 days in culture. Nevertheless, this method was a significant advancement in trophoblast studies as it allowed researchers to examine populations of primary trophoblasts with high purity (97-99%). Moreover, term placentas are more readily accessible than first trimester placentas in most labs and cytotrophoblasts can be isolated in large quantities allowing for multiple assays to be performed from each placenta.

Trophoblasts isolated from first trimester placentas are usually mixtures of villous cytotrophoblasts and extravillous trophoblasts (555, 556). When plated overnight on ECMs such as Matrigel or fibronectin, they exhibit an invasive phenotype and 70 – 90 % of these cells become HLA-G+ extravillous trophoblasts (556). Primary extravillous trophoblasts are commonly used to study trophoblast invasion but are also an excellent model for studying trophoblast immune interactions, particularly with decidual natural killer cells (557). Extravillous trophoblasts cultivated on ECM however remain in a non-proliferative state. Cytotrophoblasts from first trimester placentas isolated and cultured using this method are obtained at relatively low cell numbers and do not survive for long periods in culture. Therefore, frequent isolations are required from tissues that are not as easily accessible as the term placenta.

### 7.5 Human trophoblast stem cells

Although the mouse trophoblast stem cell line was established in 1998 (558), it was another 20 years before the first genuine human trophoblast stem cells (hTSCs) were derived by Okae and colleagues from the Arima lab (65). Previous attempts to isolate and culture hTSCs failed owing in part due to the lack of knowledge of the molecular mechanisms governing cytotrophoblast self-renewal. Okae et al initially examined the transcriptomes of primary trophoblasts from first trimester placentas to determine which molecular pathways were enriched in cytotrophoblasts compared to syncytiotrophoblast, extravillous trophoblasts and stromal cells. Based on these analyses and following further optimisation of culture conditions, Okae et al. found that activation of Wnt and EGF signalling and inhibition of TGF-β, histone deacetylases and ROCK were required for the long-term culture of hTSCs *in vitro*. hTSCs can be derived from first trimester placentas <10 weeks gestation as well as blastocyst stage embryos. These cells can be maintained indefinitely in culture and chromosomal analysis of hTSCs show that they are karyotypically normal after 150 cell doublings. Single cell transcriptomic analyses later demonstrated that hTSCs resemble the earliest cytotrophoblast progenitor population previously identified in first trimester placental tissues (54, 559). hTSCs are bipotent, capable of differentiating into syncytiotrophoblast upon cAMP induction or into extravillous trophoblasts by neuregulin-1 and TGF-β inhibition (65). The differentiation capacity of hTSCs was maintained even after 50 passages.

In addition to being true cytotrophoblast progenitors, hTSCs also provide other advantages. Gene manipulations can be performed relatively easily in hTSCs, allowing gene function to be studied at a high-throughput level. For example, a genome-wide CRISPR knockout screen was implemented to identify essential and growth-restricting genes in hTSCs (71). hTSCs cultured in Matrigel droplets with trophoblast organoid media develop into trophoblast organoids (559) that are indistinguishable from first trimester placenta derived trophoblast organoids (108, 540) (described below). Lastly, the Arima lab have deposited these cells onto a biorepository (RIKEN BioResource Research Center) allowing hTSCs to be easily accessed by many labs around the world without the need to isolate them from first trimester placentas.

### 7.6 Trophoblast Organoids

Trophoblast organoids are 3D cultures of human trophoblasts that retain the cellular architecture, function, and molecular characteristics of the *in vivo* villous trophoblast. In 2018, trophoblast organoids were derived from first trimester human placentas by two independent groups using similar methods (108, 540). Organoid cultures require trophoblast stem cells and therefore were initially derived from first trimester placentas <10 weeks gestational age (but see below for more recent work). The use of Matrigel droplets allows TSCs to develop into self-organizing 3D structures. Trophoblast organoids are cultured in media with similar requirements for growth factors and inhibitors as hTSCs, i.e., activation of Wnt and EGF and inhibition of TGF-β signaling. Organoids develop initially in small cell clusters and grow into spheroids of irregular shape. Trophoblast organoids however have an inverted architecture consisting of the syncytiotrophoblast core surrounded by cytotrophoblasts in the periphery that are in contact with the Matrigel. Extravillous trophoblasts can also be differentiated from trophoblast organoids. During differentiation, extravillous trophoblasts emerge from the cytotrophoblast region and migrate outwards digesting through the Matrigel until they reach the tissue culture plastic. Trophoblast organoids can be cultured continuously long-term (>1 year) without loss of differentiation capacity while remaining genetically stable (540, 560).

The 3D trophoblast organoids can be advantageous over the 2D hTSC cultures because it retains the cell-cell interactions and mechanical cues resulting from the complex structure, which may have physiological implications. For example, trophoblast organoids remained HLA-null unless when differentiated into extravillous trophoblasts, whereas hTSCs express HLA albeit at very low levels (561). Cell-cell interactions between trophoblasts and maternal cells may also be studied by combining for example, natural killer cells with extravillous trophoblast-differentiated trophoblast organoids.

Despite these advancements in trophoblast organoids, there are several limitations. Because hTSCs and trophoblast organoids are derived from placentas <10weeks gestational age, indefinite cultures of patient-specific models of many placental disorders (such as those discussed in this review) cannot be obtained given that these disorders are only diagnosed in the second half of pregnancy. However, recent studies demonstrate the feasibility of longer-term primary trophoblast 2D monocultures and 3D organoids from term placentas (562–564) allowing for future studies to model disease conditions.

The integration of endometrial stromal cells with ESC-derived blastocysts (termed “blastoids”) is a rapidly advancing field with significant potential for translating foundational studies of human embryo implantation. Similarly, we can anticipate that the integration of trophoblast organoids with endometrial organoids will enhance our understanding of trophoblast invasion, maternal-fetal communication, and early placental development. Notably, the use of endometrial organoids, which can be derived non-invasively from menstrual flow (565), offers a promising approach to studying the maternal contribution to defective placentation in preeclampsia and FGR when combined with trophoblast organoids.

Currently, integrated models of the endometrium and trophoblasts are in their early stages of development. Existing studies rely on transformed or carcinoma-derived trophoblast and endometrial cells rather than primary or stem cell models (566). Key challenges of integrating these models include synchronizing developmental stages of the different organoid systems before co-culture, establishing physiological polarity in both models and optimizing growth factor and signaling conditions to sustain the co-cultures.

## 8 Future directions for diagnostics

### 8.1 Omics and biomarker discovery

Novel diagnostic approaches have the potential to impact on multiple elements of care, including identifying women at high risk of complications in early pregnancy, screening women in later pregnancy to detect those who would benefit from enhanced monitoring, and informing decision making around the timing of delivery. Given the lack of detailed mechanistic understanding of the causes of pregnancy complications, it is likely that future identification of novel approaches will use platforms which analyze potential markers across a wide range of biological pathways, i.e. omics. We have previously reviewed the potential for analysis of different types of biological sample using different types of omic platform in the context of FGR (570) and similar approaches are likely to be important in other placentally related complications of pregnancy. An element of future research might be to focus these efforts directly on maternal blood. We and others have previously studied the placenta using RNA-seq to determine which transcripts were differentially expressed and then assess measurement of the corresponding protein as a predictor of the given disease (276). However, development of maternal plasma cell free RNA-seq now allows direct assessment of the placental transcriptome (251, 252). Similarly, development of proteomic methods now allows – in effect – performing ELISA quality assessment of protein levels on thousands of proteins in very small samples of serum, using the proximity extension assay (571). As commercial platforms using this technology include many of the currently available ELISAs, this provides an alternative to selecting candidate markers based on indirect evidence, such as differential placental expression of the corresponding mRNA in a given disease state. However, these platforms tend to have the common properties of generating very large numbers of data points on a single sample and being quite expensive. The latter property combines with the other practical consideration that it is expensive and time consuming to collect biological samples from pregnant women prior to manifestation of a given condition. These issues have the consequence that the datasets generated have many more predictors (p) than subjects (n), referred to as the ‘big p, little n’ problem. Hence, a major challenge when applying these methods to identify new diagnostic methods will be identifying true disease-associated signals within the noise of false positive signals arising from type 1 statistical error due to multiple testing.

Future work can address these challenges by creating resources of data and biological samples from prospective pregnancy cohorts in multiple different settings around the world. The strongest argument against false discovery and for clinical utility is confirming the diagnostic test accuracy of a new predictor in a separate population ideally with widely differing demographic and ethnographic characteristics. Another solution to the problem of false discovery is to analyze the original dataset using optimal methods. This could include characteristics of the study design such as obtaining serial samples from the same women which can enhance differentiation of signal from noise (570). It could also include characteristics of the analysis, such as rigorous use of methods to reduce false discovery, such as robust methods for false discovery correction when identifying candidate predictors and use of methods to prevent optimism, such as bootstrapping and cross validation, when assessing potential multivariate models (572). Furthermore, future work is likely to benefit from developments in enhanced computational methods. Machine learning approaches, such as Lasso, Elastic Net and Random Forrest regression are now widely employed. Artificial intelligence (AI) methods, such as convolutional neural networks and recurrent neural networks are likely to be an increasingly important feature of future work (573). A relative obstacle to the implementation of AI is the “black box” nature of prediction and a further enhancement is the use of explainable AI (xAI), a method which provides insight into the basis of a given AI prediction (574). Finally, there is also the possibility that the search for disease predictors could provide insights into disease mechanism. It could be argued that the search for predictors of disease is futile in the absence of disease modifying therapies to reduce the risk in women who screen positive. However, the secondary analysis of multi-omic datasets generated for biomarker identification could also lead to insights into the underlying pathophysiological processes and the identification of potential new therapeutics.

### 8.2 Combining biomarkers and imaging

Ultrasound plays a pivotal role in screening healthy pregnant women and management of complications, such as FGR. Meta-analyses of ultrasonic methods for assessing the risk that the baby is severely small actually show quite high diagnostic accuracy (486). However, the background risk that the baby is affected by FGR is low in a healthy pregnancy. As all screening tests act by multiplying the prior odds of disease in the given population by the likelihood ratio associated with the test being positive, the predictive strength of a positive test will always be lower when applied to low risk women. Consequently, most low risk women who have a suspected small for gestational age fetus by ultrasound are false positives and, in such cases, intervention in the form of early delivery will actually tend to make outcomes worse. This likely explains why implementation of routine ultrasound for all low-risk pregnant women at 30-34 weeks in France was associated with increased risks of neonatal morbidity (575). Confining the use of ultrasound to high risk women results in fewer false positives as the prior risk of FGR is higher, hence the positive predictive value is higher (570). However, many of the risk factors which cause women to be referred for an ultrasound scan, such as physical examination employing measurement of the symphyseal-fundal height, only modestly increase the risk of FGR, hence there continues to be a high false positive rate. It is likely that when new biomarkers are identified their screening utility will be less than ultrasound. However, clinically useful screening might be achieved by the combination of ultrasound and biomarkers, with very high positive predictive values when both are abnormal and very high negative predictive values when both are negative, as was demonstrated for a novel metabolite-based ratio for FGR (250). In that study, we demonstrated the positive likelihood ratio (LR) of an FGR infant was 4.8 with an ultrasound estimation of fetal size <10^th^ centile; a positive LR of 4.0 with an elevated metabolite-based ratio, but a positive LR of 23.9 for pregnancies where both parameters were abnormal. (250) Hence one potential future model of care is that women are selected for ultrasonic surveillance of fetal growth following risk stratification using maternal blood biomarkers.

## 9 Future directions for therapeutics

Technological advances have led to big breakthroughs rendering many serious diseases treatable - melanoma, other cancers, and even rare genetic conditions. Unfortunately, placental conditions have not benefited: there is still no clinical therapeutic agent that targets the placenta to treat serious pregnancy conditions. It has been proposed that aspirin prevents preterm preeclampsia by improving placental implantation. While this may be true, preclinical data supporting this is sparse - it is plausible aspirin prevents preeclampsia simply by improving maternal vascular function without affecting placental pathology. Barriers to the development of new drugs to treat pregnancy conditions include conservatism by regulatory authorities (who need to consider adverse effects on fetal health) and lack of pharmaceutical company interest in the field of pregnancy (576). This section covers future directions for therapeutics that aim to exert a biological effect on the placenta; either directly, or indirectly by improving perfusion to the placenta. There are broadly two approaches. The first is to repurpose drugs that already exist and use them to treat placental conditions. The second is to generate entirely new drugs, such as molecular targeted therapies.

### 9.1 Conceptual challenges to developing new therapeutics targeting the placenta

Generating drugs to improve placental health could either aim to act directly on the placenta, or indirectly, such as dilating the uterine arteries to improve placental perfusion. Drugs that target the placenta face conceptual challenges. Most drugs are proposed to biologically act on the syncytiotrophoblast, a layer that undergoes constant shedding (418, 577). This may be a challenge as therapeutic agents designed to act on the placenta need to accumulate in the syncytiotrophoblast at high enough concentrations to exert a biological effect even though a portion of the layer is shed. Furthermore, shedding of this layer is exacerbated in the placental diseases with an unmet need for novel therapies, notably preeclampsia (418). This may be a particular challenge for mRNA-based therapeutics which can have long lasting actions on cells that do not rapidly turn over (such as hepatocytes). By targeting the placenta, any drug effect may be lost when the syncytiotrophoblast containing the mRNA is shed from the placenta.

Secondly, it may be difficult to even get sufficient amount of drug into the placenta to exert a biological effect. It is helpful that 20% of the maternal circulating blood volume is at the maternal fetal interface. However, it remains unclear whether many small molecules which are candidate treatments for preeclampsia can cross the constantly shedding syncytiotrophoblast to reach therapeutically beneficial levels.

The third challenge is the ever-present concern for fetal safety that applies to any proposed treatment for pregnancy. This presents translational barriers. Drug approving agencies such as the Food and Drug Administration (FDA) are more conversative in approving drugs for human use when it applies to a pregnant cohort. Pharmaceutical companies tend to shy away from developing drug pipelines for pregnancy conditions (576) as they often perceive risk may not overcome the potential for commercial benefit.

### 9.2 Repurposed small molecule drugs

Most proposed treatments for placental conditions have applied the approach of repurposing drugs, mostly small molecules. This means determining whether drugs already licensed for other indications may be effective in treating placental conditions. While repurposing existing drugs is less conceptually interesting then conceiving a new drug from scratch, it is pragmatic. Extensive human safety data will already exist. Often safety data on pregnant populations may also exist because the drugs are used to treat co-existing medical conditions that are present during the pregnancy. This means promising ‘repurposed drugs’ identified preclinically can be fast tracked to clinical trials. In contrast, studies of a new entity are expensive, time consuming and exhibit a high level of attrition (i.e. compounds that are evaluated but never make it to a Phase 3 study). Repurposing drugs therefore represents the most direct pathway where new drugs for obstetric conditions may be found.

There are a large number of drugs proposed to counter the placental pathophysiology of preeclampsia in either cellular models, or in animal models. They include metformin (386), pravastatin (544, 578), proton pump inhibitors (545), melatonin (579) and resveratrol (580). These drugs are variously reported to reduce placental secretion of sFLT1 or soluble endoglin, vasodilate, dampen inflammation or up-regulate cellular molecules that mount an anti-oxidative response.

A number of these drugs have been evaluated in clinical trials and these have yielded mixed results. In randomized trials using these drugs to treat preterm preeclampsia (where the primary outcomes were either prolongation of gestation or reduction circulating concentrations of sFLT1), esomeprazole was negative (581), pilot trials of pravastatin yielded mixed results (582, 583) and sildenafil was found to be potentially useful (584). Unfortunately, fetal safety concerns over repeated antenatal dosing of sildenafil (585) have made it unsuitable for further consideration in the treatment of preeclampsia. A large trial of 20 mg of pravastatin commenced at 36 weeks to prevent preeclampsia was negative (586) but did not raise concerns about fetal safety.

Metformin may have promise in treating preeclampsia. In a randomized trial of 180 women with preterm preeclampsia (the Preeclampsia Intervention 2 trial, PI2), metformin prolonged pregnancy by around a week (median prolongation 7.6 days; geometric mean ratio 1.39 which means a 39% prolongation (95% confidence interval 0.99-1.95), p=0.057) (587). Although the intention to treat analysis was borderline non-significant in that trial, planned sub-analyses showed prolongation in gestation was significant among those who managed to take the trial medication, a 11.5 day prolongation.

While most attention has been on preeclampsia, other conditions have been evaluated. The Strider consortium was a series of concurrent randomized trials to evaluate whether sildenafil citrate (a selective phosphodiesterase inhibitor which may cause placental vasodilation and increased uteroplacental perfusion) could rescue severe FGR. The Strider UK reported a negative finding (588), Strider NZAus also reported a negative finding (with possibly a few encouraging trends towards benefit) (589) and the Netherlands consortium also reported a negative finding and raised potential concerns that the drug may cause fetal pulmonary hypertension (585).

Sildenafil has also been evaluated in labor. A randomized trial of 300 women found sildenafil given during labor reduced the incidence of operative birth by 51%, with numbers needed to treat of only 5. Furthermore, it seemed to reduce rates of pathologic fetal heart rate patterns by 43% (590).

### 9.3 Nanoparticles, siRNAs and other molecularly targeted therapies

Preclinical studies have explored the possibility of targeting drug delivery to the placenta by loading the drug into nanoparticles (591). Placental targeting is achieved by studding the surface of the nanoparticles with a molecule that preferentially binds the placental surface (such as a peptide sequence (592), or an antibody that binds the epidermal growth factor receptor, as this receptor is highly expressed on the placental surface (593). Nanoparticle approaches may maximize drug concentration to the placenta and minimize off target effects (591). There have been no clinical trials examining nanoparticle delivery of drugs to the placenta, and the concept is in its infancy with respect to potential clinical translation.

There have been a limited number of molecular therapies targeting placental function that have been proposed or even tested in clinical trials. Given placental development is highly dependent on epidermal growth factor receptor signaling, gefitinib (a small molecule inhibitor of this pathway) has been evaluated as a potential medical treatment for ectopic pregnancy. While preclinical and early phase (single arm) trials appeared encouraging (594–596) a large phase III multi-center trial yielded a negative result (597).

Injecting an adenoviral vector into the maternal uterine arteries that is designed to express VEGFA into the endothelium has been proposed as a novel approach to rescue severe FGR arising from poor placental function. This approach increases uterine artery blood volume (598, 599) and causes the release of nitric oxide to induce vasorelaxation (598). It increases fetal growth in sheep (600) and guinea pigs (601). The EVERREST consortium is a UK based group that has attempted to translate this into humans. They published a protocol describing a prospective observational study of pregnancies with early onset FGR to help define the potential target population that may benefit from such a treatment (602). It is unclear whether human trials are imminent or will happen (there is no trial registered). Regulatory hurdles in translating this concept are likely significant but these and the ethical issues have been considered (603).

siRNAs to silence genes to treat preeclampsia have been evaluated. A preclinical report concluded an siRNA reducing maternal hepatic angiotensinogen expression rescued a preeclampsia phenotype in two animal models and even improved placental morphology (604). Angiotensinogen is a peptide that is the precursor to angiotensin II, which signals through its cognate receptor on endothelial cells to promote vasoconstriction. Angiotensinogen is a good molecular target. As it is produced in the liver, an siRNA that targeting this protein does not need an organ specific targeting mechanism because an intravenous injection will passively reach the liver. Zilebesiran is an siRNA drug targeting angiotensinogen (Alnylam Pharmaceuticals) shown in a phase I study to elicit a sustained drop in blood pressure in a non-pregnant cohort lasting 24 weeks (605). Given the likely role of the renin-angiotensin system in the vascular pathology of preeclampsia, it possible this drug could be effective in treating preeclampsia. Unfortunately, no trials of this drug have been registered. Unfortunately, it appears the drug was initially generated to treat preeclampsia but in 2019, the company pivoted towards treatment of chronic hypertension (606).

Turanov et al proposed siRNA targeting sFLT1 as an approach to treat preeclampsia (607). They evaluated a mix of siRNAs targeting the sFLT1-i13a and sFLT1-e15a isoforms (both upregulated in placenta with preeclampsia (608) without silencing the full-length vascular endothelial growth factor receptor (encoded by the same gene). When administered to mice, most of the siRNAs accumulated in the liver and kidney but 7% reached the placenta and this seemed sufficient to reduce circulating sFLT11 by around 40%. Reassuringly, the labelled siRNA was not detected in the fetus. A single injection of the siRNA in a baboon model of preeclampsia (single uterine artery ligation, n=3) trended towards reduced blood pressure and proteinuria. There was a potential trend towards the offspring being born at a reduced birthweight but numbers were very small. It is encouraging to note that there is an effort to translate this novel treatment for preeclampsia. Comanche Biopharma have announced they have raised $75 million (US) completed first in human (non-pregnant) trials and planning to undertake phase I trials in women with preeclampsia (609).

## 10 Conclusions

Normal function of the placenta is essential for an uncomplicated pregnancy and a healthy offspring. Placental dysfunction is a direct and indirect cause of a substantial burden of global morbidity and mortality. Despite this, there is a lack of mechanistic understanding of the causes of most of the serious pregnancy complications, there is a limited and relatively poorly performing repertoire of tests to assess placental function clinically, and there is an almost complete absence of disease modifying therapies for the common complications of pregnancy. Developing better understanding of normal and abnormal placental function should, therefore, be identified as a research priority. The major areas for improving clinical management of placentally-related complications of human pregnancy are better screening tests and diagnostics (both imaging and biochemical) and the development of disease modifying therapies. Given the limitations of animal models, detailed prospective studies of optimally phenotyped pregnancies, both healthy and complicated, could yield the data to develop better prognostic and diagnostic tests. Application of high dimensional computational methods (machine learning and AI) to multilayered omic datasets arising from such studies could yield both novel tests and also help identify the mechanistic pathways involved. Hypotheses arising from these studies could then be tested using novel cellular model systems, employing human stem cells and organoids, which have developed considerably over recent years. The combination of a greater focus on pregnancy complications as an area of unmet need with recent technological developments in omics, imaging and in vitro models could lead to prevention of the tragic and long-term sequelae of placental dysfunction and the better targeting of high risk care to the women who need it most.

## Figures and Tables

**Table 1:** Placental histopathology findings in stillbirths in relation to small for gestational age (SGA), large for gestational age (LGA) birthweight, or neither.

Associated with stillbirth AND SGA/LGA	Associated with stillbirth BUT NOT SGA/LGA
single umbilical artery	acute chorioamnionitis (membranes)
velamentous insertion	acute chorioamnionitis (chorionic plate)
terminal villous immaturity	chorionic plate vascular degenerative changes
retroplacental hematoma	fibrinoid deposition
parenchymal infarction	fetal vascular thrombi in the chorionic plate
Intraparenchymal thrombus	
avascular villi	
placental edema	
birth weight/placental weight ratio	

Data abstracted from Bukowski et al 2017 (289)

**Table 2:** Maternal and fetal vascular malperfusion as defined by the Amsterdam Placental Workshop Group (284). *Karyorrhexis – fragmentation of the nucleus and breakup of chromatin into unstructured granules. ** Ectasia – dilation or distention of tubular structure.

Macroscopic and histologic changes associated with fetal growth restriction
**Maternal vascular malperfusion**
**Gross changes**
Placental hypoplasia (placental weight <10^th^ centile)
Thin umbilical cord (<10^th^ centile)
**Vascular lesions**
Acute atherosis/fibrinoid necrosis
Persistent muscularization of basal plate arterioles
Thrombosis in basal decidual vessels
Mural hypertrophy of membrane arterioles
**Villous changes:**
Villous infarction
Retroplacental hematoma
Accelerated villous maturation
Distal villous hypoplasia
**Fetal vascular malperfusion**
Thrombosis (in fetal vessels)
Avascular villi
Intramural fibrin deposition (in fetal vessels – subendothelial, or intramural)
Villlous stromal vascular karyorrhexis*
Stem vessel obliteration
Vascular ectasia**
**Other**
Non-infectious villitis/villitis of unknown etiology

**Table 3:** Key clinical and pathological differences between preterm (diagnosed <37 weeks gestation) and term preeclampsia (diagnosed >37 weeks gestation).

	Features that are increased with preterm preeclampsia, compared to term preeclampsia
Pathological	Frequency of placental lesions, such as maternal, or fetal vascular malperfusion lesions (370)
	Co-existing fetal growth restriction (371)
	Uterine artery Doppler abnormalities in the first trimester (372)
	Low circulating PIGF and PAPP-A in the first trimester (372)
Clinical	Prevalence of serious maternal and fetal morbidity, including maternal death (363)
	Risk of long-term cardiovascular disease (373)
	More accurately predicted during early pregnancy using the Fetal
	Medicine Foundation Algorithm (372)

**Table 4:** Summary of *in vitro* human trophoblast models

*In vitro* model	Origin / derivation	Benefits	Limitations	First reported
BeWo	Chorio-carcinoma	Indefinite culture Simple culture protocols Tractable model of differentiation (STB) Amenable to gene targeting	Cancer cells Aberrant genome and epigenome Genetic drift over many passages Limited differentiation capacity (STB only) Limited physiological relevance	Hertz *et al.* 1959 (524)
JAR	Chorio- carcinoma	Indefinite culture Simple culture protocols Tractable model of differentiation (STB) Amenable to gene targeting	Cancer cells Aberrant genome and epigenome Genetic drift over many passages Limited differentiation capacity (STB only) Limited physiological relevance	Pattillo *et* *al.* 1968 (567)
JEG3	Chorio-carcinoma, sub-clone of BeWo	Indefinite culture Simple culture protocols EVT model without requirement for differentiation Amenable to gene targeting	Cancer cells, Aberrant genome and epigenome Genetic drift over many passages Post-differentiation cells Limited physiological relevance	Kohler *et al.* 1971 (568)
Term CTBs	Isolated and purified from term placentas	Primary cultures Simple culture protocols Physiologically relevant Model of differentiation (STB)	Limited time in culture Long process to isolate cells Undergo spontaneous differentiation into STB Limited scope for gene targeting	Kliman *et al.* 1986 (553)
First trimester CTBs	Isolated and purified from first trimester placentas	Primary cultures Simple culture protocols Physiologically relevant Model of differentiation (EVT)	Limited time in culture Mixtures of CTBs and EVTs Requires access to restricted and limited tissues (first-trimester placentas) Limited scope for gene targeting	Yagel *et al.* 1989 (569)
HTR8/ SVneo	Immortalised first- trimester placentas	Indefinite culture Simple culture protocols More closely related to first-trimester CTBs than choriocarcinoma derived cell lines Amenable to gene targeting	Transformed cells Unpure populations of trophoblasts containing stromal cells Aberrant genome and epigenome Genetic drift over many passages Limited physiological relevance	Graham *et* *al.* 1993 (527)
Swan-71	Immortalised 7-week CTB isolate	Indefinite culture Simple culture protocols More closely related to first-trimester CTBs than choriocarcinoma derived cell lines Amenable to gene targeting	Transformed cells Unpure populations of trophoblasts containing stromal cells Aberrant genome and epigenome Genetic drift over many passages Limited physiological relevance	Straszewski- Chavez *et* *al.* 2009 (528)
hESC/hiPSC-derived trophoblast-like cells	Human ESCs or iPSC lines	Relatively simple protocol to differentiate into trophoblasts Differentiates into both lineages (EVT and STB)	Limited time in culture Requires access and expertise in ESC/hiPSC culture techniques Differentiation is non-tractable Questionable whether these are true trophoblasts	Li *et al.* 2013 (534)
hTSCs	Isolated and purified from human blastocysts and first trimester placentas	Indefinite culture Pure trophoblast stem cells Tractable model of differentiation (EVT and STB) Can be transformed into 3D organoids Physiologically-relevant Amenable to gene targeting	Complex culture protocols Requires access to restricted and limited tissues (first-trimester placentas) if deriving cell lines in the lab. Limited biological replicates available if ordering from biorepository	Okae *et al.* 2018 (65)
Trophoblast organoids	Isolated trophoblasts from first trimester placentas and grown in special conditions	Indefinite culture 3D culture may be more physiologically representative than 2D Physiologically-relevant Can model trophoblast cell-cell interactions. Differentiates into both lineages (EVT and STB)	Complex isolation and culture protocols Requires access to restricted and limited tissues (first-trimester placentas). Limited gene targeting. Considerable inter and intra-placental variability	Haider *et al.* 2018 (108) Turco *et al.* 2018 (540)

## Clinical Highlights

- The placenta profoundly alters maternal physiology during pregnancy, in part due to the effects of placentally-derived hormones released into the maternal circulation. Recognition that one of these, GDF15, is likely to be the cause of hyperemesis gravidarum indicates that therapies targeting this pathway could be effective in treating the condition.
- Placental dysfunction is a major determinant of preeclampsia, fetal growth restriction, spontaneous preterm birth and stillbirth, hence it contributes significantly to the global burden of disease, principally through perinatal morbidity and mortality, but also through maternal death due to complications such as preeclampsia and placental abruption.
- Current clinical methods for selecting women for enhanced surveillance during pregnancy for placentally-related complications perform poorly. Better understanding of the causes and consequences of placental dysfunction could lead to novel tests which allow better stratification of care. Novel methods are likely to include further development of ultrasound, use of MRI, and biomarkers identified by omics studies of large, prospective cohort studies.
- Mechanistic understanding of disease pathophysiology could also lead to novel therapies, but risk aversion and the difficulty in proving long term safety of novel treatments may still present obstacles, even if disease mechanism was understood and candidate treatments were available.
